# Mapping the Evolving AI Preferences and Care Needs in Orthopedic Transitional Care From Hospitals to Home: Cross-Sectional Study

**DOI:** 10.2196/91061

**Published:** 2026-08-04

**Authors:** Xiaomin Huang, Xiaoqiong Peng, Weiling Zhang, Meng Zhou, Qingtang Zhu, Tianwen Huang

**Affiliations:** 1 Department of Musculoskeletal Oncology, The First Affiliated Hospital, Sun Yat-sen University, Guangzhou, Guangdong, China; 2 Department of Spine Surgery, The First Affiliated Hospital, Sun Yat-sen University, Guangzhou, Guangdong, China; 3 Department of Microsurgery, Trauma andHand Surgery, The First Affiliated Hospital, Sun Yat-sen University, Guangzhou, Guangdong, China; 4 Department Of Orthopedic,Trauma & Microsurgery, The First Affiliated Hospital, Sun Yat-sen University, No. 58 Zhongshan Road, Guangzhou, Guangdong, 510080, China, 86 18902233835

**Keywords:** AI, orthopedics, transitional care, patient preference, rehabilitation, digital health

## Abstract

**Background:**

Enhanced recovery after surgery protocols have shortened orthopedic hospital stays but have shifted rehabilitation and safety-monitoring tasks to patients and families after discharge. In this study, AI refers to patient-facing digital systems for orthopedic transitional care, including large language model chatbots, computer vision or platform-based monitoring tools, and wearable sensor–enabled systems for education, rehabilitation guidance, motion correction, and risk alerts. However, patient-reported preferences for different AI-supported functions across the hospital-to-home transition remain underexplored.

**Objective:**

This study aimed to map the evolution of care needs from hospital to home recovery and to identify specific preferences and factors associated with the willingness to use AI systems in orthopedic transitional care.

**Methods:**

We conducted a multicenter cross-sectional survey among patients recovering from orthopedic surgery in 33 Guangdong hospitals, China. Of the 860 submitted questionnaires, 752 responses were included after prespecified quality control, including exclusion of responses completed in 180 seconds or less based on pilot-informed screening. Participants rated standardized function-based AI descriptions rather than a specific prototype or live tool. The data covered demographic and clinical characteristics, task priorities across care phases, perceived transitional care challenges, and stated willingness to use AI. The survey was informed by the technology acceptance model, although perceived usefulness, perceived ease of use, and attitude toward use were not directly measured. Exploratory factor analysis was used to examine perceived challenges. Descriptive mapping summarized care needs and AI function preferences, and multivariable logistic regression explored factors associated with willingness.

**Results:**

Most respondents reported a willingness to use AI (604/752, 80.3%). Care priorities varied by phase: inpatient priorities were more often related to information acquisition and care instruction, whereas home-stage priorities more often involved functional safety, rehabilitation guidance, motion correction, and risk alerts. Exploratory factor analysis identified 3 perceived challenge dimensions: home rehabilitation self-management barriers, lack of professional support, and symptom uncertainty. In adjusted exploratory analysis, willingness to use AI was associated with older age (adjusted odds ratio [aOR] 1.02, 95% CI 1.00‐1.03), comorbidities (aOR 1.72, 95% CI 1.09‐2.69), later rehabilitation stage (aOR 1.28, 95% CI 1.01‐1.62), and urban residence (aOR 1.85, 95% CI 1.14‐3.01). Unmarried, divorced, or widowed status was associated with lower willingness than married status (aOR 0.59, 95% CI 0.39‐0.89). Physical disability and self-care ability were not independently associated with willingness after adjustment.

**Conclusions:**

In this hospital-based convenience sample, most respondents were willing to use AI, and their stated priorities shifted from information support during hospitalization to functional safety and rehabilitation support after discharge. The associated factors should be interpreted as exploratory associations rather than causal determinants. Because participants evaluated function-based AI descriptions rather than actual AI tools, these findings can inform future prototype development and real-world evaluation, particularly around usability, trust, privacy, digital accessibility, and clinician oversight.

## Introduction

### Background

Recent data from the Global Burden of Disease Study 2023 highlight a substantial increase in life expectancy and a corresponding rise in age-related disease burdens [1]. This transition is driving increasing demand for orthopedic interventions, particularly given the persistent burden of fractures [2] and the rising prevalence of osteoarthritis [3]. While enhanced recovery after surgery protocols have significantly shortened the length of stay [4], they have also transferred substantial care responsibilities to the home environment. This “transitional care gap” exposes patients recovering from orthopedic surgery, especially older adults, to risks such as undetected functional decline [5] and fall-related adverse outcomes [6], indicating a critical need for innovative digital solutions.

Successful orthopedic recovery relies heavily on continuous, high-quality postdischarge management. Recent evidence demonstrates that implementing continuity of care postdischarge significantly improves functional outcomes, including gait stability, pain levels, and balance ability, among older adults undergoing arthroplasty [7]. However, unlike pharmacotherapy, improving function, strength, and mobility in patients recovering from orthopedic surgery requires active physical participation—specifically through correct execution of rehabilitation exercises and strict adherence to movement precautions [8]. Yet, without professional supervision, patients recovering at home frequently face execution barriers, such as kinesiophobia, incorrect exercise techniques, and the inability to detect early signs of complications such as deep vein thrombosis or infection.

AI has been proposed as a potential supportive approach to help address this gap. In the present study, AI refers to patient-facing digital systems for orthopedic transitional care, including large language model (LLM)–based chatbots for education and question answering, as well as computer vision, monitoring platforms, and wearable sensor tools for rehabilitation guidance, motion correction, and safety alerts. In this study, these AI modalities were presented to participants as standardized function-based descriptions rather than as an actual prototype, interface, or live AI system. In the hospital phase, LLMs such as ChatGPT have demonstrated the ability to answer patient questions regarding total knee arthroplasty [9,10], hip arthroscopy [11,12], and shoulder arthroplasty [13], with additional evidence supporting improved comprehensibility of AI-generated patient responses [14]. For example, Gemini has shown superior clarity in addressing clinical guidelines for anterior cruciate ligament reconstruction [15]. However, as patients transition to home recovery, their needs may extend beyond text-based information.

Despite significant technological advances, current orthopedic AI development often prioritizes technical feasibility over patient-centered design that reflects patients’ needs and preferences. A multinational survey suggests that while patients are generally open to AI, their trust remains fragile and depends heavily on physician oversight [16]. However, limited evidence has mapped how patient-reported needs change across the hospital-to-home transition and how these needs correspond to stated preferences for AI-supported functions. Understanding these stated preferences may help identify candidate functions for future prototype development and real-world evaluation, while avoiding the assumption that stated willingness necessarily translates into actual adoption or clinical effectiveness.

### Objective

This study was conceptually informed by the technology acceptance model (TAM), but it was not designed to test the full TAM pathway. Standard TAM emphasizes perceived usefulness and perceived ease of use; however, these constructs and attitude toward use were not directly measured in this exploratory survey. Instead, we examined perceived transitional-care challenge dimensions as contextual factors that may be relevant to stated willingness to use AI-supported functions. These dimensions should, therefore, be interpreted as contextual orthopedic transitional-care challenges rather than as core TAM constructs. Accordingly, this study sought to (1) describe patient-reported task priorities across the hospital-to-home transitional-care continuum; (2) explore the dimensional structure of perceived transitional-care challenges; and (3) identify demographic, clinical, and contextual factors associated with stated willingness to use AI-supported functions.

## Methods

### Study Design

A multicenter, quantitative, cross-sectional survey was conducted between August 25, 2025, and September 22, 2025, in Guangdong Province, China. The study adhered to the CHERRIES (Checklist for Reporting Results of Internet E-Surveys; Checklist 1) guidelines, the reporting on health equity in observational research (STROBE-Equity [Strengthening the Reporting of Observational Studies in Epidemiology—Equity]) guidelines (Checklist 2) [17], and the consensus-based Checklist for Reporting of Survey Studies (CROSS; Checklist 3) [18].

### Ethical Considerations

The study protocol was approved by the institutional review board of The First Affiliated Hospital, Sun Yat-sen University (approval number 2024‐244). The study was conducted in accordance with the Declaration of Helsinki. Electronic informed consent was obtained from participants who completed the questionnaire independently on the online platform. Verbal informed consent was obtained immediately before researcher-assisted completion for participants with limited digital literacy or reading difficulties. In these cases, trained researchers provided a standardized study explanation and entered patients’ responses only after consent had been obtained.

### Participants and Setting

The target population comprised patients who had recently undergone orthopedic treatment. Participants were recruited from 33 medical institutions using convenience sampling, including 30 tertiary hospitals and 3 secondary hospitals. These sites were strategically selected to include both the economically developed Pearl River Delta and peripheral regions, with the aim of capturing patients from urban and rural settings. However, because this was a convenience sample and most participants were recruited from tertiary hospitals, the sample should not be considered population-representative.

The inclusion criteria were as follows: (1) a diagnosis of an orthopedic condition, such as spine, joint, trauma, or sports-related injuries; (2) currently in the perioperative or recovery phase, up to 6 months after discharge; and (3) willing to participate. Exclusion criteria included severe cognitive impairment or critical clinical instability, such as admission to an intensive care unit, which prevented effective communication. Lack of digital literacy was not an exclusion criterion; assisted data collection was provided to improve the inclusion of participants who had difficulty completing the online questionnaire independently.

### Sample Size

The sample size was determined a priori for multivariable logistic regression using an events-per-variable (EPV) approach, with the target guided by commonly used recommendations for model stability and overfitting control [19]. Considering approximately 16 potential independent variables, and because no prior study had reported a directly applicable proportion of willingness to use AI in orthopedic transitional care, we prespecified a planning proportion of 50% for the binary outcome. This proportion was used as a neutral assumption for the initial EPV-based sample size calculation in the absence of directly comparable prior data, rather than as an empirical estimate of the true acceptance rate. Accordingly, a minimum sample size of 320 participants was required (16 × 10 / 0.50). To account for potential invalid responses commonly observed in orthopedic survey populations, the target recruitment was set at 600. Ultimately, 860 questionnaires were submitted, and after quality control screening, 752 valid responses were included in the final analysis. With 148 participants in the smaller outcome group (unwilling), the final EPV was approximately 9.3, which was considered close to commonly used rule-of-thumb thresholds for exploratory multivariable estimation [20].

### Questionnaire Development and Pretesting

#### Questionnaire Structure and Content

A self-developed questionnaire titled “Perceptions and Needs for AI in Orthopedic Transitional Care” was designed based on a comprehensive literature review and the TAM [21]. In this study, the term AI was defined for participants as patient-facing digital tools that could provide health education, online question answering, rehabilitation guidance, motion correction, symptom or risk monitoring, and alerts during the transition from hospital to home. No specific commercial prototype, interface, wearable device, monitoring platform, or live AI system was shown. Instead, participants rated standardized function-based descriptions so that responses reflected stated willingness toward AI-supported functions rather than actual use of a specific product.

TAM was used as a conceptual framework rather than as a complete measurement model. The questionnaire did not include validated multi-item measures of perceived usefulness, perceived ease of use, or attitude toward use. Therefore, this study should be interpreted as TAM-informed rather than as a full empirical test of TAM.

A multiprofessional panel comprising orthopedic surgeons, nurses, and digital health experts reviewed the draft questionnaire for content validity. Subsequently, a pilot study was conducted with 30 patients recovering from orthopedic surgery, who were excluded from the final analysis to assess clarity and feasibility. Based on their feedback, minor linguistic adjustments were made to improve comprehensibility.

The final questionnaire consisted of the following 5 sections as detailed below.

#### Demographic and Clinical Profile

This section collected information on age, sex, education level, marital status, residence, living arrangement, employment status, medical diagnosis, comorbidities, physical disability, self-care ability, and rehabilitation stage. Recruitment site information was also recorded, from which the hospital level was derived.

#### Transitional Care Tasks

Participants identified the top 5 priority tasks during the inpatient and home phases, respectively.

#### Perceived Challenges Scale

A 17-item scale assessed execution barriers, care support challenges, and symptom-related uncertainty during transitional care. These items were conceptualized as contextual transitional-care challenges that may be relevant to the stated willingness to use AI-supported functions within a TAM-informed framework. They should not be interpreted as direct measures of perceived usefulness, perceived ease of use, or other core TAM constructs. The factor analysis was conducted to examine the structure of perceived transitional-care challenges rather than to validate the structure of TAM. Items were rated on a 5-point Likert scale ranging from 1 (“strongly disagree” or “no difficulty”) to 5 (“strongly agree” or “extreme difficulty”).

#### AI Willingness

A single 5-point Likert-scale item measured the stated willingness to use AI systems. This item was used as an operational indicator of behavioral intention in this TAM-informed exploratory framework. For analysis, responses indicating “willing” or “very willing” were classified as willing, while the remaining responses were classified as not willing.

#### Barriers to Adoption

To map potential barriers to AI adoption, we administered a multiple-choice checklist. The items addressed key themes, including usability barriers, such as “operation is too complicated” and “interface information overload”; trust and security concerns, such as “data privacy risks,” “unreliable content/ads,” and “skepticism about online diagnosis”; and economic or habitual factors, such as “fear of unreasonable charges” and “preference for offline visits.”

### Data Collection Procedure

The survey was hosted on the Wenjuanxing platform (Changsha Ranxing Information Technology Co, Ltd). To accommodate the varying digital literacy levels of the orthopedic population, a hybrid data collection strategy was used.

#### Self-Administration

Survey links and QR codes were disseminated to eligible patients with digital proficiency in orthopedic wards and outpatient clinics.

#### Assisted Self-Administration

For participants with limited digital literacy, reading difficulties, or difficulty operating a digital device independently, trained researchers conducted face-to-face assisted questionnaire completion. Researchers read the questions aloud using a standardized neutral script and entered responses via tablet devices. Researchers were instructed not to interpret items for participants or suggest answers. This assisted-entry approach was used to improve inclusiveness; however, the administration mode of the questionnaire was not retained as a separate analyzable variable in the final dataset. Therefore, the independent effect of assisted completion on stated willingness could not be evaluated.

To ensure data quality, IP address restrictions were enabled to reduce duplicate submissions. An automated response-time check was performed, and questionnaires completed in 180 seconds or less were excluded. Invalid data were defined as questionnaires submitted with a completion time of 180 seconds or less, based on the prespecified automated response-time quality control criterion. This threshold was set a priori according to the 30-patient pretest and the number of survey items, because responses completed at or below this threshold were considered unlikely to reflect adequate reading and deliberation. Accordingly, 108 submitted questionnaires were excluded from the analysis, and 752 valid responses were included in the final analysis. All data were deidentified and stored on an encrypted server accessible only to the principal investigator.

### Statistical Analysis

Data were exported from Wenjuanxing and analyzed using SPSS (version 26.0; IBM Corp). Continuous variables were assessed for normality using the Shapiro-Wilk test; normally distributed data were presented as mean (SD), while nonnormally distributed data were reported as median and IQR. Categorical data were summarized as frequencies and percentages (%).

The psychometric properties of the Perceived Challenges Scale were evaluated using Cronbach α for internal consistency and exploratory factor analysis (EFA) for construct validity. EFA was used to examine the dimensional structure of perceived transitional-care challenges rather than to validate TAM constructs.

To explore factors associated with the stated willingness to use AI, binary logistic regression analysis was performed. Willingness to use AI was dichotomized from the 5-point Likert-scale item, with “willing” and “very willing” coded as willing and the remaining responses coded as not willing. Odds ratios (ORs) with 95% CIs were calculated. Multivariable logistic regression was conducted as an exploratory association analysis rather than as a predictive modeling exercise. Given the cross-sectional design, adjusted odds ratios (aORs) were interpreted as associations and not as evidence of causal effects. Model fit was summarized using the log-likelihood, model chi-square test, and McFadden pseudo *R*^2^. McFadden pseudo *R*^2^ was used only as an index of relative explanatory fit and was not interpreted as the proportion of variance explained in the same way as *R*^2^ in linear regression. A 2-tailed *P* value of <.05 was considered statistically significant.

To assess potential nonresponse bias, we conducted a wave analysis comparing early and late respondents among valid questionnaires after the prespecified quality control screening. Because individual-level information from eligible patients who declined or did not complete the survey was unavailable, late respondents were used as a proxy for potential nonrespondents. Valid responses were ordered chronologically according to questionnaire submission time. The first 3 quartiles were classified as early respondents, and the final quartile was classified as late respondents. Demographic characteristics, clinical characteristics, questionnaire completion time, rehabilitation stage, and stated willingness to use AI-enabled functions were compared between early and late respondents using independent-samples *t* tests or Mann-Whitney *U* tests for continuous variables and chi-square or Fisher exact tests for categorical variables, as appropriate. This analysis was exploratory and was intended to assess potential nonresponse bias rather than to modify the primary regression model.

## Results

### Participant Characteristics and Data Quality Control

**Figure 1.**
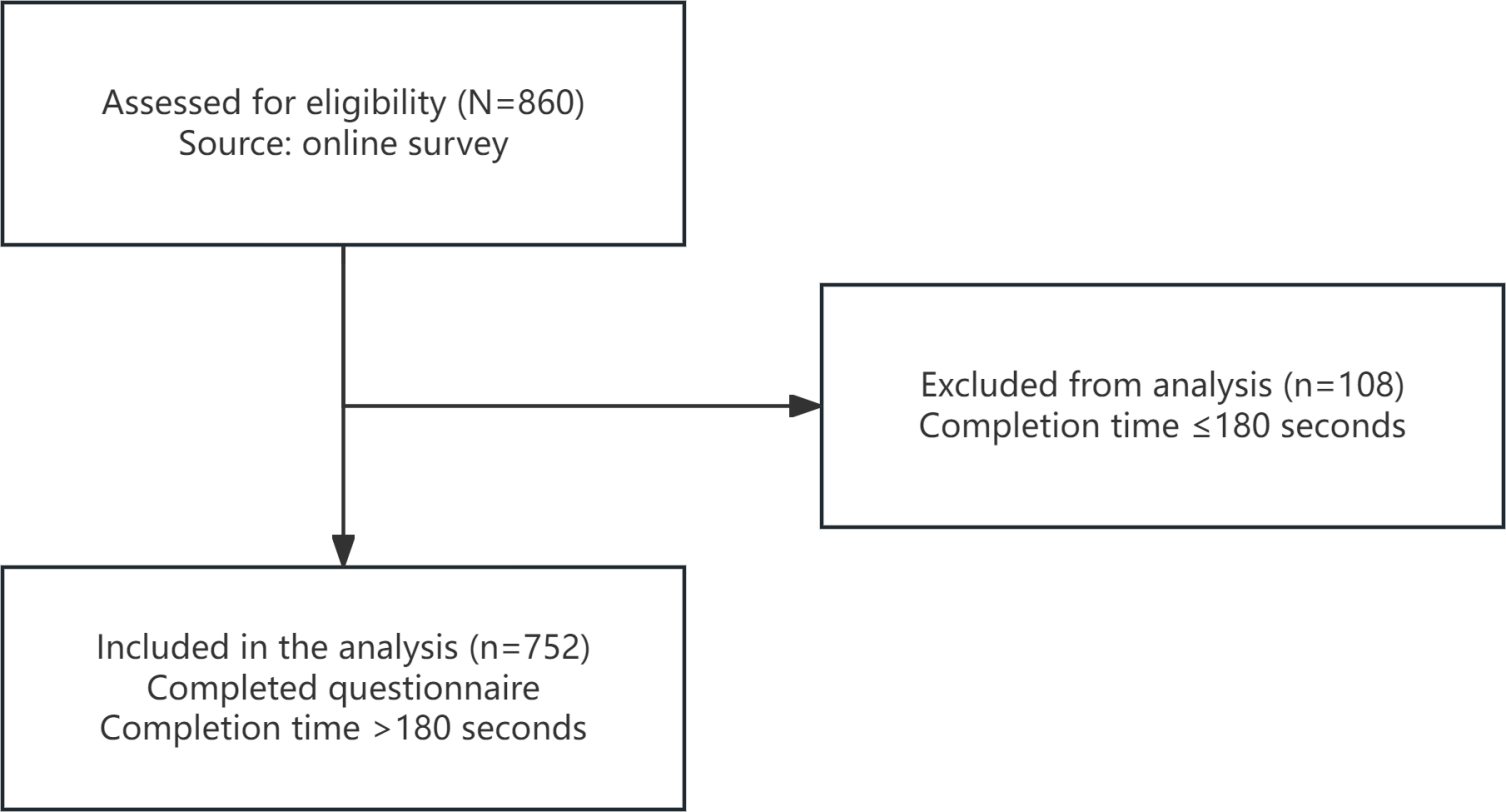
Recruitment and data screening process flow diagram.

The recruitment and data screening process is illustrated in Figure 1. A total of 860 questionnaires were submitted. To ensure data integrity, 108 responses with a completion time of 180 seconds or less were excluded as invalid. The final analysis included 752 valid participants (effective response rate is 752/860, 87.4%).

The demographic and clinical characteristics of the participants are detailed in Table 1. The mean age was 45.72 (SD 17.98) years. Of 752 participants, 387 (51.5%) were female participants. Most participants resided in nonrural urban areas (n=627, 83.4%) and were recruited from tertiary hospitals (n=710, 94.4%). Overall, stated willingness to use AI-supported functions for orthopedic transitional care was high, with 604 (80.3%) classified as willing. In unadjusted comparisons, participants who were willing to use AI were older than those who were not willing (mean 46.54, SD 17.91 y vs mean 42.37, SD 17.95 y; *P*=.01) and were more likely to reside in nonrural urban areas (514/604, 85.1% vs 113/148, 76.4%; *P*=.02). Detailed group comparisons are presented in Table 1.

**Table 1.** Demographic and clinical characteristics by stated willingness to use AI-supported functions (N=752).

Characteristic	Total (N=752)	Willing group (n=604)	Not willing group (n=148)	*P* value
Age (y), mean (SD)	45.72 (17.98)	46.54 (17.91)	42.37 (17.95)	.01
Sex, n (%)				.63
Female	387 (51.5)	314 (52.0)	73 (49.3)	
Male	365 (48.5)	290 (48.0)	75 (50.7)	
Education level, n (%)				.56
Primary or below	130 (17.3)	108 (17.9)	22 (14.9)	
Secondary school	345 (45.9)	272 (45.0)	73 (49.3)	
University or above	277 (36.8)	224 (37.1)	53 (35.8)	
Marital status, n (%)				.11
Married	526 (69.9)	431 (71.4)	95 (64.2)	
Unmarried or divorced or widowed	226 (30.1)	173 (28.6)	53 (35.8)	
Residence, n (%)				.02
Rural	125 (16.6)	90 (14.9)	35 (23.6)	
Nonrural (urban)	627 (83.4)	514 (85.1)	113 (76.4)	
Living status, n (%)				.10
Living alone	117 (15.6)	87 (14.4)	30 (20.3)	
Living with others	635 (84.4)	517 (85.6)	118 (79.7)	
Employment status, n (%)				.73
Employed	364 (48.4)	290 (48.0)	74 (50)	
Unemployed or retired	388 (51.6)	314 (52.0)	74 (50)	
Hospital level, n (%)				.62
Tertiary	710 (94.4)	572 (94.7)	138 (93.2)	
Secondary	42 (5.6)	32 (5.3)	10 (6.8)	
Medical diagnosis, n (%)				.08
Nontumor	577 (76.7)	472 (78.1)	105 (70.9)	
Tumor	175 (23.3)	132 (21.9)	43 (29.1)	
Comorbidities, n (%)				.20
No	195 (25.9)	150 (24.8)	45 (30.4)	
Yes	557 (74.1)	454 (75.2)	103 (69.6)	
Physical disability, n (%)				.86
No	389 (51.7)	311 (51.5)	78 (52.7)	
Yes	363 (48.3)	293 (48.5)	70 (47.3)	
Self-care ability, n (%)				.24
Fully independent	155 (20.6)	126 (20.8)	29 (19.6)	
Mostly independent	181 (24.1)	146 (24.2)	35 (23.6)	
Partially dependent	334 (44.4)	273 (45.2)	61 (41.2)	
Dependent	82 (10.9)	59 (9.8)	23 (15.6)	
Rehabilitation stage, n (%)				.25
Preoperative	136 (18.1)	106 (17.5)	30 (20.3)	
Postoperative (inpatient)	488 (64.9)	386 (63.9)	102 (68.9)	
Home care (early stage)	76 (10.1)	66 (10.9)	10 (6.8)	
Home care (mid stage)	14 (1.9)	13 (2.2)	1 (0.6)	
Home care (late stage)	38 (5.0)	33 (5.5)	5 (3.4)	
Psychometric factors, mean (SD)				
Perceived home rehab barriers	0.00 (0.96)	0.03 (0.98)	−0.12 (0.85)	.10
Lack of professional support	0.00 (0.92)	−0.03 (0.95)	0.13 (0.77)	.05
Symptom uncertainty	0.00 (0.88)	0.02 (0.90)	−0.07 (0.82)	.30

### Nonresponse Bias Assessment

To assess potential nonresponse bias, we conducted a wave analysis comparing early and late respondents after applying the prespecified quality control criterion. Among the 752 valid respondents, 564 were classified as early respondents and 188 as late respondents according to questionnaire submission time. The proportion of respondents reporting willingness to use AI systems was identical between early and late respondents (453/564, 80.3% vs 151/188, 80.3%; *P*>.99). Age, questionnaire completion time, sex, education, marital status, residence, living status, employment status, comorbidities, physical disability, self-care ability, and rehabilitation stage were not significantly different between groups. Hospital level and medical diagnosis differed significantly between early and late respondents. Overall, the early vs late comparison did not support the concern that the high willingness estimate was driven by more enthusiastic early respondents. However, because late respondents are only a proxy for true nonrespondents, nonresponse bias cannot be fully excluded. Detailed results are provided in Table 2.

**Table 2.** Comparison of early and late respondents for assessment of potential nonresponse bias (N=752).

Variable	Early respondents (n=564)	Late respondents (n=188)	*P* value
Age (y), mean (SD)	45.75 (17.91)	45.64 (18.24)	.95
Questionnaire completion time (s), median (IQR)	447 (296.75‐734.00)	452 (295.0‐800.5)	.81
Willingness to use AI systems, n (%)	453 (80.3)	151 (80.3)	>.99
Sex, n (%)			.16
Female	282 (50)	105 (55.9)	
Male	282 (50)	83 (44.1)	
Education level, n (%)			.16
Primary or below	95 (16.8)	35 (18.6)	
Secondary school	270 (47.9)	75 (39.9)	
University or above	199 (35.3)	78 (41.5)	
Marital status, n (%)			.65
Married	397 (70.4)	129 (68.6)	
Unmarried/divorced/widowed	167 (29.6)	59 (31.4)	
Residence, n (%)			.53
Rural	91 (16.1)	34 (18.1)	
Nonrural or urban	473 (83.9)	154 (81.9)	
Living status, n (%)			.38
Living alone	84 (14.9)	33 (17.6)	
Living with others	480 (85.1)	155 (82.4)	
Employment status, n (%)			.18
Employed	281 (49.8)	83 (44.1)	
Unemployed/Retired	283 (50.2)	105 (55.9)	
Hospital level, n (%)			<.001
Tertiary	522 (92.6)	188 (100)	
Secondary	42 (7.4)	0 (0)	
Medical diagnosis, n (%)			.04
Nontumor	443 (78.5)	134 (71.3)	
Tumor	121 (21.5)	54 (28.7)	
Comorbidities, n (%)			.74
No	148 (26.2)	47 (25)	
Yes	416 (73.8)	141 (75)	
Physical disability, n (%)			.77
No	290 (51.4)	99 (52.7)	
Yes	274 (48.6)	89 (47.3)	
Self-care ability, n (%)			.43
Fully independent	123 (21.8)	32 (17.0)	
Mostly independent	138 (24.5)	43 (22.9)	
Partially dependent	243 (43.1)	91 (48.4)	
Dependent	60 (10.6)	22 (11.7)	
Rehabilitation stage, n (%)			.09
Preoperative	97 (17.2)	39 (20.7)	
Postoperative or inpatient	360 (63.8)	128 (68.1)	
Home care, early stage	66 (11.7)	10 (5.3)	
Home care, mid stage	12 (2.1)	2 (1.1)	
Home care, late stage	29 (5.1)	9 (4.8)	

### Psychometric Structure of Perceived Transitional-Care Challenges

To examine the structure of the perceived transitional-care challenge items, EFA was conducted on the 17-item Perceived Challenges Scale. The scale showed excellent sampling adequacy (Kaiser-Meyer-Olkin measure is 0.96), and the Bartlett test of sphericity was statistically significant (*P*<.001), supporting the suitability of the data for factor analysis. Three factors were extracted, explaining 72.1% of the total variance. These factors were interpreted as contextual transitional-care challenge dimensions rather than as core TAM constructs.

Factor 1 was labeled “Home rehabilitation self-management barriers” and explained 32.8% of the variance (Cronbach α=0.96). This factor reflected difficulties in home rehabilitation execution, motivation, understanding, and feedback. Factor 2 was labeled “Lack of professional support” and explained 22.2% of the variance (Cronbach α=0.92). This factor reflected fragmented care, inconsistent education, and delayed professional feedback. Factor 3 was labeled “Symptom uncertainty” and explained 17.1% of the variance (Cronbach α=0.86). This factor reflected uncertainty related to pain, complications, and postoperative symptoms. The rotated factor loadings are presented in Table 3.

**Table 3.** Rotated factor loadings for the Perceived Challenges Scale (N=752).

Item	F1: Self-management barriers	F2: Lack of professional support	F3: Symptom uncertainty
Factor 1 items (α=0.96)			
Fear of overexercise or underexercise and lack of feedback	0.825	0.203	0.331
Lack of motivation and poor understanding	0.813	0.241	0.315
Uncertainty about sudden symptoms (swelling or fever)	0.794	0.234	0.324
Caregiver lacks professional nursing knowledge	0.773	0.340	0.209
Anxiety due to slow rehabilitation progress	0.763	0.379	0.170
Unsure about exercise correctness or lack of guidance	0.750	0.186	0.400
Trouble traveling for follow-up visits	0.714	0.366	0.154
Forgetting medication or exercise schedules	0.648	0.500	0.134
Factor 2 items (α=0.96)			
Inconsistent education from different staff	0.261	0.818	0.185
Busy staff and delayed professional feedback	0.302	0.747	0.293
Difficulty understanding medical terminology	0.252	0.616	0.485
Inability to self-care during bed rest	0.369	0.587	0.346
Disconnected transition from hospital to home	0.458	0.569	0.416
Anxiety about complications (DVT^a^ or infection)	0.358	0.559	0.440
Factor 3 items (α=0.86)			
Lack of personalized rehabilitation plan	0.235	0.422	0.719
Fear of nonstandard movements affecting recovery	0.279	0.172	0.713
Uncertainty about pain severity (normal vs abnormal)	0.324	0.391	0.668
Reliability (Cronbach α)	0.960	0.920	0.860
Eigenvalues	10.500	1.590	1.010
Variance explained (%)	32.810	22.230	17.060

a DVT: deep vein thrombosis.

### Mapping Stated AI Function Preferences Across the Care Continuum

**Figure 2.**
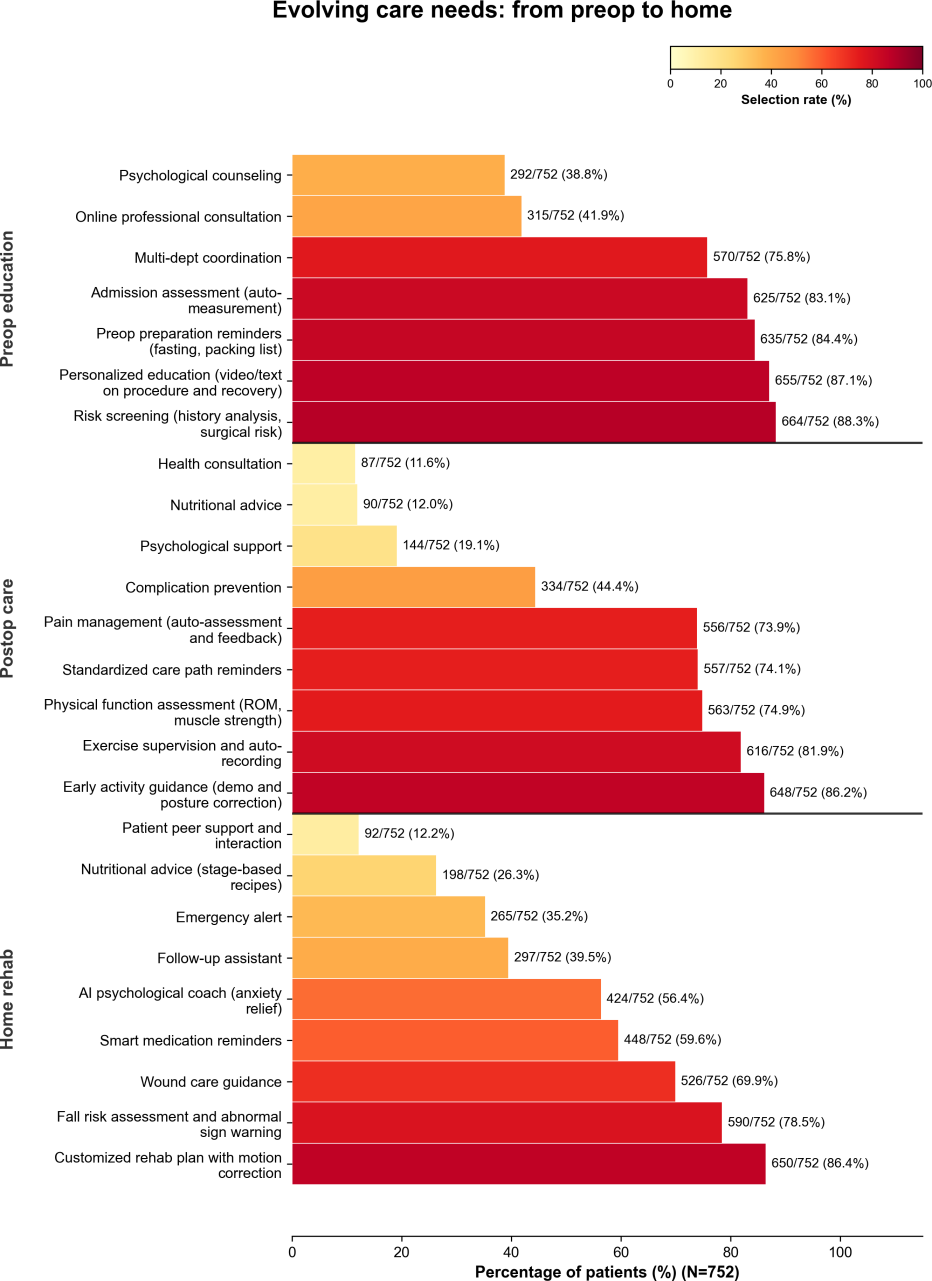
Heatmap evolving AI function preferences across care phases. Bars show the proportion of participants (N=752) selecting each AI function as necessary. Color intensity represents the same proportion and does not indicate a separate construct. ROM: range of motion.

Patient-reported care priorities and preferences for AI-supported functions differed across the hospital-to-home care continuum. As shown in Figure 2, inpatient-stage preferences were more frequently related to information acquisition, education, risk screening, and care instructions, whereas home-stage preferences were more frequently related to rehabilitation guidance, motion correction, fall prevention, symptom monitoring, and risk alerts. Customized rehabilitation plans and motion correction remained highly preferred during the postoperative and home recovery phases.

**Figure 3.**
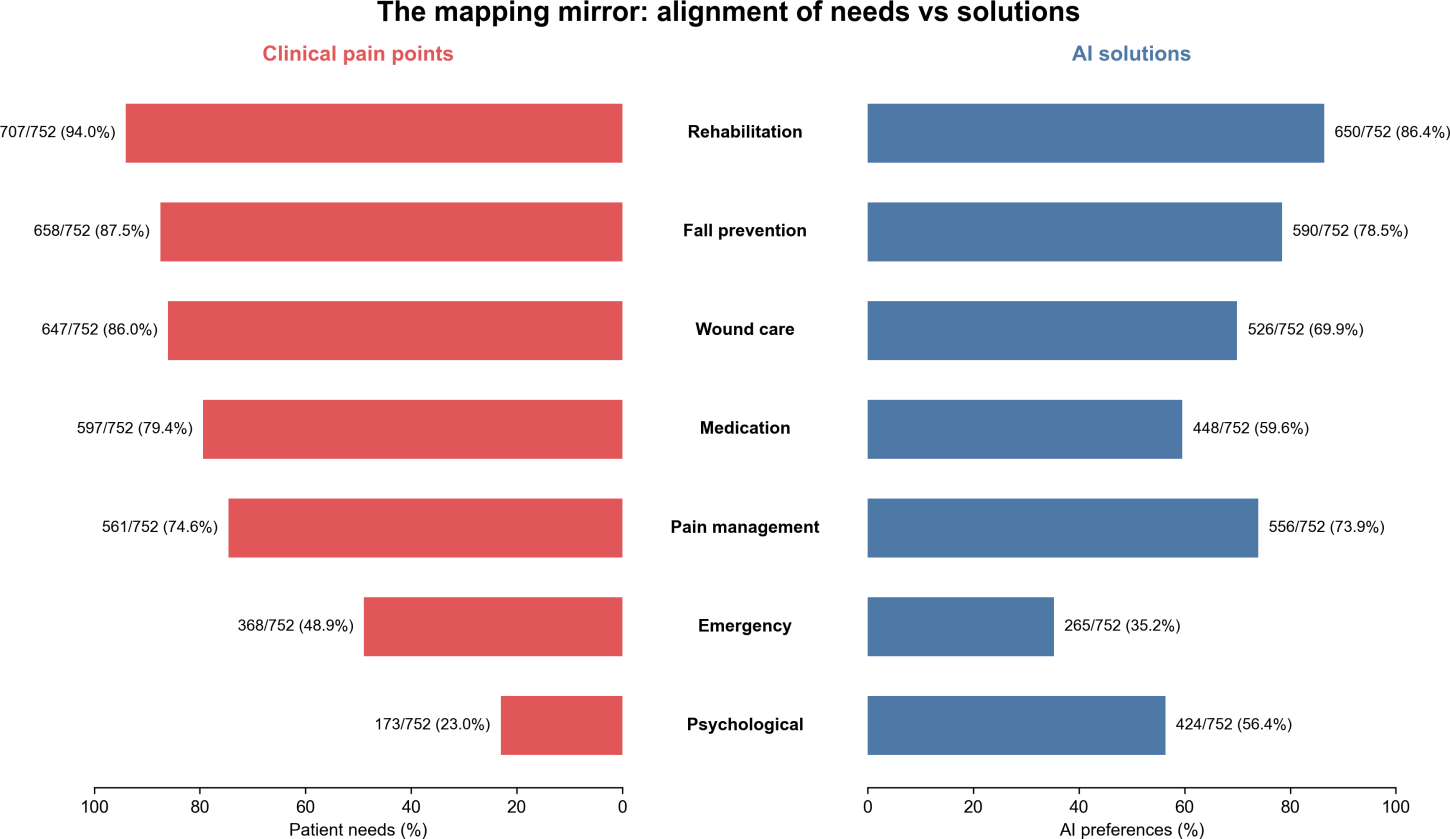
Descriptive mapping of patient-reported care needs and AI function preferences (N=752).

To descriptively examine the correspondence between patient-reported care needs and preferences for AI-supported functions, we mapped these 2 sets of responses in Figure 3. A descriptive concordance was observed for rehabilitation-related support and fall prevention, where high patient-reported care needs corresponded to a high preference for AI-supported functions. Rehabilitation-related support showed high levels of both reported need and AI-supported function preference (707/752, 94.0% vs 650/752, 86.4%), as did fall prevention (658/752, 87.5% vs 590/752, 78.5%). This pattern indicates alignment between reported physical care needs and stated AI function preferences, but it should not be interpreted as evidence that these functions would be effective in actual use.

A discrepancy was observed in the psychological domain. Although fewer participants explicitly ranked psychological support as a top care need (173/752, 23.0%), more participants expressed interest in AI-supported psychological coaching (424/752, 56.4%). This pattern may indicate openness to AI-supported emotional functions, although whether such functions would be acceptable, safe, or effective in actual use requires further evaluation.

Figure 3 displays the descriptive correspondence between reported clinical care needs and preferences for AI-supported functions. Categories are sorted by the magnitude of patient-reported care needs.

### Factors Associated With Willingness to Use AI-Supported Functions

**Figure 4.**
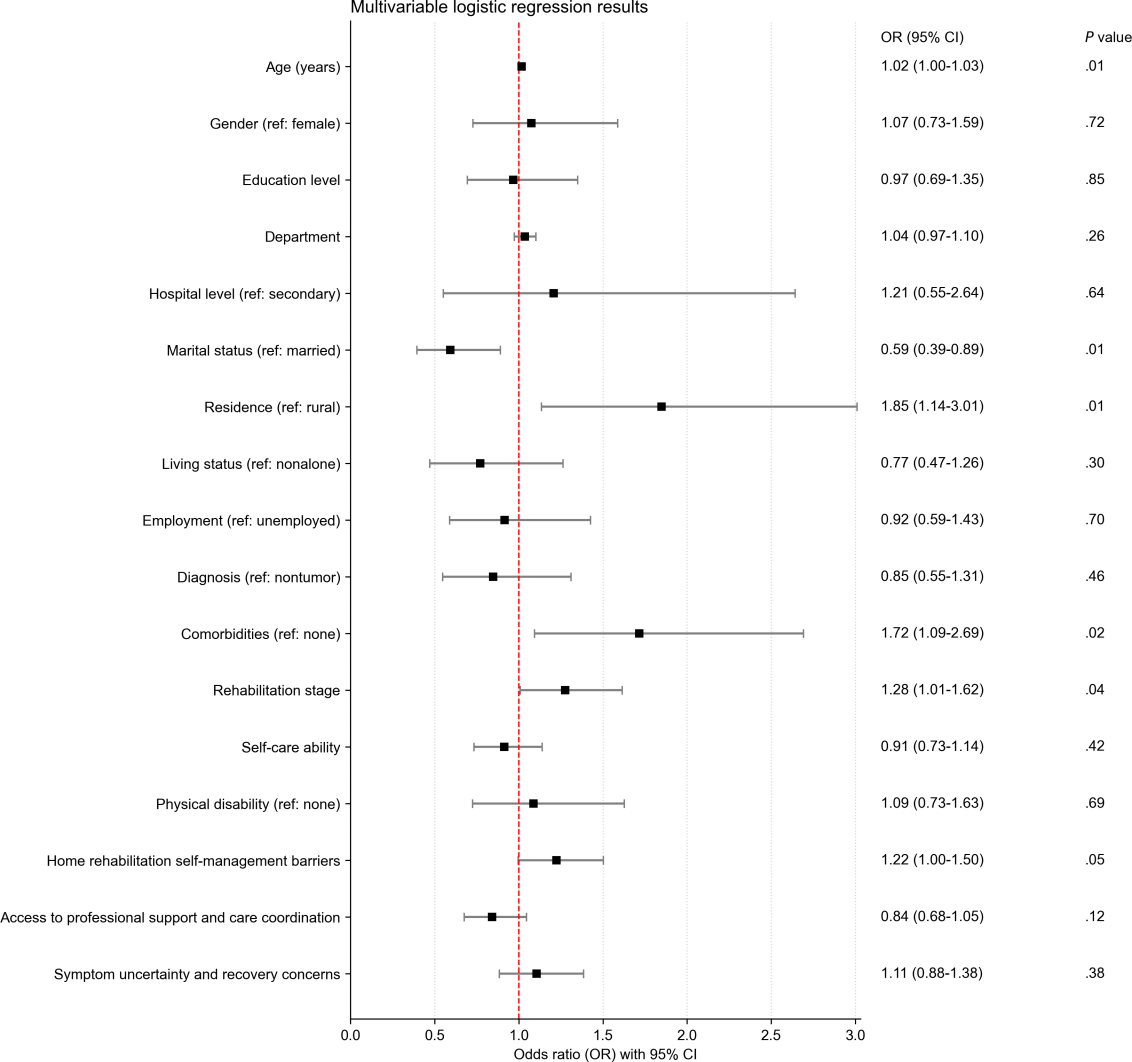
Forest plot of adjusted odds ratios for factors associated with the willingness to use AI-supported functions (N=752). “Residence” refers to nonrural (urban) vs rural; “marital status” refers to married vs unmarried or divorced or widowed; “comorbidities” refers to presence vs absence.

Exploratory multivariable logistic regression identified several factors associated with the stated willingness to use AI-supported functions. The results suggest that selected clinical and sociodemographic characteristics were associated with willingness, whereas not all perceived challenge or functional status variables were independently significant after adjustment. Although the overall model was statistically significant, its explanatory fit was modest (McFadden pseudo *R*^2^=0.056). Therefore, the aORs should be interpreted as exploratory associations rather than as a prediction rule or evidence of causal mechanisms. The results are visualized in Figure 4 and detailed in Table 4.

Older age, the presence of comorbidities, and a later rehabilitation stage were associated with a higher willingness to use AI-supported functions. Participants with comorbidities had higher odds of reporting willingness to use AI-supported functions than those without comorbidities (adjusted odds ratio [aOR] 1.72, 95% CI 1.09‐2.69; *P*=.02). Older age was also associated with higher willingness (aOR 1.02, 95% CI 1.00‐1.03; *P*=.01), as was a later rehabilitation stage (aOR 1.28, 95% CI 1.01‐1.62; *P*=.04). These findings may be consistent with the possibility that patients facing more complex recovery contexts perceive greater potential value in AI-supported assistance, but this interpretation remains hypothetical because of the cross-sectional design.

Sociodemographic characteristics were also associated with stated willingness. Participants residing in nonrural urban areas had higher odds of reporting willingness than rural participants (aOR 1.85, 95% CI 1.14‐3.01; *P*=.01). Marital status was associated with willingness; unmarried, divorced, or widowed participants had lower odds of reporting willingness than married participants (aOR 0.59, 95% CI 0.39‐0.89; *P*=.01). These associations may reflect broader access-related or social context factors; however, digital literacy, internet access, caregiver involvement, family assistance with technology use, and clinician encouragement were not directly measured in this study.

In contrast, physical disability (aOR 1.09, 95% CI 0.73‐1.63; *P*=.69) and self-care ability (aOR 0.91, 95% CI 0.73‐1.14; *P*=.42) were not independently associated with willingness in the adjusted model. Among the perceived challenge factors, factor 1 (F1: Home rehabilitation self-management barriers) was not statistically significant at the prespecified .05 threshold, although the point estimate was positive (aOR 1.22, 95% CI 1.00‐1.50; *P*=.05). Factor 2 (F2: Lack of professional support; aOR 0.84, 95% CI 0.68‐1.05; *P*=.12) and factor 3 (F3: Symptom uncertainty; aOR 1.11, 95% CI 0.88‐1.38; *P*=.38) were also not statistically significant after adjustment.

**Table 4.** Exploratory multivariable logistic regression analysis of factors associated with willingness to use AI-supported functions (N=752).^a^

Variable	β (SE)	aOR (95% CI)	*P* value
Sociodemographic characteristics			
Age (y)	0.02 (0.01)	1.02 (1.00-1.03)	.01
Sex (male vs female)	0.07 (0.20)	1.08 (0.73-1.59)	.72
Education level	−0.03 (0.17)	0.97 (0.69-1.35)	.85
Marital status (unmarried vs married)	−0.52 (0.21)	0.59 (0.39-0.89)	.01
Residence (urban vs rural)	0.61 (0.25)	1.85 (1.14-3.01)	.01
Living status (alone vs with others)	−0.26 (0.25)	0.77 (0.47-1.26)	.30
Employment (unemployed vs employed)	−0.09 (0.23)	0.92 (0.59-1.43)	.70
Clinical characteristics			
Hospital level (tertiary vs secondary)	0.19 (0.40)	1.21 (0.55-2.64)	.64
Department category	0.04 (0.03)	1.04 (0.97-1.10)	.26
Diagnosis (tumor vs nontumor)	−0.17 (0.22)	0.85 (0.55‐1.31)	.46
Comorbidities (yes vs no)	0.54 (0.23)	1.72 (1.09‐2.69)	.02
Rehabilitation stage (later stage)	0.24 (0.12)	1.28 (1.01‐1.62)	.04
Self-care ability (worse status)	−0.09 (0.11)	0.91 (0.73‐1.14)	.42
Physical disability (yes vs no)	0.08 (0.21)	1.09 (0.73‐1.63)	.69
Psychometric factors (perceived challenges)			
F1: Home rehab self-management barriers	0.20 (0.11)	1.22 (1.00‐1.50)	.05
F2: Lack of professional support	−0.17 (0.11)	0.84 (0.68‐1.05)	.12
F3: Symptom uncertainty	0.10 (0.11)	1.11 (0.88‐1.38)	.38
Constant	−0.52 (1.01)	—^b^	.61

a Model fit statistics: log-likelihood=−352.20; *χ*2 test: *P*<.001; pseudo *R*2 (McFadden)=0.056.

b Not applicable.

### Barriers to Adoption and Perceived Risks

Despite the high stated willingness to use AI-supported functions, participants reported several barriers that might hinder future adoption. Usability and trust-related concerns were among the most frequently reported barriers. The most common usability concern was that operation would be too complicated (566/752, 75.3%), followed by concerns about excessive interface information or information overload (472/752, 62.7%).

Trust and security concerns were also common. A total of 517 participants (n=752, 68.8%) reported concern about excessive advertising or unreliable content, and 447 (n=752, 59.5%) reported concerns about personal information security. These concerns were reported more frequently than fear of unreasonable charges (348/752, 46.3%). These findings suggest that future implementation studies and AI prototype development should address usability, information reliability, privacy protection, and clinician oversight, rather than assuming that willingness to use AI-supported functions will translate directly into real-world adoption.

## Discussion

### Principal Findings

This multicenter cross-sectional survey mapped patient-reported priorities and stated willingness to use AI in orthopedic transitional care. Overall, stated willingness was high among valid respondents (604/752, 80.3%). Patient-reported priorities differed across care phases: inpatient-stage priorities were more commonly related to information acquisition and care instruction, whereas home-stage priorities were more commonly related to functional safety, rehabilitation guidance, motion correction, fall prevention, symptom monitoring, and risk alerts. In exploratory adjusted analysis, older age, the presence of comorbidities, later rehabilitation stage, nonrural urban residence, and marital status were associated with stated willingness to use AI. Physical disability, self-care ability, and most perceived challenge factor scores were not statistically significant after adjustment.

These findings should be interpreted within the limitations of the study design and measurement approach. Because this was a cross-sectional survey, the observed associations do not establish causal pathways or temporal ordering. In addition, participants evaluated standardized function-based AI descriptions rather than actual AI tools, interfaces, wearable devices, or monitoring platforms. Therefore, the results should be interpreted as stated preferences and exploratory associations rather than evidence of actual adoption, clinical effectiveness, or causal mechanisms.

### Comparison With Prior Work

The observed pattern suggests that stated priorities may differ across the orthopedic transitional-care continuum. During hospitalization, participants more often prioritized information acquisition, understanding of care instructions, risk screening, and preparation for surgery or discharge. This pattern is consistent with previous work showing that LLM chatbots can support patient education and question answering in orthopedic contexts [9,10,15]. After discharge, however, priorities shifted toward task execution and safety, including rehabilitation guidance, motion correction, fall prevention, symptom monitoring, and timely risk alerts [22,23].

This descriptive pattern supports the relevance of moving beyond generic information delivery when designing future AI-supported transitional-care tools. However, the findings should not be interpreted as evidence that these AI-supported functions improve recovery outcomes or reduce adverse events. Instead, they identify patient-prioritized candidate functions that may warrant future prototype development, usability testing, and real-world evaluation.

Older age, comorbidities, and later rehabilitation stages were associated with greater stated willingness to use AI. One possible interpretation is that patients facing more complex recovery contexts may perceive greater potential value in digital support. However, the cross-sectional design cannot determine whether clinical complexity increases willingness, whether willingness influences how patients report their needs, or whether unmeasured factors account for these associations. This caution is important because physical disability and self-care ability were not independently associated with willingness in the adjusted model. Therefore, willingness to use AI should not be interpreted as being driven simply by objective functional impairment. Instead, it may reflect a broader combination of perceived need, anticipated benefit, social support, technology access, clinician encouragement, trust, and previous digital health experience.

This study should be interpreted as TAM-informed rather than as a full empirical test or extension of the TAM [21]. Perceived usefulness, perceived ease of use, and attitude toward use were not directly measured. Therefore, the EFA-derived challenge dimensions should be viewed as contextual transitional-care challenges rather than core TAM constructs or validated TAM pathways. After adjustment, factor 1 (F1: Home rehabilitation self-management barriers) did not meet the prespecified threshold for statistical significance, although the point estimate was positive (aOR 1.22, 95% CI 1.00‐1.50; *P*=.05). Factor 2 (F2: Lack of professional support) and factor 3 (F3: Symptom uncertainty) were also not statistically significant. Accordingly, the findings do not demonstrate that perceived challenge dimensions independently drive willingness to use AI. Future TAM-based or implementation-focused studies should directly measure perceived usefulness, perceived ease of use, trust, privacy concerns, digital literacy, prior digital health experience, clinician recommendation, and actual use behavior.

The adjusted model identified several statistically significant associations, but its explanatory fit was modest, with a McFadden pseudo *R*^2^ of 0.056. This indicates that a substantial proportion of variability in stated willingness remained unexplained. The regression results should, therefore, be interpreted as exploratory signals rather than as a prediction rule or a comprehensive explanatory model of AI acceptance. The associations with residence and marital status also require cautious interpretation. Nonrural urban residence may reflect broader access to specialist care, digital services, and health technology exposure. Marital status may reflect differences in family support or care resources. However, the study did not directly measure digital literacy, internet access, caregiver involvement, family assistance with technology use, or clinician encouragement. These variables may partly explain the observed associations and should be incorporated into future models.

These implementation concerns also underscore the importance of trust, information reliability, and human oversight. This is consistent with recent work showing that physiotherapy students recognize practical uses of AI chatbots but remain cautious about their role in clinical reasoning because of misinformation risks, and with nursing literature emphasizing that AI should support rather than replace human-centered care [24,25]. Accordingly, future AI-supported transitional-care tools should include clear pathways for clinician review, particularly when patients report complex symptoms, uncertainty, or potential complications.

### Limitations

Several limitations should be considered when interpreting these findings. The most important is the cross-sectional design. Patient characteristics, perceived transitional-care challenges, and willingness to use AI were measured at the same time, so temporal ordering could not be established. Therefore, observed associations should not be interpreted as evidence that age, comorbidities, rehabilitation stage, residence, or marital status caused higher willingness to use AI. Reverse causality and residual confounding remain possible, particularly because factors such as digital literacy, previous experience with digital health tools, family caregiving resources, clinician encouragement, and trust in AI-supported care were not directly measured.

The sampling strategy also limits generalizability. Participants were recruited using convenience sampling, and the sample was concentrated in tertiary hospitals and nonrural settings. Patients in these settings may have more exposure to specialist care, digital health services, and advanced medical technologies than patients in rural, primary care, or lower-resource settings. As a result, the observed willingness rate should be interpreted as applying to this hospital-based respondent sample rather than as a population-level estimate for all patients recovering from orthopedic surgery.

We attempted to assess potential nonresponse bias by comparing early and late respondents, using late respondents as a proxy for potential nonrespondents. The proportion reporting willingness to use AI was identical between early and late respondents (453/564, 80.3% vs 151/188, 80.3%; *P*>.99), which does not support the concern that the overall willingness estimate was driven mainly by more enthusiastic early respondents. However, this analysis cannot replace direct information from patients who declined or did not complete the survey. Late respondents may differ from true nonrespondents in ways that were not captured in the available data.

Another limitation concerns measurement. Participants evaluated standardized function-based AI descriptions rather than actual AI tools, interfaces, wearable devices, or monitoring platforms. Their responses may, therefore, reflect anticipated usefulness under simplified survey conditions. Real-world use could be lower when patients encounter setup requirements, interface complexity, false alerts, privacy authorization, costs, alarm fatigue, or uncertainty about clinician oversight. In addition, researcher-assisted completion was used for participants with limited digital literacy or reading difficulties. Although this approach improved inclusion, it may have introduced social desirability or interviewer bias, and the administration mode was not retained as a separate analyzable variable.

Finally, the adjusted model had limited explanatory fit, and several constructs relevant to technology acceptance were not directly measured. Although the study was conceptually informed by the TAM, perceived usefulness, perceived ease of use, and attitude toward use were not assessed with validated multi-item measures. Therefore, the regression findings should be viewed as exploratory associations rather than as a prediction model or a comprehensive explanation of willingness to use AI. Future longitudinal, implementation, and prototype-based studies are needed to determine whether stated willingness translates into actual uptake, sustained engagement, and improved recovery outcomes.

### Conclusions

This study identified a high stated willingness to use AI and a descriptive shift in patient-reported priorities from information support during hospitalization to functional safety and rehabilitation support after discharge. Older age, comorbidities, later rehabilitation stage, nonrural or urban residence, and marital status were associated with stated willingness, but these findings should be interpreted as exploratory associations rather than causal determinants. Because participants evaluated function-based AI descriptions rather than actual AI tools, the findings should be used to inform candidate areas for future prototype development and real-world evaluation. Future orthopedic AI research should assess usability, safety, privacy, clinician oversight, sustained engagement, and clinical outcomes across diverse care settings, including rural, primary care, and lower-resource environments.

## Supplementary material

10.2196/91061 Checklist 1 CHERRIES checklist.

10.2196/91061 Checklist 2 STROBE-Equity guidelines.

10.2196/91061 Checklist 3 CROSS checklist.

## Abbreviations

aOR: adjusted odds ratio
CHERRIES: Checklist for Reporting Results of Internet E-Surveys
CROSS: Consensus-Based Checklist for Reporting of Survey Studies
EFA: exploratory factor analysis
EPV: events per variable
LLM: large language model
OR: odds ratio
STROBE-Equity: Strengthening the Reporting of Observational Studies in Epidemiology—Equity
TAM: technology acceptance model
