# Supplementary material for: Mapping the Evolving AI Preferences and Care Needs in Orthopedic Transitional Care From Hospitals to Home: Cross-Sectional Study
Source: JMIR AI. 2026 Aug 4;5:e91061. doi: 10.2196/91061 (PMC13436801; doi:10.2196/91061)
Supplement: Supplementary file 2 [file ai-v5-e91061-s002.pdf]

JAMA Network Open

Special Communication | Global Health

Improving the Reporting on Health Equity in Observational Research (STROBE-Equity)
Extension Checklist and Elaboration

Omar Dewidar, MSc; Larissa Shamseer, PhD; G. J. Melendez-Torres, PhD; Elie A. Akl, PhD; Jacqueline Ramke, PhD; Xiaoqin Wang, PhD; Oyekola Oloyede, PhD; Taryn Young, PhD; Stuart G. Nicholls, PhD; Zack Marshall, PhD; Michelle Kennedy, PhD; Billie-Jo Hardy, PhD; Anita Rizvi, PhD; Elizabeth Ghogomu, MD; Tamara Rader, MLIS; Hugh Sharma Waddington, PhD; Beverley Shea, PhD; Miriam Nkangu, PhD; Holly Ellingwood, PhD; Luke Wolfenden, PhD; Janice Tufte; Tanya Horsley, PhD; Kevin Pottie, MD; Luis Gabriel Cuervo, MD; Clara Juando-Prats, PhD; Cindy Feng, PhD; Melissa K. Sharp, PhD; Julian Little, PhD; Ebenezer Owusu-Addo, PhD; Damian Francis, PhD; Tamara Kredo, PhD; Michael Johnson Mahande, PhD; Catherine Chamberlain, PhD; Tomás Pantoja, MD; Erik von Elm, PhD; Zulfiqar A. Bhutta, PhD; Peter Tugwell, MD; Charles S. Wiysonge, PhD; Sarah Funnell, MD; Janet Jull, PhD; Lawrence Mbuagbaw, PhD; Vivian Welch, PhD

Abstract

IMPORTANCE Observational studies can provide valuable insights to inform decisions on health equity. Existing guidelines for reporting such studies, such as the Strengthening the Reporting of Observational Studies in Epidemiology (STROBE) statement, currently lack specific considerations for reporting on health equity. Health equity is defined as the absence of avoidable and unfair differences that may exist across individuals and populations due to structural and systematic inequities in living and working conditions, opportunities, and resources. To address this gap, the research team developed an extension of the STROBE statement (STROBE-Equity) that focuses on reporting health equity data and considerations.

OBSERVATIONS This consensus statement followed steps for developing a consensus- and evidence-based guideline using an integrated knowledge translation approach to ensure engagement of knowledge users from diverse disciplines and perspectives. Selection criteria for the research team and steering committees prioritized diversity across age, gender, and geography. The STROBE checklist was extended to include 10 items specifically aimed at reporting health equity considerations. To develop these items, the research team drew on evidence from empirical studies including a scoping review of the literature, methodological review, key informant interviews, an online survey, and a global consensus meeting of experts. For each of the 10 equity-related items, the statement provides an explanation and example(s) of transparent reporting practices.

CONCLUSIONS AND RELEVANCE Use of the STROBE-Equity extension alongside the STROBE statement when writing up completed reports of observational studies has the potential to advance the reporting of health equity data and considerations. Improved reporting of this information may help knowledge users better identify and apply evidence relevant to populations experiencing inequities.

JAMA Network Open. 2025;8(9):e2532512. doi:10.1001/jamanetworkopen.2025.32512

+ Supplemental content

Author affiliations and article information are listed at the end of this article.

Introduction

Health equity indicators are frequently collected in observational studies, making them an important source of information for population health (**Box**). The Strengthening the Reporting of Observational Studies in Epidemiology (STROBE)[17] statement is considered the current criterion standard for reporting observational studies. However, the consideration of health equity is outside the scope of the STROBE statement.[18] Health equity is achieved when everyone has an equal and fair opportunity

Open Access. This is an open access article distributed under the terms of the CC-BY License.

Downloaded from jamanetwork.com by guest on 07/17/2026

to attain optimal health.[19] Evidence consistently demonstrates that certain groups consistently experience barriers to achieving health equity arising from unequal access to resources and opportunities.[20,21] These barriers are exacerbated by structural inequities that manifest as uneven power and privilege distribution, disadvantaging certain groups based on their living and working conditions, personal and social characteristics (eg, gender, age, ethnicity, occupation).[3,22-24] International agreements have called for the development of standards for health equity-relevant research to promote health equity.[25] Despite this, data suggests that inequitable experiences are often poorly reported in observational studies, leaving many population groups underrepresented in

Box. Definitions

Observational study: A study that does not involve any intervention (experimental or otherwise) on the part of the investigator.[1]

Health equity–relevant observational studies: Studies that investigate outcomes related to populations experiencing or that may experience inequities and/or examine variations in outcomes among groups experiencing different levels of inequities or no inequities at all. In some cases, these observational studies may meet both criteria by focusing on populations experiencing inequities and assessing subgroup differences. Importantly, this definition does not require the studies to explicitly state that the objective of the observational study is linked to health equity.[2] It is intended for those that address unfair or unjust differences and may not apply to differences that are unavoidable (eg, biological). For example, a study reporting differences in breast cancer incidence between age groups may reflect biological variation, as age is a nonmodifiable risk factor. In contrast, a study reporting disparities in breast cancer mortality between ethnic groups due to delays in diagnosis or unequal access to treatment would qualify as health equity–relevant.

Characteristics associated with inequities: PROGRESS-Plus factors can be used to identify factors that are known to socially stratify health opportunities and outcomes.[3,4] PROGRESS stands for: Place of residence, Race or ethnicity or culture or language, Occupation or out of work, Gender or sex, Religion, Education, Socioeconomic status, and Social capital. “Plus” recognizes additional factors including personal characteristics, time-dependent situations, and relationships. These characteristics may be associated with each other (for example, educational attainment and socioeconomic status) and may interact with each other (for example socioeconomic status and ethnicity) or with systems of oppression, power and privilege to drive the experience of health inequities (intersectionality).[5] The importance of these characteristics may depend on the context and setting. EQUALSS GUIDE MULTIPLE[6] is another tool that can be used, which includes: Ethnicity (and race), Qualifications and education, Underserved area, Age, Language and religion, Sex, Sexual orientation, Gender identification, Underrepresented groups (inclusion groups), Income and wealth, Disability (physical, mental, and learning), Employment and occupation, and Multiple disadvantage.

Populations experiencing inequities: Various terms have been used to describe populations that experience health inequities, ie, “inclusion health populations,”[7-9] “equity-seeking,” “equity-deserving,” “underserved,” “marginalized,” or “disadvantaged” populations.[10,11] In this reporting guideline, we utilize the term *populations experiencing inequities* to acknowledge individuals with firsthand experience of inequity, whether current or historical. This term emphasizes inclusivity and recognizes that inequities are not inherent to a given population, can evolve over time and are context dependent. Those with past and/or present experiences offer a comprehensive perspective on accessing care and services. We recognize the importance of using terms that best fit the study context and meet the needs of the populations experiencing inequities involved or impacted by the study.

Health equity data: Statistics and information related to health inequities as well as other characteristics associated with inequities.[12]

Lived experience of inequities: We adopted the Patient-Centered Outcomes Research Institute (PCORI)’s definition of individuals with lived experience of inequities, which includes patients, caregivers, clinicians, and other health care partners involved in patient care.[13]

Interest-holders: These are groups with legitimate interests in the health issue under consideration: patients, health research funders, health care service purchasers, peer-reviewed journal editors, policymakers, research leaders, review creators and commissioners, product developers, program overseers, care providers, and the general public.[14] The term *interest-holders* provides an alternative to *stakeholders* given its colonial roots and extensive consultation with the MuSE (previously known as Multi-Stakeholder Engagement) consortium to find a favored replacement.[15]

Knowledge users: Individuals who are likely to use the research results to make informed decisions about health policies, programs, and/or practices.[16] Knowledge users may originate from different interest-holder groups to ensure usefulness and relevance of the research.

Engagement: According to the MuSE consortium,[14] engagement can take 2 forms: shared decision-making, where individuals with lived experience of inequities contribute equally to decisions, or providing advice or feedback, where their interest-holder perspectives are sought and considered by the larger research team.

Downloaded from jamanetwork.com by guest on 07/17/2026

the health literature. This results in health decisions and policymaking that are unsuitable for many in our society.[26-31]

The coronavirus (COVID-19) pandemic has exposed significant inequities in health care access and outcomes.[32-34] The absence and inadequate reporting of health equity data and considerations in observational research are barriers to identifying and mitigating these inequities, impacting the populations who are in the greatest need of equitable interventions.[35-38] Therefore, addressing health inequities in research, especially observational studies,[39,40] is essential for ensuring that knowledge users have access to high-quality data that can guide equitable health care interventions.[41-45] This aligns with the global agenda of leaving no one behind—achieving Universal Health Coverage (UHC)[46] was among the Sustainable Development Goals (SDGs)[22] adopted by the United Nations—where generating data on approaches to reach populations experiencing inequities, or measurement at disaggregated levels, are fundamental. It also aligns with emerging evidence on benefits of climate change mitigation and adaptation.[47] This extends beyond matters of improved reporting; this is a call to action to ensure that research supports the equitable distribution of and access to health care resources and outcomes for all populations.

Reporting guidelines[48] provide evidence-informed guidance to authors for reporting essential information, enabling knowledge users to understand the research question, methodology, and findings. Research reported in adherence to reporting guidelines can enable readers to identify potential biases, weaknesses, and strengths in studies.[49,50] Extending the STROBE reporting guidelines for health equity will help enhance the clarity and completeness of reporting for health equity data and considerations in observational studies.[51]

Objectives

This guideline extends the STROBE reporting guidelines for specific attention to reporting health equity–relevant methods, data, and other considerations in observational studies. We describe the STROBE-Equity reporting guideline checklist and elaborate on each extension item.

Scope of STROBE-Equity Extension

The STROBE-Equity extension is designed to guide the reporting of health equity data and considerations in studies of any topic that aim to investigate differences in health that are avoidable and considered unjust (Box). The STROBE-Equity extension applies to the 3 observational study designs addressed in the STROBE statement: cohort, case-control, and cross-sectional studies. It is intended to complement the STROBE statement rather than replace it. Similarly, it can be used alongside the Reporting of Studies Conducted Using Observational Routinely Collected Data (RECORD)[52]guidelines. This extension focuses on transparent reporting, not to inform study design or implementation. Given the sensitive nature of health equity research, authors are encouraged to carefully consider how data are reported to avoid reinforcing stigma or discrimination. The guideline does not apply to qualitative studies as they require a different reporting structure.[53,54]

Development of the STROBE-Equity Reporting Guideline Checklist

To develop the STROBE-Equity extension, we adhered to a published protocol,[55] informed by the Enhancing the Quality and Transparency of Health Research (EQUATOR) Network's methodological framework for developing reporting guidelines.[56] We followed 2 parallel streams: one for the global guidance (described in this paper) and another for creating Indigenous guidance, led by Indigenous scholars. This paper presents the development of the global reporting guideline, covering phases 1 to 3 as specified in the protocol. Phases 4 and 5, which involve evaluating the outcomes of collaboration between the global and Indigenous streams and the outline the knowledge translation plan, will be reported separately. Patient and public engagement and the Delphi process are reported according to the Guidance for Reporting Involvement of Patients and the Public 2.0 (GRIPP2)[57] and Accurate Consensus Reporting Document (ACCORD)[58] reporting guidelines (eAppendix 1 in the Supplement).

Downloaded from jamanetwork.com by guest on 07/17/2026

A diverse, global multidisciplinary team with 57 members developed the STROBE-Equity extension that included members of the public, authors of observational studies, statisticians, social scientists, epidemiologists, methodologists, funders, health care practitioners, ethicists, and knowledge users. We sought diversity in our research team and steering committees across geography, genders, ethnicities, and career stages (eAppendix 2 in the Supplement). The group included knowledge users from the Pan American Health Organization/World Health Organization (PAHO/WHO), the Royal College of Physicians and Surgeons of Canada, scientific journal editors, the Canadian Drug Agency, and the Public Health Agency of Canada. All the team members were part of the Technical Oversight Committee, which oversaw the whole project. In parallel, we established a Knowledge User Committee (12 members) and a Patient and Public Steering Committee (3 members). Both committees were consulted at all stages of the guideline development process. An Executive Committee comprising the coprincipal investigators, trainees, and researchers (10 members) was responsible for leading the empirical studies. Recognizing the Indigenous right to self-determination as outlined in the United Declaration on the Rights of Indigenous Peoples (Article 3),[59] we established an Indigenous Steering Committee comprised of an Indigenous Elder, researchers, clinicians, and trainees (6 members). This Indigenous stream provides a culturally safe way to coproduce ethical and relevant guidance for reporting health equity in Indigenous health research. We describe our values and positionality statements in eAppendix 2 in the Supplement.

In Phase 1, the STROBE-Equity project team proposed 36 extension items to the STROBE reporting guideline that would be required to ensure transparent and complete reporting of health equity data and considerations. These items formed the interim guidance for the STROBE-Equity extension.[18] A scoping review of equity guidance found that all 36 items proposed in the interim guidance[18] were supported by existing guidance[60,61] and 13 additional items were identified (**Figure**). Then, a methodological review of health equity data and reporting and considerations in 320 observational studies indicated that the reporting of study design features ranged from 0% (0 of 320) to 95% (307 of 320) of studies, with over half of the items being reported by less than one-quarter.[30,62] We conducted 8 key informant interviews with 8 STROBE-Equity project group members from diverse backgrounds, genders, and ethnicities. These interviews identified 6 key themes that guided reflection, consideration, and confirmation of final checklist items. These themes highlighted the importance of clarity, feasibility, contextual relevance, and the inclusion of diverse perspectives to improve the reporting of health equity in observational research. A detailed table in eAppendix 3 in the Supplement outlines the themes and their impact on the development of the extension items.

In Phase 2, we sought to gather feedback from diverse interest-holders (eg, researchers, members of the public, individuals with lived experiences of inequities) with different backgrounds by deploying a global online survey from June 2023 to January 2024 (recruitment details and survey questions available in eAppendix 4 in the Supplement). The survey consisted of 24 recommendations for new items to STROBE, and 17 items for which no extension was proposed. We received 162 responses to the global online survey, with representation from different self-identifying knowledge users including research methodologists (58 of 162 [36%]), epidemiologists (33 of 162 [20%]), clinicians (22 of 162 [14%]), scientific journal editors (17 of 162 [10%]), policy makers (12 of 162 [7%]), and patients (9 of 162 [6%]) (eAppendix 5 in the Supplement). A third of the survey participants self-identified as individuals with lived experiences of health inequities. Seventy-six percent (123 of 162) had over 10 years of experience in health care research. Content expertise varied from public health and health promotion (52 of 162 [32%]) to clinical research (24 of 162 [15%]), equity-oriented research (13 of 162 [8%]), and health systems research (13 of 162 [8%]). We obtained 25% (41 of 162) of respondents from people based in low-and-middle income countries (eAppendix 5 in the Supplement). The survey showed over 80% agreement for 22 out of 24 proposed extension items (eAppendix 5 in the Supplement). However, 63% (85 of 135) to 75% (91 of 121) of respondents indicated that 17 items without proposed extension items should have extension items, suggesting a strong interest in further refinement. Open-ended comments indicated

Downloaded from jamanetwork.com by guest on 07/17/2026

endorsement for developing guidance describing minimal standards for health equity reporting. Additionally, participants felt a need for better definitions regarding populations experiencing health inequities.

In Phase 3, we convened the team members and all steering committee members as our consensus panel, using 2 online consensus meetings held at different times to be inclusive of multiple time zones. We carried out a preconsensus survey to evaluate inclusion of items with under 70% agreement and help focus the consensus discussion on items where people held different opinions. Survey participants contributed detailed comments to support their judgements, which were synthesized into main themes and presented at the consensus meeting. We then held two 1.5-hour online consensus meetings to discuss and finalize the checklist items. The consensus threshold was defined a priori as 80% agreement or higher among 80% of the respondents. The consensus meetings endorsed 21 items with at least 80% agreement; and 2 items were rejected based on consensus that they were adequately covered and 1 item was contested (eAppendix 6 in the Supplement). We established a synthesis working group to consolidate the feedback from the consensus meetings and discuss how to increase the feasibility of health equity reporting while safeguarding items the working group believed were essential for good practice. This team consisted of 10 project members who convened for two 1-hour meetings and discussed drafts by email. The

Figure. Flow of Proposed STROBE-Equity Extension Items

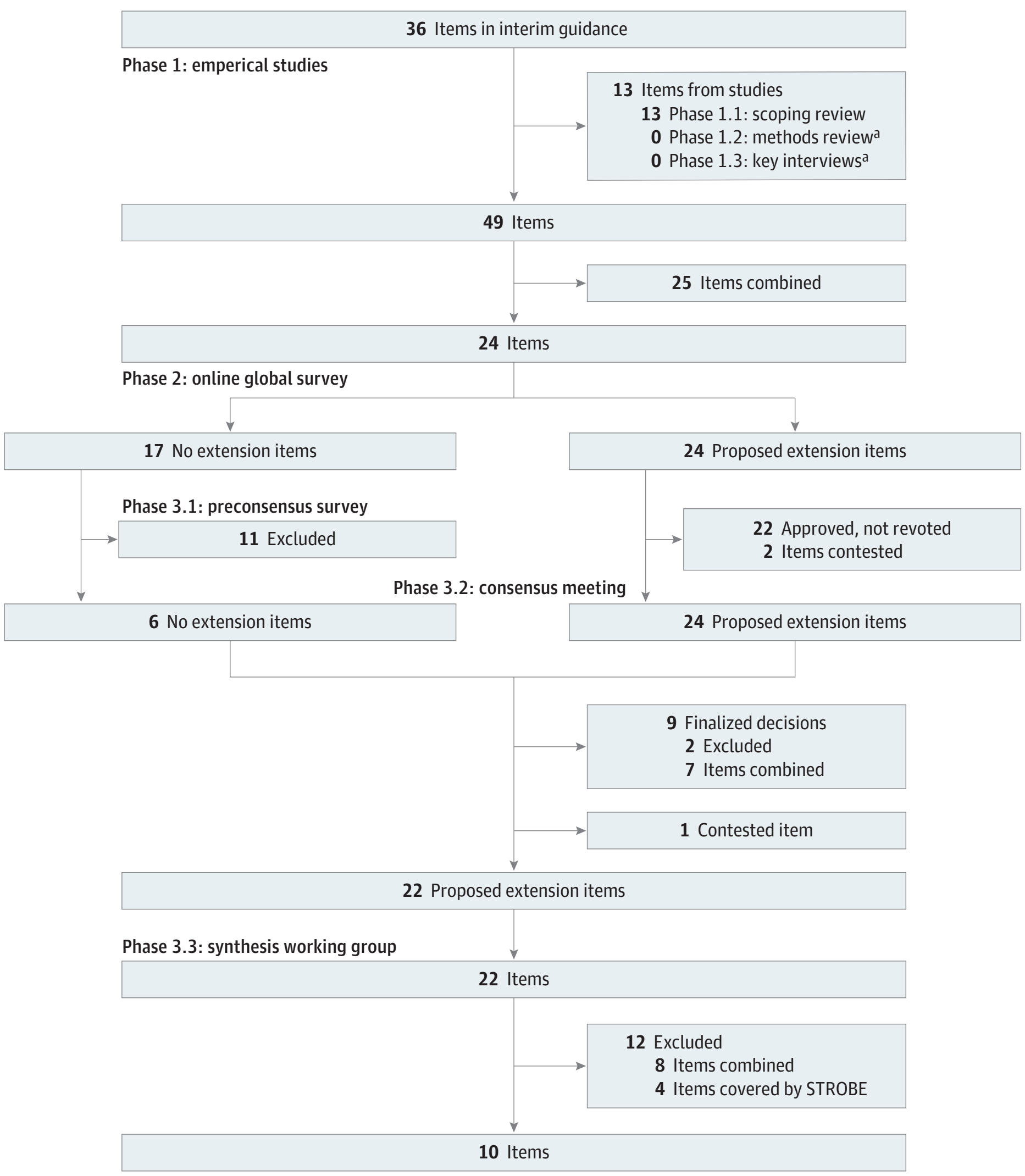

STROBE indicates Strengthening the Reporting of Observational Studies in Epidemiology.

[a] The methods review aimed to inform current reporting practices in observational studies, while the key informant interviews identified principles to guide the consensus meeting and finalize the extension checklist, but neither were intended to identify new items. “Items combined” refers to collapsing at least 2 items into a single item to enhance clarity and reduce redundancy.

Downloaded from jamanetwork.com by guest on 07/17/2026

groups' focus was to improve clarity of wording, maximize parsimony and maintain essential items. This team was guided by values and principles expressed during the consensus panel discussions and themes identified in the key informant study. Additional details are in eAppendix 7 in the Supplement.

Patient and Public Involvement

Following the GRIPP2 reporting checklists (eAppendix 1 in the Supplement),[63] we involved both patients and the public at various stages of the development of the guideline. Our Patient and Public Steering Committee, led by 3 of our team members (J.T., R.G.S., and H.E.) who were also decision-makers in the development of the STROBE-Equity extension. Two team members who have lived experience with health inequities and prior participation in health equity research (J.T. and R.G.S.) were involved from the funding acquisition stage. They convened every 2 months to guide the project's design and received regular project updates through quarterly technical advisory meetings and steering committee sessions, with study progress shared via project newsletters. The patient and public partners played a pivotal role in establishing the need for these guidelines and refining the STROBE-Equity guideline by identifying areas for integrating equity considerations. They also contributed to designing the 3 empirical studies and discussions regarding interpreting the key findings. The patient and public partners received annual financial compensation for their contributions during the project. Additional patient and public perspectives were gathered through the key informant interviews and the online global survey.

Checklist Items: Extension of the STROBE Statement

The **Table** shows the original STROBE checklist (22 items) alongside the STROBE-Equity extension items. STROBE-Equity introduces 10 items that are specific to health equity–relevant observational studies. The items apply to the 3 study designs covered by the original STROBE statement. Identical to the STROBE statement, the items in the STROBE-Equity guidelines are organized into sections: Title and Abstract (1.1), Introduction (2.1), Methods (4.1-12.1), Results (13.1-14.1), and Discussion (18.1). Authors of health equity–relevant observational studies should address all items of the STROBE Equity checklist (although some information may be best placed in supplementary materials, if journal space constraints apply).

Title and Abstract

1.1 Highlight in the Title or the Abstract the Relevance of Equity to the Study Objective

Example 1, study title: "Trends in Racial and Ethnic Disparities in Barriers to Timely Medical Care Among Adults in the US, 1999 to 2018."[64]

Example 2, abstract objective: "To identify variation in several measures of health equity from 1993 to 2017 in the United States and to test whether there are significant time trends."[65]

Example 3, abstract methods: "We use population at risk and mortality data covering 1991-2019 to calculate the mortality rate at age 65-69 at the Local Authority level. We use the 2019 Index of Multiple Deprivation to examine geographical inequalities."[66]

Example 4, abstract results: "Those with a foreign first language had lower testing rates, higher incidence, and higher positivity."[67]

Example 5, abstract discussion: "Management of high fasting plasma glucose remains a major challenge for older people with T1DM, and targeted clinical guidelines are needed."[68]

Reporting health equity objectives in the title or the abstract helps patients, policymakers, and practitioners discover health equity–relevant studies by making them easily identifiable and understand how they relate to health equity.[60] This is especially important because health equity studies often focus on specific populations,[30,69] such as those defined by PROGRESS-Plus, and the

Downloaded from jamanetwork.com by guest on 07/17/2026

Table. Equity Extension to the STROBE Statement

STROBE item	Item No	STROBE recommendation	STROBE-Equity extension recommendation
Title and abstract	1	• Indicate the study's design with a commonly used term in the title or the abstract<br>• Provide in the abstract an informative and balanced summary of what was done and what was found	1.1 Highlight in the title or the abstract the relevance of equity to the study objective.
**Introduction**			
Background/rationale	2	Explain the scientific background and rationale for the investigation being reported	2.1 Describe the relevance of equity to the study rationale and motivation.
Objectives	3	State specific objectives, including any prespecified hypotheses	STROBE guidelines sufficient
**Methods**			
Study design	4	Present key elements of study design early in the paper	4.1 Report on whether and how individuals with lived experiences of inequities were engaged in the study conceptualization, design, conduct, or dissemination.<br>4.2 Report whether and how the research team composition includes individuals with lived experience of inequities.
Setting	5	Describe the setting, locations, and relevant dates, including periods of recruitment, exposure, follow-up, and data collection	5.1 Report whether methods of sampling and recruitment were tailored to populations with lived experience of inequities.
Participants	6	General:<br>• For cohort studies, give the eligibility criteria, and the sources and methods of selection of participants. Describe methods of follow-up<br>• For case-control studies, give the eligibility criteria, and the sources and methods of case ascertainment and control selection. Give the rationale for the choice of cases and controls<br>• For cross-sectional studies, give the eligibility criteria, and the sources and methods of selection of participants Matched studies:<br>• For cohort studies, for matched studies, give matching criteria and number of exposed and unexposed<br>• For case-control studies, for matched studies, give matching criteria and the number of controls per case	6.1 Describe whether characteristics associated with inequities were considered in the process of selecting participants (eg, eligibility criteria, matching).
Variables	7	Clearly define all outcomes, exposures, predictors, potential confounders, and effect modifiers. Give diagnostic criteria, if applicable	STROBE guidelines sufficient
Data sources/measurement	8	For each variable of interest, give sources of data and details of methods of assessment (measurement). Describe comparability of assessment methods if there is more than one group	STROBE guidelines sufficient
Bias	9	Describe any efforts to address potential sources of bias	STROBE guidelines sufficient
Study size	10	Explain how the study size was arrived at	STROBE guidelines sufficient
Quantitative variables	11	Explain how quantitative variables were handled in the analyses. If applicable, describe which groupings were chosen and why	STROBE guidelines sufficient
Statistical methods	12	• Describe all statistical methods, including those used to control for confounding<br>• Describe any methods used to examine subgroups and interactions<br>• Explain how missing data were addressed<br>• For cohort studies, explain how loss to follow-up was addressed<br>• For case-control studies, explain how matching of cases and controls was addressed<br>• For cross-sectional studies, describe analytical methods taking account of sampling strategy<br>• Describe any sensitivity analyses	12.1 Describe all analytical methods related to equity objectives (eg, subgroups, sensitivity).
**Results**			
Participants	13	• Report numbers of individuals at each stage of study—eg, numbers potentially eligible, examined for eligibility, confirmed eligible, included in the study, completing follow-up, and analyzed<br>• Give reasons for nonparticipation at each stage<br>• Consider use of a flow diagram	13.1 Describe participant flow including losses, exclusions, non-responses and unplanned pauses according to characteristics associated with inequities.
Descriptive data	14	• Give characteristics of study participants (eg, demographic, clinical, social) and information on exposures and potential confounders<br>• Indicate number of participants with missing data for each variable of interest<br>• For cohort studies, summarize follow-up time (eg, average and total amount)	14.1 Report characteristics of study participants, missing data and follow-up time across characteristics associated with inequities.
Outcome data	15	• For cohort studies, report numbers of outcome events or summary measures over time<br>• For case-control studies, report numbers in each exposure category, or summary measures of exposure<br>• For cross-sectional studies, report numbers of outcome events or summary measures	STROBE guidelines sufficient
Main results	16	• Give unadjusted estimates and, if applicable, confounder-adjusted estimates and their precision (eg, 95% confidence interval). Make clear which confounders were adjusted for and why they were included<br>• Report category boundaries when continuous variables were categorized<br>• If relevant, consider translating estimates of relative risk into absolute risk for a meaningful time period	STROBE guidelines sufficient
Other analyses	17	Report other analyses done—eg, analyses of subgroups and interactions, and sensitivity analyses	STROBE guidelines sufficient

(continued)

Downloaded from jamanetwork.com by guest on 07/17/2026

Table. Equity Extension to the STROBE Statement (continued)

STROBE item	Item No	STROBE recommendation	STROBE-Equity extension recommendation
**Discussion**			
Key results	18	Summarize key results with reference to study objectives	18.1 Interpret the results while considering the representativeness of the study sample and the implications of the findings on health equity.
Limitations	19	Discuss limitations of the study, taking into account sources of potential bias or imprecision. Discuss both direction and magnitude of any potential bias	
Interpretation	20	Give a cautious overall interpretation of results considering objectives, limitations, multiplicity of analyses, results from similar studies, and other relevant evidence	
Generalizability	21	Discuss the generalizability (external validity) of the study results	
**Other information**			
Funding	22	Give the source of funding and the role of the funders for the present study and, if applicable, for the original study on which the present article is based	STROBE guidelines sufficient

Abbreviation: STROBE, Strengthening the Reporting of Observational Studies in Epidemiology.

findings should clearly describe the populations studied and any limitations in their applicability to other settings. Clear reporting ensures that the study's reference and potential limitations in the applicability of the findings to other settings are easily understood. Moreover, making equity-relevant studies easily searchable in databases like MEDLINE or Embase, as indexing terms (eg, MeSH or Emtree) are influenced by the terms used in the title and abstract, which also guide search strategies.

We recommend authors incorporate specific terms, like health equity or other more specific terms in the title, when appropriate.[70,71] Additionally, the relevance of health equity should be highlighted across different sections of the abstract (ie, as an objective that to emphasize equity-relevant results or interpret findings while considering the impact on health equity). To further improve searchability, authors and journals are encouraged to describe their work using appropriate standardized subject terms (MeSH or Emtree). For example, MeSH terms for the concept of health equity include *health equity*, *health inequities*, and *health status disparities* for indexing.[72] Each concept of a robust search strategy should consist of text words and subject terms.[73]

Introduction

2.1 Describe the Relevance of Equity to the Study Rationale and Motivation

Example 1: "Marginalized populations have approximately 5 times greater risk of adverse COVID-19 consequences related to social determinants of health that may exacerbate patient comorbidities. Equitable distribution would eliminate vaccination disparities while mitigating the disproportionate effect of the COVID-19 pandemic in underserved populations, which are disadvantaged because of limited access to health care, low socioeconomic status, or race."[74]

Example 2: "Substantial racial and ethnic disparities persist in cancer survival, reflecting systemic barriers to cancer screening, treatment, and survivorship care and differences in the prevalence of comorbidities."[75]

Example 3: "The importance of non-traditional risk factors for atherosclerotic diseases, such as socioeconomic deprivation, has also been increasingly recognised. Whether socioeconomic deprivation is as strongly associated with other CVDs [cardiovascular diseases] as with atherosclerosis is uncertain, but it is important to understand as many countries have reported an increase in socioeconomic inequalities."[76]

Describing how the aim of health equity relates to the author's motivation for conducting the study clarifies its purpose and demonstrates its contribution to addressing health inequities.[77] Understanding these motivations helps contextualize the research design and explains why specific research questions were prioritised.[78,79]

We advise authors to emphasize the relevance of the health condition to populations experiencing inequities, using context-specific language and acknowledging the role of

Downloaded from jamanetwork.com by guest on 07/17/2026

intersectionality[80-82] and systemic causes of health inequities (eg, racism, sexism, heterosexism, ableism, and colonialism). Authors should provide a rationale that elucidates the underlying theory for why the issue is particularly relevant to these populations, indicating where differences reflect inequities.[83,84] Using a logic model to illustrate this theory can further clarify the study's equity-focused objectives.[85,86]

Methods (Study Design)

4.1 Report on Whether and How Individuals With Lived Experiences of Inequities Were Engaged in the Study Conceptualization, Design, Conduct, or Dissemination

Example 1: "The survey was refined in consultation with people with lived experience of mental health conditions involving a citizen's jury participatory methodology process. Items were modified, and questions added to reflect the Canadian context, with the aim to examine indicators of mental health, stress, and coping in the previous two weeks among the Canadian population 18 years and older during the COVID-19 pandemic."[87]

Example 2: "The study was conceptualized and led by the first author (M.K., Wiradjuri woman), in partnership with NAATSIHWP and Aboriginal communities represented by: H.L., S.B., P.H., K.B."[88]

Example 3: "Study findings will be disseminated through the Provincial Advisory Committee that was assembled to oversee the entire Provincial Prescribed Safer Supply evaluation project. The committee includes people with lived/living experience of overdose and/or substance use who have been engaged in this project from its establishment. We will also disseminate results through the British Columbia Overdose Emergency Response Centre and social media posts through our organizational partners such as the Centre for Advancing Health, British Columbia Centre for Disease Control, and Canadian Institutes for Substance Use Research."[89]

Example 4: "Although formal patient and public involvement was not integrated into the study's design, we engaged with the public by inviting a patient to review our manuscript, whose insights contributed to refining our presentation and interpretation of findings."[90]

The reporting of ethical research necessitates respecting the voices and experiences of those directly affected by the study.[91-95] This includes reporting on any engagement of individuals with lived experiences in the conduct or execution of the study. Not only is this a demonstration of researchers' accountability to their ethical responsibility,[96-98] it reflects a clear commitment to prioritizing health equity by describing the efforts put in place to engage those experiencing health inequities.[99] It also signals to readers, funders, and interest-holders that the study is relevant to these populations.[100] Transparent reporting offers readers an opportunity to assess the level of engagement and determine whether it was meaningful or tokenistic.[101-106] Where engagement was not undertaken, researchers should reflect on how its absence may affect the study's findings.[107]

Authors should report who was engaged, what were the strategies for engagement, how people with lived experience were involved (shared decision-making, providing advice or feedback, collaborating, informing, or other), and the reasons behind these decisions. Following standards for reporting engagement such as the GRIPP2 checklist[57] or other standards for specific populations (eg, Indigenous Peoples[108,109]) can guide authors in reporting these practices comprehensively. We reported how our engagement process for this guideline adheres to the GRIPP2 checklists in eAppendix 1 in the Supplement.

4.2 Report Whether and How the Research Team Composition Includes Individuals With Lived Experience of Inequities

Example 1: "People with lived or living experience of substance use and/or overdose from across British Columbia were engaged as research assistants and advisors in the broader evaluation project of which this study is a part."[89]

Downloaded from jamanetwork.com by guest on 07/17/2026

Example 2: "This project is informed, implemented, and reported by an Indigenous-led research team, we recognize that our lived experience and worldviews influence the way that the study was conducted which is central to developing an Indigenous-led evidence base for SCC [smoking cessation care]. Our team brings Aboriginal and Torres Strait Islander lived experience (M.K., K.B., H.L., S.B.), Indigenous lived experience (R.M.), expertise in Aboriginal health services (P.H., H.L., S.B.), Aboriginal Health Workers (H.L., S.B., K.B.), Indigenous tobacco research (A.M., M.K., R.M., Y.B.Z.) and epidemiology (R.M., Y.B.Z.)."[88]

Example 3: "Relationality is foundational to Indigenous research practice and paradigm. We recognize that our own lived experience and standpoint are central to this research including the conceptualization, development, conduct, and interpretation of findings. Our team embodies Aboriginal and Torres Strait Islander lived experience (M.K., C.C., K.C., J.F., and K.R.B.), Indigenous lived experience (R.M.), expertise in Indigenous tobacco research (M.K., C.C., R.M., A.G.M., C.S., and B.B.), Aboriginal health services (B.D.), qualitative research (M.K., K.B., and C.C.), and tobacco behavioral counseling (J.F. and C.S.). This study privileges Indigenous knowledge and recognizes the ongoing scientific rigor of Aboriginal and Torres Strait Islander people and their knowledge systems."[110]

The composition of a research team plays a critical role in determining how the research is conducted, interpreted, and applied.[111-114] Including voices of individuals with lived experience of health inequities may help the research address the realities of those affected by health inequities.[115-117] Reporting the composition of the research team, particularly in terms of including individuals with lived experiences of inequities, helps readers understand how power dynamics,[117-119] bias,[96,120] and contextual factors[116,121] shape the conducted research. This is especially important in health equity research, where team diversity can directly influence the framing and interpretation of the findings.[122]

Authors should construct a statement that specifies the team's representation and roles, particularly focusing on expertise relevant to the health equity objectives of the study. During our consensus meeting discussions, it was noted that disclosing this information, particularly at the individual level, could compromise individuals' privacy and expose them to stigma, discrimination, or potential harm. Therefore, a possible approach to mitigate this would be to report the data in aggregate for the whole team. We report our team composition statement as an example in eAppendix 2 in the Supplement.

Methods (Setting)

5.1 Report Whether Methods of Sampling and Recruitment Were Tailored to Populations With Lived Experience of Inequities

Example 1: "We plan to recruit 1000 participants in the GTA [Greater Toronto Area] and 500 participants in Ottawa using a peer recruitment strategy. Survey venues that are frequented by ACB [African, Caribbean, and Black] people will be selected with input from local team members and collaborators. This form of recruitment should generate a sample that is similar in age, gender, and country of birth to Statistics Canada data pertaining to the GTA and Ottawa. These approaches have been used in previous studies to limit selection bias and create representative samples."[123]

Example 2: "Quota sampling will be used for the quantitative surveys. The Spanish survey (n = 500) will include 250 SGM [sexual and gender minorities] and 250 non-SGM. The English survey (n = 3000) will include non-Hispanic Black (1000/3000, 33%), non-Hispanic White (1000/3000, 33%), and Hispanic or Latino/a/x persons of any race (1000/3000, 33%), with 50% (250/500 for Spanish and 1500/3000 for English) of each group being composed of SGM. We will further stratify recruitment by gender to generate sample sizes of approximately 250 at each race and ethnicity × SGM × gender intersection (eg, White non-SGM women). Transgender and gender nonbinary respondents will be grouped by gender identity (eg, transgender men and transmasculine persons with men)."[124]

Downloaded from jamanetwork.com by guest on 07/17/2026

Example 3: "First, to accommodate a relatively young Latino population and, therefore, a large proportion of working-age subjects, both field and clinic schedules included weekdays, weekday evenings, and weekends. Second, to accommodate language barriers and trust issues, given that many participants are first-generation immigrants who do not speak English and a relatively high prevalence of illegal immigrants, all field and clinic staff were Latino and fully bilingual. Third, because many younger adult Latinos in Los Angeles do not have access to childcare facilities and, therefore, have nowhere to leave their children when asked to come to the clinic, we provided in-clinic free childcare. Finally, because many older Latinos do not drive and have no transportation to get to the clinic for an appointment, we offered free transportation to and from the clinic."[125]

Tailoring sampling or recruitment strategies helps to minimize biases that may arise from traditional sampling methods,[126-130] which might not reach populations experiencing inequities.[131-134] Reporting these methods allows for transparency in how the researchers actively sought to reach and include populations experiencing inequities by adapting their recruitment strategies.[135] This is particularly important when the populations of interest are typically underrepresented in studies,[98] as it clarifies how efforts were made to achieve inclusivity.[98]

Authors should report how their sampling or recruitment strategies were adapted to improve the representation of populations experiencing health inequities, including the rationale behind their choices. In the case of sampling, authors should describe their sampling approach (eg, proportionate stratification vs disproportionate stratification) and explain how it aligns with answering research questions related to health equity. The use of recruitment in the study design requires authors to describe if they used specific outreach methods, location-based recruitment strategies, or tailored recruitment processes to address language, cultural and logistical barriers, and disabilities in order to have a representative sample.

Methods (Participants)

6.1 Describe Whether Characteristics Associated With Inequities Were Considered in the Process of Selecting Participants (eg, Eligibility Criteria, Matching)

Example 1: "To account for differences in baseline characteristics between the 2 groups [males and females], a propensity score–matching (PSM) model was developed using logistic regression to derive two well-matched groups for comparative outcome analysis [from registry data]. Variables included in the PSM model included age, race, and key comorbidities (hypertension, diabetes, chronic obstructive lung disease, heart failure, obesity, nicotine dependence, and history of stroke)."[136]

Reporting whether characteristics associated with health inequities were considered in the selection process provides insights regarding the representation of the study sample to populations most affected and informs the applicability of the study's results to these populations.[137,138] Doing so allows readers to assess whether imbalances in these factors could have influenced the study findings or biased their interpretation. In certain contexts, characteristics such as race or ethnicity in the US, are often proxies for social determinants, such as systemic racism.[139,140] Thus, reporting them helps with contextualizing the results with a more nuanced and deeper understanding of potential biases and disparities[141] and informs the relevance of the findings to other settings.

Authors should describe the rationale for including or excluding specific populations with characteristics associated with inequities in the selection process. This may include the eligibility criteria, propensity score (when matching in case-control and controlled cohort studies), or adjusting eligibility criteria to ensure balanced groups. In line with the original STROBE, authors should describe the source of the study population (eg, the general population of a region or country), the method of recruitment (eg, referral or self-selection through advertisements), and details regarding follow-up procedures. Additionally, they should report whether efforts to minimize nonresponse and loss to follow-up were used, as well comment on the consistency of study procedures for all the included participants.

Downloaded from jamanetwork.com by guest on 07/17/2026

Methods (Statistical Methods)

12.1 Describe All Analytical Methods Related to Health Equity Objectives (eg, Subgroups, Sensitivity)

Example 1: "We calculated hazard ratios (HR) and 95%CI to estimate the difference in SARS-CoV-2 incidence between ethnic groups, using the Dutch origin group as a reference, adjusting for current age in years and sex."[142]

Example 2: "Thirdly, as boys are more likely to receive a diagnosis of neurodevelopmental disorders than girls, we evaluated the association by sex of the infants."[143]

Additional analyses are frequently conducted to assess whether effects or associations differ across groups experiencing inequities (eg, based on race, gender, socioeconomic status, or other relevant factors),[144,145] or to assess how potential biases could obscure the true impact of inequity-related characteristics on outcomes.[146] Reporting these analyses allows readers to understand how differences between groups were evaluated and whether any findings could indicate a reduction or increase of health inequities.

Authors should clearly state whether subgroup analyses were preplanned or exploratory, as post-hoc analyses can introduce bias.[147,148] They should describe the methods used to examine differences between groups and the assumptions underlying them. For example, authors should report whether interaction tests were conducted, specify how they were reported,[149] and report if any sensitivity analyses were performed to assess the robustness of the findings. Consideration should be given to how the choice of presenting analyses—whether in absolute or relative terms—can significantly influence judgments about whether health inequities are widening or narrowing.[150,151]

Results (Participants)

13.1 Describe Participant Flow Including Losses, Exclusions, Nonresponses and Unplanned Pauses According to Characteristics Associated With Inequities

Example 1: "We excluded individuals with missing information on race/ethnicity (n = 112) and those who identified as Alaskan Native or American Indian or 'other' race (n = 6673) due to small numbers."[64]

People experiencing health inequities may be less inclined to participate in studies and more inclined to withdraw than other populations.[152-155] Explaining why participants were lost to follow-up, excluded, or failed to respond, and how these factors relate to characteristics of people experiencing inequities, allows readers to judge the representativeness of the final sample. Additionally, this detailed reporting helps identify whether biases related to inequities were potentially introduced in the study[156] and highlights what needs to be done in future studies for populations experiencing inequities.

Authors should explain the reasons behind participant loss, exclusions, or nonresponses, especially about characteristics linked to inequities. Authors could present this data through a flow diagram or narrative description in text.[157] Such a flow diagram could become challenging to make if the equity factor involves more than 2 levels or if multiple characteristics of interest, such as poverty and rurality, are considered. Using tables may more effectively convey this information.

Results (Descriptive Data)

14.1 Report Characteristics of Study Participants, Missing Data, and Follow-Up Time Across Characteristics Associated With Inequities

Example 1: "The 8.5% of BAME [Black, Asian, and minority ethnic] is the sum of approximately 2% Bangladeshi, Indian and Pakistani (BIP), and 6.5% of other minority ethnic groups (non-BIP: White Other, Mixed, Black, Asian, and Arab)."[158]

Example 2: "We included 3 574 903 individuals who did not have atrial fibrillation at index age 45 years or older, of whom 1 727 703 were men (48.3%) and 1 847 200 were women (51.7%)."[159]

Downloaded from jamanetwork.com by guest on 07/17/2026

Disaggregating baseline characteristics of study participants and examining how these characteristics relate to the exposure, allows readers to better assess the applicability[160,161] and representation[162-166] of different populations in the study. Depending on the study design, this data can highlight potential biases in health care practices[120,167] and provides insight into the effectiveness of recruitment strategies focused on specific populations, if used. Additionally, stratifying follow-up time by relevant characteristics offers a clearer understanding of how follow-up duration and missing data may differ across population groups,[168,169] improving the overall interpretation of the findings.

Authors should present baseline characteristics disaggregated by characteristics of interest associated with health inequities, such as socioeconomic status or ethnicity, and explain how these factors relate to the exposure of interest. Authors should report the number of participants with missing data and follow-up time stratified by these characteristics.

Discussion

18.1 Interpret the Results While Considering the Representativeness of the Study Sample and the Implications of the Findings on Health Equity

Example 1: "Our male-to-female breakdown was representative of the national sample, of which 15% of the Brazilian Society of Head and Neck Surgery members are females."[170]

Example 2: "Also, our online survey study design excluded individuals who have no access to internet, especially rural residents. Thus, our sample cannot be considered representative of the Chinese population, which might generate biased results. The rural residents recruited in our study might have higher social economic status than other rural residents because our rural sample have regular access to internet."[171]

Interpreting study results helps the reader understand the implications for health equity.[144,172] For instance, if a study's sample does not adequately reflect the populations most affected by health inequities, the results may not be generalizable or applicable to those populations, potentially leading to biased conclusions.[173-175]

The discussion of the findings should reflect on the results of health equity objectives. Authors can highlight any gaps in the study or emphasize significant findings. Authors need to consider the study's limitations, such as method of data collection, whether populations experiencing inequities were under- or overrepresented, and whether data collection approaches could lead to selection bias. Interpretations of the results should be made with caution to avoid overinterpretation or misinterpretation, as previously observed with race or ethnicity and several health outcome differences,[176] and to link them to the social determinants of health.[177-179] Identified biases should be considered when interpreting of the findings for accurate reporting. Furthermore, when comparing findings with previous studies, authors may explain how their results contribute to and impact the existing evidence base on health equity.

Discussion

The STROBE-Equity extension was developed to address a recognized need for improved reporting of health equity data and considerations in observational studies. It is designed to complement the original STROBE statement and checklist.

This extension aligns with the existing guidelines for reporting on health equity in randomized controlled trials (CONSORT-Equity) and systematic reviews (PRISMA-Equity) developed by members of our team and clarifies the process for conducting equity-relevant research. We will include the STROBE-Equity extension on the EQUATOR Network's library of reporting guidelines. To promote its adoption, we will engage the editors-in-chief of scholarly journals in epidemiology and global health, encouraging them to endorse and require the extension for equity-relevant studies. We have established a knowledge translation working group to develop strategies to ensure widespread

Downloaded from jamanetwork.com by guest on 07/17/2026

dissemination and use, such as road-testing[180] the reporting guideline and its translations and producing various patient-oriented tools such as digital stories. We are also surveying our team members regarding the perceived benefits, challenges, and impact of our integrated knowledge translation approach on the development process.

Strengths and Limitations

We followed a prespecified process to gather evidence on the necessity of the reporting guideline extension[55] and adhered to widely accepted methods for developing reporting guidelines.[56] We engaged a diverse group of 57 individuals, employed empirical studies, and obtained input from 162 individuals through an online survey, capturing a wide range of perspectives. The robustness and inclusivity of our approach, combined with high agreement with the extension items, enhances the guideline's applicability across diverse populations and settings. Nonetheless, we recognize that no guideline can comprehensively include the perspectives of all possible populations and situations where minor adaptations to the guideline may arise.

Additionally, our collective understanding of health equity in research is rapidly evolving with frequent new methodological developments, reflecting advancements in understanding how health equity can be conceptualized,[181] what are the factors at different levels that create inequities,[182] and how to overcome them.[183] We recognize that these guidelines may need to be updated to encompass evolving concepts, such as intersectionality,[5] as well as considerations for certain populations.

We acknowledge that the ability of researchers to report on health equity data and considerations is limited by the availability, quality, and type of data they are able to collect (ie, culturally sensitive data, lack of standardization).[4,184] Reporting these challenges could help bring these obstacles to light and foster opportunities for developing innovative solutions.

Conclusions

The STROBE-Equity extension provides essential guidelines for the reporting of health equity data and considerations in observational studies. We recommend using the STROBE-Equity extension alongside the main STROBE statement to enhance the clarity and completeness of health equity reporting in research. We encourage all interest-holders to promote using this extension to improve the evidence base for health equity.

ARTICLE INFORMATION

Accepted for Publication: June 25, 2025.

Published: September 3, 2025. doi:10.1001/jamanetworkopen.2025.32512

Open Access: This is an open access article distributed under the terms of the CC-BY License. © 2025 Dewidar O et al. *JAMA Network Open*.

Corresponding Author: Omar Dewidar, MSc, Bruyère Health Research Institute, University of Ottawa, Ottawa, ON K1R 6M1, Canada (Odewi090@uottawa.ca).

Author Affiliations: Temerty Faculty of Medicine, University of Toronto, Toronto, Ontario, Canada (Dewidar); Bruyère Health Research Institute, University of Ottawa, Ottawa, Ontario, Canada (Dewidar, Ghogomu, Nkangu, Tugwell, Welch); Knowledge Translation Program, Li Ka Shing Knowledge Institute, St Michael's Hospital, Unity Health Toronto, Toronto, Ontario, Canada (Shamseer); Faculty of Health and Life Sciences, University of Exeter, Exeter, United Kingdom (Melendez-Torres); Department of Internal Medicine, American University of Beirut, Beirut, Lebanon (Akl); Department of Health Research Methods, Evidence, and Impact, McMaster University, Hamilton, Ontario, Canada (Akl, Mbuagbaw); International Centre for Eye Health, London School of Hygiene and Tropical Medicine, London, United Kingdom (Ramke); School of Optometry and Vision Science, University of Auckland, Auckland, New Zealand (Ramke); Lee Kong Chian School of Medicine, Nanyang Technological University, Singapore (Wang); Sefako Makgatho Health Sciences University, Pretoria, South Africa (Oloyede); Centre for Evidence Based Health Care, Department of Global Health, Stellenbosch University, Cape Town, South Africa

Downloaded from jamanetwork.com by guest on 07/17/2026

(Young, Kredo, Wiysonge); Methodological and Implementation Research, Ottawa Hospital Research Institute, Ottawa, Ontario, Canada (Nicholls); Department of Community Health Sciences, Cumming School of Medicine, University of Calgary, Calgary, Alberta, Canada (Marshall); School of Medicine and Public Health, The University of Newcastle, Callaghan, New South Wales, Australia (Kennedy); Dalla Lana School of Public Health, University of Toronto, Toronto, Ontario, Canada (Hardy, Juando-Prats); Well Living House, Li Ka Shing Knowledge Institute, Unity Health Toronto, Toronto, Ontario, Canada (Hardy); School of Psychology, University of Ottawa, Ottawa, Ontario, Canada (Rizvi); Canadian Agency for Drugs and Technologies in Health, Ottawa, Ontario, Canada (Rader); Planetary Health Group, Department of Population Health, London School of Hygiene and Tropical Medicine, London, United Kingdom (Waddington); School of Epidemiology and Public Health, Faculty of Medicine, University of Ottawa, Ottawa, Ontario, Canada (Shea, Nkangu, Horsley, Little, Tugwell, Welch); Department of Law and Legal Studies, Carleton University, Ottawa, Ontario, Canada (Ellingwood); Public representative, Ottawa, Ontario, Canada (Ellingwood); The University of Newcastle, School of Medicine and Public Health, Callaghan, New South Wales, Australia (Wolfenden); Hassanah Consulting, Seattle, Washington (Tufte); Public representative, Seattle, Washington (Tufte); Royal College of Physicians and Surgeons of Canada, Research, Ottawa, Ontario, Canada (Horsley); Family Medicine, Dalhousie University, Halifax, Nova Scotia, Canada (Pottie); Unit of Science and Knowledge for Impact, Department of Evidence and Intelligence for Action in Health, Pan American Health Organization, Washington, DC (Cuervo); Doctoral Programme on Methodology of Biomedical Research and Public Health, Department of Paediatrics, Obstetrics, Gynaecology and Preventive Medicine and Public Health, Autonomous University of Barcelona, Bellaterra, Cataluña, Spain (Cuervo); School of Nursing, Faculty of Health and Behavioral Sciences, Lakehead University, Toronto, Ontario, Canada (Juando-Prats); Li Ka Shing Knowledge Institute, Unity Health Toronto, Toronto, Ontario, Canada (Juando-Prats); Department of Community Health and Epidemiology, Faculty of Medicine, Dalhousie University, Halifax, Nova Scotia, Canada (Feng); School of Public Health, University of Saskatchewan, Saskatoon, Saskatchewan, Canada (Feng); Department of Public Health and Epidemiology, School of Population Health, RCSI University of Medicine and Health Sciences, Dublin, Ireland (Sharp); Institute for Rural Development and Innovation Studies, Kwame Nkrumah University of Science and Technology, Kumasi, Ghana (Owusu-Addo); School of Health and Human Performance, Center for Health and Social Issues, Georgia College and State University, Milledgeville (Francis); Health Systems Research Unit, South African Medical Research Council, Cape Town, South Africa (Kredo, Wiysonge); Department of Epidemiology and Biostatistics, Institute of Public Health, Kilimanjaro Christian Medical College, Moshi, Tanzania (Mahande); Indigenous Health Equity Unit, Melbourne School of Population and Global Health, The University of Melbourne, Melbourne, Victoria, Australia (Chamberlain); Judith Lumley Centre, School of Nursing and Midwifery, La Trobe University, Melbourne, Victoria, Australia (Chamberlain); Family Medicine Department, School of Medicine, Pontificia Universidad Católica de Chile, Santiago, Chile (Pantoja); Cochrane Switzerland, and Cochrane Germany Foundation, Freiburg, Germany (von Elm); Centre for Global Child Health, Hospital for Sick Children, Toronto, Ontario, Canada (Bhutta); Institute for Global Health and Development, The Aga Khan University, Karachi, Pakistan (Bhutta); Ottawa Hospital Research Institute, Ottawa, Ontario, Canada (Tugwell); Faculty of Medicine, University of Ottawa, Ottawa, Ontario, Canada (Tugwell); Department of Family Medicine, Queen's University, Kingston, Ontario, Canada (Funnell); Department of Family Medicine, Faculty of Medicine, University of Ottawa, Ottawa, Ontario, Canada (Funnell); Department of Health Sciences, Carleton University, Ottawa, Ontario, Canada (Jull); Faculty of Health Sciences, School of Rehabilitation Therapy, Queen's University, Kingston, Ontario, Canada (Jull).

Author Contributions: Mr Dewidar had full access to all of the data in the study and takes responsibility for the integrity of the data and the accuracy of the data analysis. Drs Funnell, Jull, Mbuagbaw, and Welch were cosenior authors.

Concept and design: Dewidar, Shamseer, Akl, Young, Nicholls, Kennedy, Hardy, Rizvi, Waddington, Shea, Nkangu, Tufte, Pottie, Cuervo, Juando-Prats, Sharp, Little, Kredo, Mahande, Chamberlain, von Elm, Bhutta, Tugwell, Wiysonge, Funnell, Jull, Mbuagbaw, Welch.

Acquisition, analysis, or interpretation of data: Dewidar, Shamseer, Melendez-Torres, Akl, Ramke, Wang, Oloyede, Marshall, Kennedy, Hardy, Rizvi, Ghogomu, Rader, Shea, Ellingwood, Wolfenden, Horsley, Pottie, Cuervo, Juando-Prats, Feng, Sharp, Owusu-Addo, Francis, Kredo, Mahande, Pantoja, Bhutta, Wiysonge, Jull, Mbuagbaw.

Drafting of the manuscript: Dewidar, Shamseer, Melendez-Torres, Ramke, Rader, Pottie, Mahande, Tugwell, Jull, Welch.

Critical review of the manuscript for important intellectual content: Dewidar, Shamseer, Melendez-Torres, Akl, Wang, Oloyede, Young, Nicholls, Marshall, Kennedy, Hardy, Rizvi, Ghogomu, Waddington, Shea, Nkangu, Ellingwood, Wolfenden, Tufte, Horsley, Pottie, Cuervo, Juando-Prats, Feng, Sharp, Little, Owusu-Addo, Francis, Kredo, Mahande, Chamberlain, Pantoja, von Elm, Bhutta, Wiysonge, Funnell, Jull, Mbuagbaw, Welch.

Statistical analysis: Dewidar, Melendez-Torres, Wang, Oloyede, Kennedy, Mahande, Jull.

Obtained funding: Shamseer, Nicholls, Nkangu, Juando-Prats, Sharp, Funnell, Jull, Mbuagbaw, Welch.

Downloaded from jamanetwork.com by guest on 07/17/2026

Administrative, technical, or material support: Ramke, Oloyede, Young, Nicholls, Kennedy, Hardy, Rizvi, Ghogomu, Waddington, Shea, Wolfenden, Horsley, Sharp, Owusu-Addo, Kredo, Wiysonge, Funnell, Jull, Welch.

Supervision: Melendez-Torres, Pottie, von Elm, Jull, Mbuagbaw, Welch.

Conflict of Interest Disclosures: Dr Funnell reported service as a board member on College of Family Physicians of Canada and National Circle of Indigenous Medical Education. Dr Cuervo reported uncompensated service as an advisor and editorial board member for journals; he is employed by the Pan American Health Organization (PAHO/WHO), which has covered travel incurred as part of his position (a copyright agreement was submitted through the corresponding author); he reported holding a retirement fund with British Medical Association and the United Nations Joint Staff Pension Fund, over which he has no control. Dr Melendez-Torres reported receiving support from the National Institute for Health and Care Research through a Senior Investigator Award outside the submitted work. Dr von Elm reported authorship of the original STROBE statement. Dr Pottie reported grants from the Public Health Agency of Canada and the Canadian Institutes of Health Research (CIHR), with involvement in multiple funded research projects related to COVID-19 and health equity outside the submitted work, with payments made to Western University; he received consulting fees from CSL Behring, an honoraria from Community Diabetes CME, and holds a pending patent related to health information-based communities and knowledge incentive systems; he reported holding leadership roles in The Canadian Collaboration for Immigrant and Refugee Health, Cochrane Canada, and MSF Canada, all of which are unpaid. Dr Sharp reported serving in an unpaid leadership role on the Scientific Advisory Board of the Endometriosis Association of Ireland. Dr Young reported receiving grants from the Fogarty International Center of the National Institutes of Health, the Research, Evidence, and Development Initiative (READ-It) project funded by UK Aid, and the GELA project funded by the European Union, all supporting research activities; she also participated in a data safety monitoring board for a study on health systems strengthening in South Africa. No other disclosures were reported.

Funding/Support: This work was supported by the Canadian Institutes of Health Research (CIHR), grant number 173269.

Role of the Funder/Sponsor: CIHR had no role in the design and conduct of the study; collection, management, analysis, and interpretation of the data; preparation, review, or approval of the manuscript; and decision to submit the manuscript for publication.

Disclaimer: The opinions expressed in this article are those of the authors and do not necessarily represent the views of the NIHR or the UK Department of Health and Social Care.

Data Sharing Statement: See the Supplement.

Additional Contributions: We would like to acknowledge the efforts of the following members of the STROBE-Equity team for their contribution to the conceptualization of the guidance, design of the empirical studies and participation in the consensus meetings: Alba Antequera, PhD; Marc Avey, PhD; Peter Craig, PhD; Alassane Dicko, PhD; Isabel Fortier, PhD; Regina Greer-Smith; Matire Harwood, PhD; Janet Hatcher-Roberts, PhD; Mwenya Kasonde, PhD; Alison Krentel, PhD; Elizabeth Kristjansson, PhD; Laurenz Langer, PhD; Daeria Lawson, PhD; Elizabeth Loder, PhD; Olivia Magwood, PhD; David Moher, PhD; Loveline Lum Niba; Stephen G. Noorduyn, MSc; Ekwaro Obuku, PhD; Patrick M. Okwen, PhD; Jennifer Petkovic, PhD; Mark Petticrew, PhD; Alison Riddle, PhD; Peter Tanuseputro, PhD; Harry Wang, MD; Laura Weeks, PhD; George Wells, PhD; Howard White, PhD; Charles S. Wiysonge, PhD; Dana Simpson, PhD.

Additional Information: STROBE-Equity is being simultaneously published in *The BMJ* and *JAMA Network Open.* The article is identical except for minor stylistic and spelling differences in keeping with each journal's style. Citations from any of the journals can be used when citing this article. We have established a knowledge translation working group and are working on strategies to disseminate the research to different interest-holders and populations.

REFERENCES

1. Porta M, Greenland S, Hernán M, dos Santos Silva I, Last JM. *A Dictionary of Epidemiology. Oxford Reference*. Oxford University Press; 2014. doi:10.1093/acref/9780199976720.001.0001

2. Jull J, Whitehead M, Petticrew M, et al. When is a randomised controlled trial health equity relevant? Development and validation of a conceptual framework. *BMJ Open*. 2017;7(9):e015815. doi:10.1136/bmjopen-2016-015815

3. O'Neill J, Tabish H, Welch V, et al. Applying an equity lens to interventions: using PROGRESS ensures consideration of socially stratifying factors to illuminate inequities in health. *J Clin Epidemiol*. 2014;67(1):56-64. doi:10.1016/j.jclinepi.2013.08.005

4. Karran EL, Cashin AG, Barker T, et al. Using PROGRESS-plus to identify current approaches to the collection and reporting of equity-relevant data: a scoping review. *J Clin Epidemiol*. 2023;163:70-78. doi:10.1016/j.jclinepi.2023.09.017

Downloaded from jamanetwork.com by guest on 07/17/2026

5. Crenshaw K. Mapping the margins: intersectionality, identity politics, and violence against women of color. *Stanford Law Rev*. 1991;43(6):1241-1299. doi:10.2307/1229039

6. McCann L, Johnson L, Gkiouleka A, Pearce H, Ford J. EQUALSS GUIDE Multiple: a novel framework for equity-focused evidence synthesis. *Public Health Pract (Oxf)*. 2025;9:100600. doi:10.1016/j.puhip.2025.100600

7. Luchenski S, Maguire N, Aldridge RW, et al. What works in inclusion health: overview of effective interventions for marginalised and excluded populations. *Lancet*. 2018;391(10117):266-280. doi:10.1016/S0140-6736(17)31959-1

8. Tweed EJ, Thomson RM, Lewer D, et al. Health of people experiencing co-occurring homelessness, imprisonment, substance use, sex work and/or severe mental illness in high-income countries: a systematic review and meta-analysis. *J Epidemiol Community Health*. 2021;75(10):1010-1018. doi:10.1136/jech-2020-215975

9. Zhang CX, Lewer D, Aldridge RW, et al. Small numbers, big impact: making a utilitarian case for the contribution of inclusion health to population health in England. *J Epidemiol Community Health*. 2023;77(12):816-820. doi:10.1136/jech-2023-220849

10. Centers for Disease Control and Prevention. Glossary terminology considerations. May 15, 2024. Accessed October 24, 2024. https://restoredcdc.org/www.cdc.gov/global-health-equity/php/publications/terminology-considerations-1.html

11. American Medical Association. Advancing health equity: a guide to language, narrative and concepts. Accessed October 24, 2024. https://www.ama-assn.org/about/ama-center-health-equity/advancing-health-equity-guide-language-narrative-and-concepts-0

12. Public Health Ontario. Health equity data. Updated July 5, 2024. Accessed February 10, 2025. https://www.publichealthontario.ca/en/Data-and-Analysis/Health-Equity

13. Patient-Centered Outcomes Research Institute. Engagement rubric for applicants. Updated June 6, 2016. Accessed February 10, 2025. https://www.pcori.org/sites/default/files/Engagement-Rubric.pdf

14. Petkovic J, Riddle A, Akl EA, et al. Protocol for the development of guidance for stakeholder engagement in health and healthcare guideline development and implementation. *Syst Rev*. 2020;9(1):21. doi:10.1186/s13643-020-1272-5

15. Akl EA, Khabsa J, Petkovic J, et al. "Interest-holders": a new term to replace "stakeholders" in the context of health research and policy. *Cochrane Evid Synth Methods*. 2024;2(11):e70007. doi:10.1002/cesm.70007

16. Canadian Institutes of Health Research. Knowledge user engagement. Accessed September 10, 2023. https://cihr-irsc.gc.ca/e/49505.html

17. von Elm E, Altman DG, Egger M, Pocock SJ, Gøtzsche PC, Vandenbroucke JP; STROBE Initiative. Strengthening the Reporting of Observational Studies in Epidemiology (STROBE) statement: guidelines for reporting observational studies. *BMJ*. 2007;335(7624):806-808. doi:10.1136/bmj.39335.541782.AD

18. Antequera A, Lawson DO, Noorduyn SG, et al. Improving social justice in COVID-19 health research: interim guidelines for reporting health equity in observational studies. *Int J Environ Res Public Health*. 2021;18(17):9357. doi:10.3390/ijerph18179357

19. World Health Organization. Health equity. Accessed August 3, 2023. https://www.who.int/health-topics/health-equity#tab=tab_1

20. Khazanchi R, Evans CT, Marcelin JR. Racism, not race, drives inequity across the COVID-19 continuum. *JAMA Netw Open*. 2020;3(9):e2019933. doi:10.1001/jamanetworkopen.2020.19933

21. Pagel C. There is a real danger that COVID-19 will become entrenched as a disease of poverty. *BMJ*. 2021;373(986):n986. doi:10.1136/bmj.n986

22. Marmot M, Bell R. The sustainable development goals and health equity. *Epidemiology*. 2018;29(1):5-7. doi:10.1097/EDE.0000000000000773

23. Whitehead M, Dahlgren G; World Health Organization. *Concepts and Principles for Tackling Social Inequities in Health: Leveling Up Part 1*. WHO Regional Office for Europe; 2006.

24. Raphael D. *Social Determinants of Health*. 3rd ed. Canadian Scholars; 2016.

25. Pan American Health Organization. Policy on Research for Health. Technical report No. CD49/10. July 10, 2009. Accessed June 18, 2025. https://iris.paho.org/handle/10665.2/54411

26. Smith M, Hosking J, Woodward A, et al. Systematic literature review of built environment effects on physical activity and active transport—an update and new findings on health equity. *Int J Behav Nutr Phys Act*. 2017;14(1):158. doi:10.1186/s12966-017-0613-9

Downloaded from jamanetwork.com by guest on 07/17/2026

27. Daskalopoulou C, Stubbs B, Kralj C, Koukounari A, Prince M, Prina AM. Physical activity and healthy ageing: a systematic review and meta-analysis of longitudinal cohort studies. *Ageing Res Rev*. 2017;38:6-17. doi:10.1016/j.arr.2017.06.003

28. Freeman S, Bishop K, Spirgiene L, et al. Factors affecting residents transition from long term care facilities to the community: a scoping review. *BMC Health Serv Res*. 2017;17(1):689. doi:10.1186/s12913-017-2571-y

29. Dewidar O, Podinic I, Barbeau V, et al. Integrating sex and gender in studies of cardiac resynchronization therapy: a systematic review. *ESC Heart Fail*. 2022;9(1):420-427. doi:10.1002/ehf2.13733

30. Dewidar O, Al-Zubaidi A, Bondok M, et al. Reporting of equity in observational epidemiology: a methodological review. *J Glob Health*. 2024;14:04046. doi:10.7189/jogh.14.04046

31. Barker LC, Hussain-Shamsy N, Rajendra KL, et al. The use of key social determinants of health variables in psychiatric research using routinely collected health data: a systematic analysis. *Soc Psychiatry Psychiatr Epidemiol*. 2023;58(2):183-191. doi:10.1007/s00127-022-02368-x

32. Glover RE, van Schalkwyk MCI, Akl EA, et al. A framework for identifying and mitigating the equity harms of COVID-19 policy interventions. *J Clin Epidemiol*. 2020;128:35-48. doi:10.1016/j.jclinepi.2020.06.004

33. Tenforde MW, Fisher KA, Patel MM. Identifying COVID-19 risk through observational studies to inform control measures. *JAMA*. 2021;325(14):1464-1465. doi:10.1001/jama.2021.1995

34. Pan American Health Organization. Guidance for implementing non pharmacological public health measures in populations in situations of vulnerability in the context of COVID-19. 2020. Accessed March 29, 2022. https://iris.paho.org/handle/10665.2/53078

35. Brownson RC, Kumanyika SK, Kreuter MW, Haire-Joshu D. Implementation science should give higher priority to health equity. *Implement Sci*. 2021;16(1):28. doi:10.1186/s13012-021-01097-0

36. Welch VA, Norheim OF, Jull J, Cookson R, Sommerfelt H, Tugwell P; CONSORT-Equity and Boston Equity Symposium. CONSORT-Equity 2017 extension and elaboration for better reporting of health equity in randomised trials. *BMJ*. 2017;359:j5085. doi:10.1136/bmj.j5085

37. Welch V, Petticrew M, Tugwell P, et al; PRISMA-Equity Bellagio group. PRISMA-Equity 2012 extension: reporting guidelines for systematic reviews with a focus on health equity. *PLoS Med*. 2012;9(10):e1001333. doi:10.1371/journal.pmed.1001333

38. Welch VA, Akl EA, Guyatt G, et al. GRADE equity guidelines 1: considering health equity in GRADE guideline development: introduction and rationale. *J Clin Epidemiol*. 2017;90:59-67. doi:10.1016/j.jclinepi.2017.01.014

39. Moser KA, Leon DA, Gwatkin DR. How does progress towards the child mortality millennium development goal affect inequalities between the poorest and least poor? Analysis of Demographic and Health Survey data. *BMJ*. 2005;331(7526):1180-1182. doi:10.1136/bmj.38659.588125.79

40. Reidpath DD, Morel CM, Mecaskey JW, Allotey P. The Millennium Development Goals fail poor children: the case for equity-adjusted measures. *PLoS Med*. 2009;6(4):e1000062. doi:10.1371/journal.pmed.1000062

41. Social Sciences and Humanities Research Council; Canadian Institutes of Health Research; Natural Sciences and Engineering Research Council; Canada Foundation for Innovation. Capitalizing on big data: toward a policy framework for advancing digital scholarship in Canada. October 16, 2013. Accessed February 27, 2021. https://www.sshrc-crsh.gc.ca/about-au_sujet/publications/digital_scholarship_consultation_e.pdf

42. Government of India. Big data initiative. Accessed February 27, 2021. https://dst.gov.in/big-data-initiative

43. Cuervo LG, Martínez-Herrera E, Cuervo D, Jaramillo C. Improving equity using dynamic geographic accessibility data for urban health services planning. *Gac Sanit*. 2022;36(6):497-499. doi:10.1016/j.gaceta.2022.05.001

44. National Institute of Standards and Technology. The federal big data research and development strategic plan. Accessed February 27, 2021. https://bigdatawg.nist.gov/pdf/bigdatardstrategicplan.pdf

45. UK Research and Innovation. The UK's research and innovation infrastructure: opportunities to grow our capability. Accessed February 28, 2021. https://www.ukri.org/wp-content/uploads/2020/10/UKRI-201020-UKinfrastructure-opportunities-to-grow-our-capacity-FINAL.pdf

46. World Health Organization. Making fair choices on the path to universal health coverage: final report of the WHO consultative group on equity and universal health coverage. April 30, 2014. Accessed February 28, 2021. https://www.who.int/publications/i/item/9789241507158

47. Whitmee S, Green R, Belesova K, et al. Pathways to a healthy net-zero future: report of the Lancet Pathfinder Commission. *Lancet*. 2024;403(10421):67-110. doi:10.1016/S0140-6736(23)02466-2

48. Schlussel MM, Sharp MK, de Beyer JA, et al. Reporting guidelines used varying methodology to develop recommendations. *J Clin Epidemiol*. 2023;159:246-256. doi:10.1016/j.jclinepi.2023.03.018

Downloaded from jamanetwork.com by guest on 07/17/2026

49. Turner L, Shamseer L, Altman DG, Schulz KF, Moher D. Does use of the CONSORT Statement impact the completeness of reporting of randomised controlled trials published in medical journals? A Cochrane review. *Syst Rev*. 2012;1(1):60. doi:10.1186/2046-4053-1-60

50. Stevens A, Shamseer L, Weinstein E, et al. Relation of completeness of reporting of health research to journals' endorsement of reporting guidelines: systematic review. *BMJ*. 2014;348:g3804. doi:10.1136/bmj.g3804

51. Simera I, Moher D, Hirst A, Hoey J, Schulz KF, Altman DG. Transparent and accurate reporting increases reliability, utility, and impact of your research: reporting guidelines and the EQUATOR Network. *BMC Med*. 2010;8(1):24. doi:10.1186/1741-7015-8-24

52. Benchimol EI, Smeeth L, Guttmann A, et al; RECORD Working Committee. The REporting of studies Conducted using Observational Routinely-collected health Data (RECORD) statement. *PLoS Med*. 2015;12(10):e1001885. doi:10.1371/journal.pmed.1001885

53. Tong A, Sainsbury P, Craig J. Consolidated criteria for reporting qualitative research (COREQ): a 32-item checklist for interviews and focus groups. *Int J Qual Health Care*. 2007;19(6):349-357. doi:10.1093/intqhc/mzm042

54. O'Brien BC, Harris IB, Beckman TJ, Reed DA, Cook DA. Standards for reporting qualitative research: a synthesis of recommendations. *Acad Med*. 2014;89(9):1245-1251. doi:10.1097/ACM.0000000000000388

55. Funnell S, Jull J, Mbuagbaw L, et al. Improving social justice in observational studies: protocol for the development of a global and Indigenous STROBE-equity reporting guideline. *Int J Equity Health*. 2023;22(1):55. doi:10.1186/s12939-023-01854-1

56. Moher D, Schulz KF, Simera I, Altman DG. Guidance for developers of health research reporting guidelines. *PLoS Med*. 2010;7(2):e1000217. doi:10.1371/journal.pmed.1000217

57. Staniszewska S, Brett J, Simera I, et al. GRIPP2 reporting checklists: tools to improve reporting of patient and public involvement in research. *BMJ*. 2017;358:j3453. doi:10.1136/bmj.j3453

58. Gattrell WT, Logullo P, van Zuuren EJ, et al. ACCORD (ACcurate COnsensus Reporting Document): A reporting guideline for consensus methods in biomedicine developed via a modified Delphi. *PLoS Med*. 2024;21(1):e1004326. doi:10.1371/journal.pmed.1004326

59. United Nations General Assembly. United Nations Declaration on the Rights of Indigenous Peoples A/RES/61/295. October 2, 2007. Accessed September 10, 2019. https://docs.un.org/en/A/RES/61/295

60. Wang X, Dewidar O, Rizvi A, et al. A scoping review establishes need for consensus guidance on reporting health equity in observational studies. *J Clin Epidemiol*. 2023;160:126-140. doi:10.1016/j.jclinepi.2023.06.009

61. Rizvi A, Lawson DO, Young T, et al. Guidance relevant to the reporting of health equity in observational research: a scoping review protocol. *BMJ Open*. 2022;12(5):e056875. doi:10.1136/bmjopen-2021-056875

62. Dewidar O, Rader T, Waddington H, et al. Reporting of health equity considerations in equity-relevant observational studies: Protocol for a systematic assessment [version 1; peer review: awaiting peer review]. *F1000 Res*. 2022;615(11). doi:10.12688/f1000research.122185.1

63. Staniszewska S, Brett J, Simera I, et al. GRIPP2 reporting checklists: tools to improve reporting of patient and public involvement in research. *BMJ*. 2017;358:j3453. doi:10.1136/bmj.j3453

64. Caraballo C, Ndumele CD, Roy B, et al. Trends in racial and ethnic disparities in barriers to timely medical care among adults in the US, 1999 to 2018. *JAMA Health Forum*. 2022;3(10):e223856. doi:10.1001/jamahealthforum.2022.3856

65. Zimmerman FJ, Anderson NW. Trends in health equity in the United States by race/ethnicity, sex, and income, 1993-2017. *JAMA Netw Open*. 2019;2(6):e196386. doi:10.1001/jamanetworkopen.2019.6386

66. Bennett NC, Norman P, Albani V, Kingston A, Bambra C. The impact of the English national health inequalities strategy on inequalities in mortality at age 65: a time-trend analysis. *Eur J Public Health*. 2024;34(4):660-665. doi:10.1093/eurpub/ckae081

67. Holmberg V, Salmi H, Kattainen S, et al. Association between first language and SARS-CoV-2 infection rates, hospitalization, intensive care admissions and death in Finland: a population-based observational cohort study. *Clin Microbiol Infect*. 2022;28(1):107-113. doi:10.1016/j.cmi.2021.08.022

68. Yang K, Yang X, Jin C, et al. Global burden of type 1 diabetes in adults aged 65 years and older, 1990-2019: population based study. *BMJ*. 2024;385:e078432. doi:10.1136/bmj-2023-078432

69. Petkovic J, Jull J, Yoganathan M, et al. Reporting of health equity considerations in cluster and individually randomized trials. *Trials*. 2020;21(1):308. doi:10.1186/s13063-020-4223-5

Downloaded from jamanetwork.com by guest on 07/17/2026

70. Hosking J, Macmillan A, Jones R, Ameratunga S, Woodward A. Searching for health equity: validation of a search filter for ethnic and socioeconomic inequalities in transport. *Syst Rev*. 2019;8(1):94. doi:10.1186/s13643-019-1009-5

71. Prady SL, Uphoff EP, Power M, Golder S. Development and validation of a search filter to identify equity-focused studies: reducing the number needed to screen. *BMC Med Res Methodol*. 2018;18(1):106. doi:10.1186/s12874-018-0567-x

72. InterTASC Information Specialists' Sub-Group. The ISSG Search Filter Resource. Updated July 9, 2025. Accessed September 10, 2023. https://sites.google.com/a/york.ac.uk/issg-search-filters-resource/home

73. Lefebvre C, Glanville J, Briscoe S, et al. *Searching for and Selecting Studies*. Cochrane Handbook for Systematic Reviews of Interventions; 2019:67-107.

74. Hardeman A, Wong T, Denson JL, Postelnicu R, Rojas JC. Evaluation of health equity in COVID-19 vaccine distribution plans in the United States. *JAMA Netw Open*. 2021;4(7):e2115653. doi:10.1001/jamanetworkopen.2021.15653

75. Sung H, Nisotel L, Sedeta E, Islami F, Jemal A. Racial and ethnic disparities in survival among people with second primary cancer in the US. *JAMA Netw Open*. 2023;6(8):e2327429. doi:10.1001/jamanetworkopen.2023.27429

76. Conrad N, Molenberghs G, Verbeke G, et al. Trends in cardiovascular disease incidence among 22 million people in the UK over 20 years: population based study. *BMJ*. 2024;385:e078523. doi:10.1136/bmj-2023-078523

77. van Roode T, Pauly BM, Marcellus L, et al. Values are not enough: qualitative study identifying critical elements for prioritization of health equity in health systems. *Int J Equity Health*. 2020;19(1):162. doi:10.1186/s12939-020-01276-3

78. Dewidar O, McHale G, Al Zubaidi A, et al. Motivations for investigating health inequities in observational epidemiology: a content analysis of 320 studies. *J Clin Epidemiol*. 2024;168:111283. doi:10.1016/j.jclinepi.2024.111283

79. Kwan BM, Brownson RC, Glasgow RE, Morrato EH, Luke DA. Designing for dissemination and sustainability to promote equitable impacts on health. *Annu Rev Public Health*. 2022;43:331-353. doi:10.1146/annurev-publhealth-052220-112457

80. Bailey ZD, Krieger N, Agénor M, Graves J, Linos N, Bassett MT. Structural racism and health inequities in the USA: evidence and interventions. *Lancet*. 2017;389(10077):1453-1463. doi:10.1016/S0140-6736(17)30569-X

81. Plamondon K, Banner D, Cary MA, et al. Relational practices for meaningful inclusion in health research: Results of a deliberative dialogue study. *Health Expect*. 2024;27(1):e13865. doi:10.1111/hex.13865

82. Bauer GR. Incorporating intersectionality theory into population health research methodology: challenges and the potential to advance health equity. *Soc Sci Med*. 2014;110:10-17. doi:10.1016/j.socscimed.2014.03.022

83. Arcaya MC, Arcaya AL, Subramanian SV. Inequalities in health: definitions, concepts, and theories. *Glob Health Action*. 2015;8:27106. doi:10.3402/gha.v8.27106

84. Newman L, Baum F, Javanparast S, O'Rourke K, Carlon L. Addressing social determinants of health inequities through settings: a rapid review. *Health Promot Int*. 2015;30(suppl 2):ii126-ii143. doi:10.1093/heapro/dav054

85. Smith JD, Li DH, Rafferty MR. The Implementation Research Logic Model: a method for planning, executing, reporting, and synthesizing implementation projects. *Implement Sci*. 2020;15(1):84. doi:10.1186/s13012-020-01041-8

86. Coughlin SS, Mann P, Vernon M, et al. A logic framework for evaluating social determinants of health interventions in primary care. *J Hosp Manag Health Policy*. 2019;3:23. doi:10.21037/jhmhp.2019.09.03

87. Jenkins EK, McAuliffe C, Hirani S, et al. A portrait of the early and differential mental health impacts of the COVID-19 pandemic in Canada: findings from the first wave of a nationally representative cross-sectional survey. *Prev Med*. 2021;145:106333. doi:10.1016/j.ypmed.2020.106333

88. Kennedy M, Longbottom H, Mersha A, et al. Which way? Indigenous-led smoking cessation care: knowledge, attitudes and practices of Aboriginal and Torres Strait Islander health workers and practitioners—a national cross-sectional survey. *Nicotine Tob Res*. 2023;25(4):788-795. doi:10.1093/ntr/ntac256

89. Effect of Risk Mitigation Guidance for opioid and stimulant dispensations on mortality and acute care visits during dual public health emergencies: retrospective cohort study. *BMJ*. 2024;384:q615. doi:10.1136/bmj.q615

90. Kearns RJ, Kyzayeva A, Halliday LOE, Lawlor DA, Shaw M, Nelson SM. Epidural analgesia during labour and severe maternal morbidity: population based study. *BMJ*. 2024;385:e077190. doi:10.1136/bmj-2023-077190

91. Charlton JI. *Nothing About Us Without Us Disability Oppression and Empowerment*. University of California Press; 1998.

Downloaded from jamanetwork.com by guest on 07/17/2026

92. Jull J, King A, King M, et al. A principled approach to research conducted with Inuit, Métis, and First Nations people: promoting engagement inspired by the CIHR guidelines for health research involving aboriginal people (2007-2010). *Int Indig Policy J*. 2020;11(2):1-30. doi:10.18584/iipj.2020.11.2.10635

93. Marshall Z. Navigating risks and professional roles: research with lesbian, gay, bisexual, trans, and queer young people with intellectual disabilities. *J Empir Res Hum Res Ethics*. 2012;7(4):20-33. doi:10.1525/jer.2012.7.4.20

94. Marshall Z, Kaposy C, Brunger F, Welch V. Trans research ethics: challenges and recommendations for change. *Bull Appl Transgend Stud*. 2022;1(3-4):187-210. doi:10.57814/2rv3-kf42

95. Brisbois B, Plamondon K. The possible worlds of global health research: an ethics-focused discourse analysis. *Soc Sci Med*. 2018;196:142-149. doi:10.1016/j.socscimed.2017.11.034

96. Canadian Institutes of Health Research. Best practices in equity, diversity and inclusion in research practice and design. Updated June 6, 2025. Accessed September 20, 2024. https://sshrc-crsh.canada.ca/funding-financement/nfrf-fnfr/edi-eng.aspx

97. Randolph I, Simonds R, Sharps D, et al. Integration of individuals with lived experience to improve recruitment within criminal justice research: 'experience as the best teacher.' *Int J Soc Res Methodol*. 2023;28(1):1-14. doi:10.1080/13645579.2023.2276960

98. National Academies of Sciences, Engineering, and Medicine. Barriers to representation of underrepresented and excluded populations in clinical research. In: Bibbins-Domingo K, Helman A, eds. *Improving Representation in Clinical Trials and Research: Building Research Equity for Women and Underrepresented Groups*. National Academies Press; 2022.

99. Fisher J, Fones G, Arivalagan Y, Ahmadpour I, Akselrod S, Olsen M. WHO framework on meaningful engagement: a transformational approach to integrate lived experience in the noncommunicable disease and mental health agenda. *PLOS Glob Public Health*. 2024;4(5):e0002312. doi:10.1371/journal.pgph.0002312

100. Reopell L, Nolan TS, Gray DM II, et al. Community engagement and clinical trial diversity: navigating barriers and co-designing solutions-a report from the "Health Equity through Diversity" seminar series. *PLoS One*. 2023;18(2):e0281940. doi:10.1371/journal.pone.0281940

101. Shayo EH, Norheim OF, Mboera LEG, et al. Challenges to fair decision-making processes in the context of health care services: a qualitative assessment from Tanzania. *Int J Equity Health*. 2012;11(1):30. doi:10.1186/1475-9276-11-30

102. Black A, Strain K, Wallsworth C, et al. What constitutes meaningful engagement for patients and families as partners on research teams? *J Health Serv Res Policy*. 2018;23(3):158-167. doi:10.1177/1355819618762960

103. Woodward EN, Castillo AIM, True G, Willging C, Kirchner JE. Challenges and promising solutions to engaging patients in healthcare implementation in the United States: an environmental scan. *BMC Health Serv Res*. 2024;24(1):29. doi:10.1186/s12913-023-10315-y

104. Bellows M, Kovacs Burns K, Jackson K, Surgeoner B, Gallivan J. Meaningful and effective patient engagement: what matters most to stakeholders. *Patient Exp J*. 2015;2(1):18-28. doi:10.35680/2372-0247.1069

105. Richards DP, Poirier S, Mohabir V, Proulx L, Robins S, Smith J. Reflections on patient engagement by patient partners: how it can go wrong. *Res Involv Engagem*. 2023;9(1):41. doi:10.1186/s40900-023-00454-1

106. Easley J, Wassersug R, Matthias S, et al. Patient engagement in health research: perspectives from patient participants. *Curr Oncol*. 2023;30(3):2770-2780. doi:10.3390/curroncol30030210

107. Hawke LD, Sheikhan NY, Roberts S, McKee S. Research evidence and implementation gaps in the engagement of people with lived experience in mental health and substance use research: a scoping review. *Res Involv Engagem*. 2023;9(1):32. doi:10.1186/s40900-023-00442-5

108. Marsden N, Star L, Smylie J. Nothing about us without us in writing: aligning the editorial policies of the Canadian Journal of Public Health with the inherent rights of Indigenous Peoples. *Can J Public Health*. 2020;111(6):822-825. doi:10.17269/s41997-020-00452-w

109. Smylie J, Marsden N, Star L, et al. Requirement for meaningful engagement of First Nations, Inuit, Métis, and Indigenous peoples in publications about them. *Can J Public Health*. 2020;111(6):826-830. doi:10.17269/s41997-020-00450-y

110. Kennedy M, Maddox R, Mersha AG, et al. Koori quit pack: a feasibility study of a multi-component mailout smoking cessation support for Aboriginal and Torres Strait Islander People: "I would recommend it to anybody. it's just so much easier.". *Nicotine Tob Res*. 2024;27(3):418-428. doi:10.1093/ntr/ntae106

111. Parsons SK, Fineberg IC, Lin M, Singer M, Tang M, Erban JK. Promoting high-quality cancer care and equity through disciplinary diversity in team composition. *J Oncol Pract*. 2016;12(11):1141-1147. doi:10.1200/JOP.2016.013920

Downloaded from jamanetwork.com by guest on 07/17/2026

112. Hattery AJ, Smith E, Magnuson S, et al. Diversity, equity, and inclusion in research teams: the good, the bad, and the ugly. *Race Justice*. 2022;12(3):505-530. doi:10.1177/21533687221087373

113. Dewidar O, Elmestekawy N, Welch V. Improving equity, diversity, and inclusion in academia. *Res Integr Peer Rev*. 2022;7(1):4. doi:10.1186/s41073-022-00123-z

114. Love HB, Stephens A, Fosdick BK, Tofany E, Fisher ER. The impact of gender diversity on scientific research teams: a need to broaden and accelerate future research. *Humanit Soc Sci Commun*. 2022;9(1):386. doi:10.1057/s41599-022-01389-w

115. Forsythe LP, Carman KL, Szydlowski V, et al. Patient engagement in research: early findings from the Patient-Centered Outcomes Research Institute. *Health Aff (Millwood)*. 2019;38(3):359-367. doi:10.1377/hlthaff.2018.05067

116. Sergeant A, Saha S, Lalwani A, et al. Diversity among health care leaders in Canada: a cross-sectional study of perceived gender and race. *CMAJ*. 2022;194(10):E371-E377. doi:10.1503/cmaj.211340

117. Steyn PS, Cordero JP, Nai D, et al. Impact of community and provider-driven social accountability interventions on contraceptive uptake in Ghana and Tanzania. *Int J Equity Health*. 2022;21(1):142. doi:10.1186/s12939-022-01736-y

118. Andress L, Hall T, Davis S, Levine J, Cripps K, Guinn D. Addressing power dynamics in community-engaged research partnerships. *J Patient Rep Outcomes*. 2020;4(1):24. doi:10.1186/s41687-020-00191-z

119. Sayani A, Cordeaux E, Wu K, et al. Using the Power Wheel as a transformative tool to promote equity through spaces and places of patient engagement. *BMJ Open*. 2024;14(3):e074277. doi:10.1136/bmjopen-2023-074277

120. Gopal DP, Chetty U, O'Donnell P, Gajria C, Blackadder-Weinstein J. Implicit bias in healthcare: clinical practice, research and decision making. *Future Healthc J*. 2021;8(1):40-48. doi:10.7861/fhj.2020-0233

121. Crooks J, Flemming K, Shulman C, Hudson B. Opportunities and challenges in involving people with lived experience of inclusion health as co-researchers in palliative and end of life research: a rapid review and thematic synthesis. *Res Involv Engagem*. 2023;9(1):25. doi:10.1186/s40900-023-00436-3

122. Dewidar O, Kawala BA, Antequera A, et al; COVID-END Equity Task Force. Methodological guidance for incorporating equity when informing rapid-policy and guideline development. *J Clin Epidemiol*. 2022;150:142-153. doi:10.1016/j.jclinepi.2022.07.007

123. Mbuagbaw L, Tharao W, Husbands W, et al. A/C study protocol: a cross-sectional study of HIV epidemiology among African, Caribbean and Black people in Ontario. *BMJ Open*. 2020;10(7):e036259. doi:10.1136/bmjopen-2019-036259

124. Scheim AI, Bauer GR, Bastos JL, Poteat T. Advancing intersectional discrimination measures for health disparities research: protocol for a bilingual mixed methods measurement study. *JMIR Res Protoc*. 2021;10(8):e30987. doi:10.2196/30987

125. Varma R, Paz SH, Azen SP, et al; Los Angeles Latino Eye Study Group. The Los Angeles Latino Eye Study: design, methods, and baseline data. *Ophthalmology*. 2004;111(6):1121-1131. doi:10.1016/j.ophtha.2004.02.001

126. Miller CJ, Burgess JF Jr, Fischer EP, et al. Practical application of opt-out recruitment methods in two health services research studies. *BMC Med Res Methodol*. 2017;17(1):57. doi:10.1186/s12874-017-0333-5

127. Garnett A, Northwood M. Recruitment of community-based samples: experiences and recommendations for optimizing success. *Can J Nurs Res*. 2022;54(2):101-111. doi:10.1177/08445621211060935

128. Blumenberg C, Menezes AMB, Gonçalves H, Assunção MCF, Wehrmeister FC, Barros AJD. How different online recruitment methods impact on recruitment rates for the web-based coortesnaweb project: a randomised trial. *BMC Med Res Methodol*. 2019;19(1):127. doi:10.1186/s12874-019-0767-z

129. Kelley K, Clark B, Brown V, Sitzia J. Good practice in the conduct and reporting of survey research. *Int J Qual Health Care*. 2003;15(3):261-266. doi:10.1093/intqhc/mzg031

130. Weinmann T, AlZahmi A, Schneck A, Mancera Charry JF, Fröschl G, Radon K. Population-based assessment of health, healthcare utilisation, and specific needs of Syrian migrants in Germany: what is the best sampling method? *BMC Med Res Methodol*. 2019;19(1):5. doi:10.1186/s12874-018-0652-1

131. Ellard-Gray A, Jeffrey NK, Choubak M, Crann SE. Finding the hidden participant: solutions for recruiting hidden, hard-to-reach, and vulnerable populations. *Int J Qual Methods*. Published online December 17, 2015. doi:10.1177/1609406915621420

132. UyBico SJ, Pavel S, Gross CP. Recruiting vulnerable populations into research: a systematic review of recruitment interventions. *J Gen Intern Med*. 2007;22(6):852-863. doi:10.1007/s11606-007-0126-3

133. Aglipay M, Wylie JL, Jolly AM. Health research among hard-to-reach people: six degrees of sampling. *CMAJ*. 2015;187(15):1145-1149. doi:10.1503/cmaj.141076

Downloaded from jamanetwork.com by guest on 07/17/2026

134. Bayes R, Druckman JN, Safarpour AC. Studying science inequities: how to use surveys to study diverse populations. *Ann Am Acad Pol Soc Sci*. 2022;700(1):220-233. doi:10.1177/00027162221093970

135. O'Brien JE, Brewer KB, Jones LM, Corkhum J, Rizo CF. Rigor and respect: recruitment strategies for engaging vulnerable populations in research. *J Interpers Violence*. 2022;37(17-18):NP17052-NP17072. doi:10.1177/08862605211023497

136. Alkhouli M, Nanjundappa A, Annie F, Bates MC, Bhatt DL. Sex differences in case fatality rate of COVID-19: insights from a multinational registry. *Mayo Clin Proc*. 2020;95(8):1613-1620. doi:10.1016/j.mayocp.2020.05.014

137. Buttery SC, Philip KEJ, Alghamdi SM, Williams PJ, Quint JK, Hopkinson NS. Reporting of data on participant ethnicity and socioeconomic status in high-impact medical journals: a targeted literature review. *BMJ Open*. 2022;12(8):e064276. doi:10.1136/bmjopen-2022-064276

138. Sterne JAC, Hernán MA, Reeves BC, et al. ROBINS-I: a tool for assessing risk of bias in non-randomised studies of interventions. *BMJ*. 2016;355:i4919. doi:10.1136/bmj.i4919

139. Adkins-Jackson PB, Chantarat T, Bailey ZD, Ponce NA. Measuring structural racism: a guide for epidemiologists and other health researchers. *Am J Epidemiol*. 2022;191(4):539-547. doi:10.1093/aje/kwab239

140. Braveman PA, Arkin E, Proctor D, Kauh T, Holm N. Systemic and structural racism: definitions, examples, health damages, and approaches to dismantling. *Health Aff (Millwood)*. 2022;41(2):171-178. doi:10.1377/hlthaff.2021.01394

141. Borrell LN, Elhawary JR, Fuentes-Afflick E, et al. Race and genetic ancestry in medicine—a time for reckoning with racism. *N Engl J Med*. 2021;384(5):474-480. doi:10.1056/NEJMms2029562

142. Coyer L, Boyd A, Schinkel J, et al. Differences in SARS-CoV-2 infections during the first and second wave of SARS-CoV-2 between six ethnic groups in Amsterdam, the Netherlands: a population-based longitudinal serological study. *Lancet Regional Health—Europe*. 2022;13:100284. doi:10.1016/j.lanepe.2021.100284

143. Choi A, Lee H, Jeong HE, et al. Association between exposure to antibiotics during pregnancy or early infancy and risk of autism spectrum disorder, intellectual disorder, language disorder, and epilepsy in children: population based cohort study. *BMJ*. 2024;385:e076885. doi:10.1136/bmj-2023-076885

144. Dewidar O, Ali A-Z, Bondok M, et al. Reporting of equity in observational epidemiology: a methodological review. *J Glob Health*. 2024;14. doi:10.7189/jogh.14.04046

145. Eckersley R. Beyond inequality: acknowledging the complexity of social determinants of health. *Soc Sci Med*. 2015;147:121-125. doi:10.1016/j.socscimed.2015.10.052

146. Groenwold RHH, Nelson DB, Nichol KL, Hoes AW, Hak E. Sensitivity analyses to estimate the potential impact of unmeasured confounding in causal research. *Int J Epidemiol*. 2010;39(1):107-117. doi:10.1093/ije/dyp332

147. Wang R, Lagakos SW, Ware JH, Hunter DJ, Drazen JM. Statistics in medicine–reporting of subgroup analyses in clinical trials. *N Engl J Med*. 2007;357(21):2189-2194. doi:10.1056/NEJMsr077003

148. Burke JF, Sussman JB, Kent DM, Hayward RA. Three simple rules to ensure reasonably credible subgroup analyses. *BMJ*. 2015;351:h5651. doi:10.1136/bmj.h5651

149. Altman DG, Bland JM. Interaction revisited: the difference between two estimates. *BMJ*. 2003;326(7382):219. doi:10.1136/bmj.326.7382.219

150. Harper S, King NB, Young ME. Impact of selective evidence presentation on judgments of health inequality trends: an experimental study. *PLoS One*. 2013;8(5):e63362. doi:10.1371/journal.pone.0063362

151. King NB, Harper S, Young ME. Use of relative and absolute effect measures in reporting health inequalities: structured review. *BMJ*. 2012;345:e5774. doi:10.1136/bmj.e5774

152. Scanlon JK, Wofford L, Fair A, Philippi D. Predictors of participation in clinical research. *Nurs Res*. 2021;70(4):289-297. doi:10.1097/NNR.0000000000000513

153. Aung YK, Swe PP, Kyaw Z, Thein ST. Differential loss to follow-up rates among adult tuberculosis patients—findings from the largest private sector tuberculosis clinic database in Myanmar. *PLoS One*. 2019;14(6):e0218450. doi:10.1371/journal.pone.0218450

154. Round R, Gokool N, Manica G, Paschall L, Foulcer S. Improving access for and experience of transgender and non-binary patients in clinical research: insights from a transgender patient focus group and targeted literature reviews. *Contemp Clin Trials*. 2023;131:107243. doi:10.1016/j.cct.2023.107243

155. Walker LE, Stackpoole M, Hodge D, FitzGerald R. Incorporating transgender and nonbinary participants in phase 1 clinical drug trials: current knowledge gaps and considerations. *Br J Clin Pharmacol*. 2024;90(10):2343-2348. doi:10.1111/bcp.16073

156. Howe CJ, Cole SR, Lau B, Napravnik S, Eron JJ Jr. Selection bias due to loss to follow up in cohort studies. *Epidemiology*. 2016;27(1):91-97. doi:10.1097/EDE.0000000000000409

Downloaded from jamanetwork.com by guest on 07/17/2026

157. Ellen JG, Matos J, Viola M, et al. Participant flow diagrams for health equity in AI. *J Biomed Inform*. 2024;152:104631. doi:10.1016/j.jbi.2024.104631

158. Proto E, Quintana-Domeque C. COVID-19 and mental health deterioration by ethnicity and gender in the UK. *PLoS One*. 2021;16(1):e0244419. doi:10.1371/journal.pone.0244419

159. Vinter N, Cordsen P, Johnsen SP, et al. Temporal trends in lifetime risks of atrial fibrillation and its complications between 2000 and 2022: Danish, nationwide, population based cohort study. *BMJ*. 2024;385:e077209. doi:10.1136/bmj-2023-077209

160. Scott MM, Ménard A, Sun AH, et al. Building evidence to advance health equity: a systematic review on care-related outcomes for older, minoritised populations in long-term care homes. *Age Ageing*. 2024;53(4):afae059. doi:10.1093/ageing/afae059

161. Howe LD. Handbook on health inequality monitoring. *Int J Epidemiol*. 2014;43(4):1345-1346. doi:10.1093/ije/dyu124

162. Chen S, Li J, Shu M. Use of participation to prevalence ratio for evaluating the representation status of women in oncology clinical trials. *JAMA Oncol*. 2022;8(3):479-480. doi:10.1001/jamaoncol.2021.6968

163. Scott PE, Unger EF, Jenkins MR, et al. Participation of women in clinical trials supporting FDA approval of cardiovascular drugs. *J Am Coll Cardiol*. 2018;71(18):1960-1969. doi:10.1016/j.jacc.2018.02.070

164. Antequera A, Madrid-Pascual O, Solà I, et al. Female under-representation in sepsis studies: a bibliometric analysis of systematic reviews and guidelines. *J Clin Epidemiol*. 2020;126:26-36. doi:10.1016/j.jclinepi.2020.06.014

165. Jin X, Chandramouli C, Allocco B, Gong E, Lam CSP, Yan LL. Women's participation in cardiovascular clinical trials from 2010 to 2017. *Circulation*. 2020;141(7):540-548. doi:10.1161/CIRCULATIONAHA.119.043594

166. Carcel C, Harris K, Peters SAE, et al. Representation of women in stroke clinical trials: a review of 281 trials involving more than 500,000 participants. *Neurology*. 2021;97(18):e1768-e1774. doi:10.1212/WNL.0000000000012767

167. Althubaiti A. Information bias in health research: definition, pitfalls, and adjustment methods. *J Multidiscip Healthc*. 2016;9:211-217. doi:10.2147/JMDH.S104807

168. Anderson TS, Yeh RW, Herzig SJ, et al. Trends and disparities in ambulatory follow-up after cardiovascular hospitalizations: a retrospective cohort study. *Ann Intern Med*. 2024;177(9):1190-1198. doi:10.7326/M23-3475

169. Tsiampalis T, Panagiotakos DB. Missing-data analysis: socio- demographic, clinical and lifestyle determinants of low response rate on self- reported psychological and nutrition related multi- item instruments in the context of the ATTICA epidemiological study. *BMC Med Res Methodol*. 2020;20(1):148. doi:10.1186/s12874-020-01038-3

170. Civantos AM, Bertelli A, Gonçalves A, et al. Mental health among head and neck surgeons in Brazil during the COVID-19 pandemic: a national study. *Am J Otolaryngol*. 2020;41(6):102694. doi:10.1016/j.amjoto.2020.102694

171. Chen X, Chen H. Differences in preventive behaviors of COVID-19 between urban and rural residents: lessons learned from a cross-sectional study in China. *Int J Environ Res Public Health*. 2020;17(12):4437. doi:10.3390/ijerph17124437

172. Ling AY, Montez-Rath ME, Carita P, et al. An overview of current methods for real-world applications to generalize or transport clinical trial findings to target populations of interest. *Epidemiology*. 2023;34(5):627-636. doi:10.1097/EDE.0000000000001633

173. Schwartz AL, Alsan M, Morris AA, Halpern SD. Why diverse clinical trial participation matters. *N Engl J Med*. 2023;388(14):1252-1254. doi:10.1056/NEJMp2215609

174. Monroe SJ. Discussing inconvenient truths about the lack of generalizability of Alzheimer's research to minoritized populations. *Alzheimers Dement (N Y)*. 2023;9(3):e12406. doi:10.1002/trc2.12406

175. Wallace N, O'Keeffe S, Gardner H, Shiely F. Underrecording and underreporting of participant ethnicity in clinical trials is persistent and is a threat to inclusivity and generalizability. *J Clin Epidemiol*. 2023;162:81-89. doi:10.1016/j.jclinepi.2023.08.008

176. Hsu CY, Yang W, Parikh RV, et al; CRIC Study Investigators. Race, genetic ancestry, and estimating kidney function in CKD. *N Engl J Med*. 2021;385(19):1750-1760. doi:10.1056/NEJMoa2103753

177. Javed Z, Haisum Maqsood M, Yahya T, et al. Race, racism, and cardiovascular health: applying a social determinants of health framework to racial/ethnic disparities in cardiovascular disease. *Circ Cardiovasc Qual Outcomes*. 2022;15(1):e007917. doi:10.1161/CIRCOUTCOMES.121.007917

178. Marmot M. Social determinants of health inequalities. *Lancet*. 2005;365(9464):1099-1104. doi:10.1016/S0140-6736(05)71146-6

Downloaded from jamanetwork.com by guest on 07/17/2026

179. Dahlgren G, Whitehead M. The Dahlgren-Whitehead model of health determinants: 30 years on and still chasing rainbows. *Public Health*. 2021;199:20-24. doi:10.1016/j.puhe.2021.08.009

180. Burford BJ, Welch V, Waters E, et al. Testing the PRISMA-Equity 2012 reporting guideline: the perspectives of systematic review authors. *PLoS One*. 2013;8(10):e75122. doi:10.1371/journal.pone.0075122

181. Maden M. Consideration of health inequalities in systematic reviews: a mapping review of guidance. *Syst Rev*. 2016;5(1):202. doi:10.1186/s13643-016-0379-1

182. National Academies of Sciences, Engineering, and Medicine. The root causes of health inequity. In: Baciu A, Negussie Y, Geller A, et al, eds. *Communities in Action: Pathways to Health Equity*. National Academies Press; 2017.

183. Purnell TS, Fakunle DO, Bone LR, et al. Overcoming barriers to sustaining health equity interventions: insights from the National Institutes of Health Centers for Population Health and Health Disparities. *J Health Care Poor Underserved*. 2019;30(3):1212-1236. doi:10.1353/hpu.2019.0083

184. Buajitti E, Chiodo S, Rosella LC. Agreement between area- and individual-level income measures in a population-based cohort: implications for population health research. *SSM Popul Health*. 2020;10:100553. doi:10.1016/j.ssmph.2020.100553

SUPPLEMENT.

eAppendix 1. Reporting Guideline Checklists
eAppendix 2. STROBE-Equity Project Team Composition
eAppendix 3. Findings from the Key Informant Interviews
eAppendix 4. Global Online Survey Details
eAppendix 5. Survey Characteristics and Results
eAppendix 6. Discussion During Consensus Meeting Regarding the 8 Proposed Extension Items
eAppendix 7. Additional Details Regarding Survey and Consensus Methods

Downloaded from jamanetwork.com by guest on 07/17/2026
