# Supplementary material for: Mapping the Evolving AI Preferences and Care Needs in Orthopedic Transitional Care From Hospitals to Home: Cross-Sectional Study
Source: JMIR AI. 2026 Aug 4;5:e91061. doi: 10.2196/91061 (PMC13436801; doi:10.2196/91061)
Supplement: Supplementary file 3 [file ai-v5-e91061-s003.pdf]

RESEARCH METHODS

A Consensus-Based Checklist for Reporting of Survey Studies (CROSS)

Akash Sharma, MBBS[1,2], Nguyen Tran Minh Duc, MD[2,3], Tai Luu Lam Thang, MD[2,4], Nguyen Hai Nam, MD[2,5], Sze Jia Ng, MD[2,6], Kirellos Said Abbas, MBCH[2,7], Nguyen Tien Huy, MD, PhD[8], Ana Marušić, MD, PhD[9], Christine L. Paul, PhD[10], Janette Kwok, MBBS[11], Juntra Karbwang, MD, PhD[12], Chiara de Waure, MD, MSc, PhD[13], Frances J. Drummond, PhD[14], Yoshiyuki Kizawa, MD, PhD[15], Erik Taal, PhD[16], Joeri Vermeulen, MSN, CM[17,18], Gillian H. M. Lee, PhD[19], Adam Gyedu, MD, MPH[20], Kien Gia To, PhD[21], Martin L. Verra, PhD[22], Évelyne M. Jacqz-Aigrain, MD, PhD[23], Wouter K. G. Leclercq, MD[24], Simo T. Salminen, PhD[25], Cathy Donald Sherbourne, PhD[26], Barbara Mintzes, PhD[27], Sergi Lozano, PhD[28], Ulrich S. Tran, DSc[29], Mitsuaki Matsui, MD, MSc, PhD[12], and Mohammad Karamouzian, DVM, MSc, PhD candidate[30,31]

[1]University College of Medical Sciences and Guru Teg Bahadur Hospital, Dilshad Garden, Delhi, India; [2]Online Research Club, Nagasaki, Japan; [3]Faculty of Medicine, University of Medicine and Pharmacy, Ho Chi Minh City, Vietnam; [4]Department of Emergency, City's Children Hospital, Ho Chi Minh City, Vietnam; [5]Division of Hepato-Biliary-Pancreatic Surgery and Transplantation, Department of Surgery, Graduate School of Medicine, Kyoto University, Kyoto, Japan; [6]Department of Medicine, Crozer Chester Medical Center, Upland, PA, USA; [7]Faculty of Medicine, Alexandria University, Alexandria, Egypt; [8]Institute of Tropical Medicine (NEKKEN) and School of Tropical Medicine and Global Health, Nagasaki University, Nagasaki, Japan; [9]Department of Research in Biomedicine and Health, University of Split School of Medicine, Split, Croatia; [10]School of Medicine and Public Health, University of Newcastle, Callaghan, Australia; [11]Division of Transplantation and Immunogenetics, Department of Pathology, Queen Mary Hospital Hong Kong, Pok Fu Lam, Hong Kong; [12]School of Tropical Medicine and Global Health, Nagasaki University, Nagasaki, Japan; [13]Department of Medicine and Surgery, University of Perugia, Perugia, Italy; [14]Cancer Research at UCC, University College Cork, Cork, Ireland; [15]Department of Palliative Medicine, Kobe University School of Medicine, Hyogo, Japan; [16]Department of Psychology, Health & Technology, Faculty of Behavioural, Management and Social Sciences, University of Twente, Enschede, Netherlands; [17]Department of Public Health, Biostatistics and Medical Informatics Research Group, Vrije Universiteit Brussel (VUB), Brussels, Belgium; [18]Department of Health Care, Knowledge Centre Brussels Integrated Care, Erasmus Brussels University of Applied Sciences and Arts, Brussels, Belgium; [19]Paediatric Dentistry and Orthodontics, Faculty of Dentistry, University of Hong Kong, Pok Fu Lam, Hong Kong; [20]Department of Surgery, School of Medicine and Dentistry, Kwame Nkrumah University of Science and Technology, Kumasi, Ghana; [21]Faculty of Public Health, University of Medicine and Pharmacy, Ho Chi Minh City, Vietnam; [22]Department of Physiotherapy, Bern University Hospital, Insel Group, Bern, Switzerland; [23]Hopital Robert-Debre AP-HP, Clinical Investigation Center, Paris, France; [24]Department of Surgery, Máxima Medical Center, Veldhoven, Veldhoven, the Netherlands; [25]Department of Social Psychology, University of Helsinki, Helsinki, Finland; [26]RAND, Santa Monica, CA, USA; [27]School of Pharmacy and Charles Perkins Centrey, Faculty of Medicine and Health, The University of Sydney, Sydney, Australia; [28]School of Economics, University of Barcelona, Barcelona, Spain; [29]Department of Cognition, Emotion, and Methods in Psychology, School of Psychology, University of Vienna, Vienna, Austria; [30]School of Population and Public Health, University of British Columbia, Vancouver, BC, Canada; [31]HIV/STI Surveillance Research Center, and WHO Collaborating Center for HIV Surveillance, Institute for Futures Studies in Health, Kerman University of Medical Sciences, Kerman, Iran.

KEY WORDS: Checklist; Surveys and Questionnaires; Delphi technique.

J Gen Intern Med 36(10):3179–87
DOI: 10.1007/s11606-021-06737-1
© Society of General Internal Medicine 2021

INTRODUCTION

A survey is a list of questions aiming to extract a set of desired data or opinions from a particular group of people.[1] Surveys can be administered quicker than some other methods of data gathering and facilitate data collection from a large number of participants. Numerous questions can be included in a survey that allow for increased flexibility in evaluation of several research areas, such as analysis of risk factors, treatment outcomes, disease trends, cost-effectiveness of care, and quality of life. Surveys can be conducted by phone, mail, face-to-face, or online using web-based software and applications. Online surveys can help reduce or prevent geographical dependence and increase the validity, reliability, and statistical power of the studies. Moreover, online surveys facilitate rapid survey administration as well as data collection and analysis.[2]

Surveys are frequently used in a variety of research areas. For example, a PubMed search of the key word "survey" on

Akash Sharma and Minh Duc Nguyen Tran contributed equally to this work.

Received September 15, 2020
Accepted March 17, 2021
Published online April 22, 2021

January 7, 2021, generated over 1,519,000 results. These studies are used for a number of purposes, including but not limited to opinion polls, trend analyses, evaluation of policies, measuring the prevalence of diseases.[3–12] Although many surveys have been published in high-impact journals, comprehensive reporting guidelines for survey research are limited[13, 14] and substantial variabilities and inconsistencies can be identified in the reporting of survey studies. Indeed, different studies have presented multiform patterns of survey designs and reported results in various non-systematic ways.[15–17]

Evidence-based tools developed by experts could help streamline particular procedures that authors could follow to create reproducible and higher quality studies.[18–20] Research studies that have transparent and accurate reporting may be more reliable and could have a more significant impact on their potential audience.[19] However, that is often not the case when it comes to reporting research findings. For example, Moher et al.[20] reported that, although over 63,000 new studies are published in PubMed on a monthly basis, many publications face the problem of inadequate reporting. Given the lack of standardization and poor quality of reporting, the Enhancing the QUAlity and Transparency Of health Research (EQUATOR) Network was created to help researchers publish high-impact health research.[20] Several important guidelines for various types of research studies have been created and listed on the EQUATOR website, including but not limited to the Consolidated Standards of Reporting Trials and encompasses (CONSORT) for randomized control trial, Strengthening the Reporting of Observational studies in Epidemiology (STROBE) for observational studies, and Preferred Reporting Items for Systemic Reviews and Meta-analyses (PRISMA) for systematic reviews and meta-analyses. The introduction of PRISMA checklist in 2009 led to a substantial increase in the quality of the systemic reviews and is a good example of how poor reporting, biases, and unsatisfactory results can be significantly addressed by implementing and following a validated reporting guideline.[21]

SURGE[22] and CHERRIES[23] are frequently recommended for reporting of non-web and web-based surveys. However, a report by Tarek et al. found that many items of the SURGE and CHERRIES guidelines (e.g., development, description, testing of the questionnaire, advertisement, and administration of the questionnaire, sample representativeness, response rates, informed consent, statistical analysis) had been missed by authors. The authors therefore concluded a need to produce a single universal guideline as a standard quality-reporting tool for surveys. Moreover, these guidelines lack a structured approach for the development of guidelines. For example, CHERRIES which was developed in 2004 lacks a comprehensive literature review and the Delphi exercise. These steps are crucial in developing guidelines as they help identify potential gaps and opinions of different experts in the field.[20, 24] While the SURGE checklist used a literature review for generation of their items, it also lacks the Delphi exercise and is limited to only self-administered postal surveys. There is also little information available about the experts involved in the development of these checklists. SURGE's limited citations since its publication suggest that it is not commonly used by authors and not recommended by journals. Furthermore, even after the development of these guidelines (SURGE and CHERRIES), there has been limited improvement in reporting of surveys. For example, Alvin et al. reviewed 102 surveys in top nephrology journals and found that the quality of surveys was suboptimal and highlighted the need for new reporting guidelines to improve reporting quality and increase transparency.[25] Similarly, Prasad et al. found significant heterogeneity in reporting of radiology surveys published in major radiology journals and suggested the need for guidelines to increase the homogeneity and generalizability of survey results.[26] Mark et al. also found several deficiencies in survey methodologies and reporting practices and suggested a need for establishing minimum reporting standards for survey studies.[27] Similar concerns regarding the qualities of surveys have been raised in other medical fields.[28–33]

Because of concerns regarding survey qualities and lack of well-developed guidelines, there is a need for a single comprehensive tool that can be used as a standard reporting checklist for survey research to address significant discrepancies in the reporting of survey studies.[13, 25–28, 31, 32] The purpose of this study was to develop a universal checklist for both web- and non-web-based surveys. Firstly, we established a workgroup to search the literature for potential items that can be included in our checklist. Secondly, we collected information about experts in the field of survey research and emailed them an invitation letter. Lastly, we conducted three rounds of rating by the Delphi method.

METHODS

Our study was performed from January 2018 to December 2019 using the Delphi method. This method is encouraged for use in scientific research as a feasible and reliable approach to reach final consensus among experts.[34] The process of checklist development included five phases: (i) planning; (ii) drafting of checklist items; (iii) consensus building using the Delphi method; (iv) dissemination of guidelines; and (v) maintenance of guidelines.

Planning Phase

In the planning phase, we established a workgroup, secured resources, reviewed the existing reporting guidelines, and drafted the plan and timeline of our project. To facilitate the development of Checklist for Reporting of Survey Studies (CROSS), a reporting checklist workgroup was set up. This workgroup had seven members from five countries. The expert panel members were found via searching original survey-based studies published between January 2004 and December 2016. The experts were selected based on their number of high-

impact and highly cited publications using survey research methods. Furthermore, members of the EQUATOR Network and contributors to PRISMA checklist were involved. Panel members' information, such as current affiliation, email address, and number of survey studies involved in were collected through their ResearchGate profiles (see Supplement 1). Lastly, a list of potential panel members was created and an invitation letter was emailed to every expert to inquire about their interest in participating in our study. Consenting experts received a follow-up email with a detailed explanation of the research objectives and the Delphi approach.

Drafting the Checklist

This process generated a list of potential items that could be included in the checklist. This procedure included searching the literature for potential items to be considered for inclusion in the checklist, establishing a checklist based on those potential items, and revising the checklist. Firstly, we conducted a literature review to identify survey studies published in major medical journals and extracted relevant information for drafting our potential checklist items (see Supplement 2 for a sample search strategy). Secondly, we searched the EQUATOR Network for previously published checklists for reporting of survey studies. Thirdly, three teams of two researchers independently extracted the potential items that could be included in our checklist. Lastly, our group members worked together to revise the checklist and remove any duplicates (Fig. 1). We discussed the importance and relevance of each potential item and compared each of them to the selected literature.

Consensus Phase Using the Delphi Method

The first round of Delphi was conducted using SurveyMonkey (SurveyMonkey Inc., San Mateo, CA, USA; www.surveymonkey.com). An email was sent to the expert panel containing information about the Delphi process, the timeline of each Delphi phase, and a detailed overview of the project. A Likert scale was used for rating items from 1 (strongly disagree) to 5 (strongly agree). Experts were also encouraged to provide their comments, modify items, or propose a new item that they felt was necessary to be included in the checklist. Nonresponding experts were sent weekly follow-up reminders. Items that did not reach consensus were rerated in the second round along with the modified or newly added items. The main objectives of the first round were to determine unnecessary items and identify incomplete items in the survey checklist. A pre-set 70% agreement (70% experts rating 4/5 or 5/5) was used as a cutoff for including an item in the final checklist.[35] Items that did not reach the 70% agreement threshold were adjusted according to experts' feedback and redistributed to the panelists for round 2. In the second round, we included items that did not reach consensus in round one. In this round, experts were also provided with their round one scoring so that they could modify or preserve their previous responses. Lastly, a third round of Delphi was launched to solve any disagreements about the inclusion of items that did not reach consensus in the second round.

RESULTS

A total of 24 experts with a median (Q1, Q3) of 20 (15.75, 31) years of research experience participated in our study. Overall, 24 items were selected in their original form in the first round, and 27 items were reviewed in the second round. Out of these 27 items, 10 items were merged into five, and 11 items were modified based on experts' comments. In the second round, 24 experts participated and 18 items were finally included. Overall, 18 experts responded in the third round and only one additional item was included in this round.

All details regarding the percentage agreement and mean and standard deviation (SD) of items included in the checklist are presented in Table 1. CROSS contains 19 sections with 40 different items, including "Title and abstract" (section 1); "Introduction" (sections 2 and 3); "Methods" (sections 4–10); "Results" (sections 11–13); "Discussion" (sections 14–16); and other items (sections 17–19). Please see Supplement 3 for the final checklist.

DISCUSSION

The development of CROSS is the result of a literature review and Delphi process which involved international experts with significant expertise in the development and implementation of survey studies. CROSS includes both evidenced-informed and expert consensus-based items which are intended to serve as a tool that helps improve the quality of survey studies.

The detailed descriptions of the methods and procedures in developing this guideline are provided in this paper so that the quality of the checklist can be assessed by other scholars. Our Delphi respondent members were made up of a panel of experts with backgrounds in different disciplines. We also spent a considerable amount of time researching and debating the potential items to be included in our checklist. During the Delphi process, the agreement of each potential item was rated by participants according to a 5-point Likert scale. Although the entire process was conducted electronically, we gathered data and feedback from the participants via email instead of conducting Skype or face-to-face discussions as suggested by the EQUATOR network.[13]

In comparison to the CHERRIES or SURGE checklists, CROSS provides a single but comprehensive tool which is organized according to the typical primary sections required for peer-reviewed publications. It also assists researchers in developing a comprehensive research protocol prior to conducting a survey. The "Introduction" provides a clear overview of the aim of the survey. In the "Methods" section, our checklist provides a detailed explanation of initiating and developing the survey, including study design, data collection methods, sample size

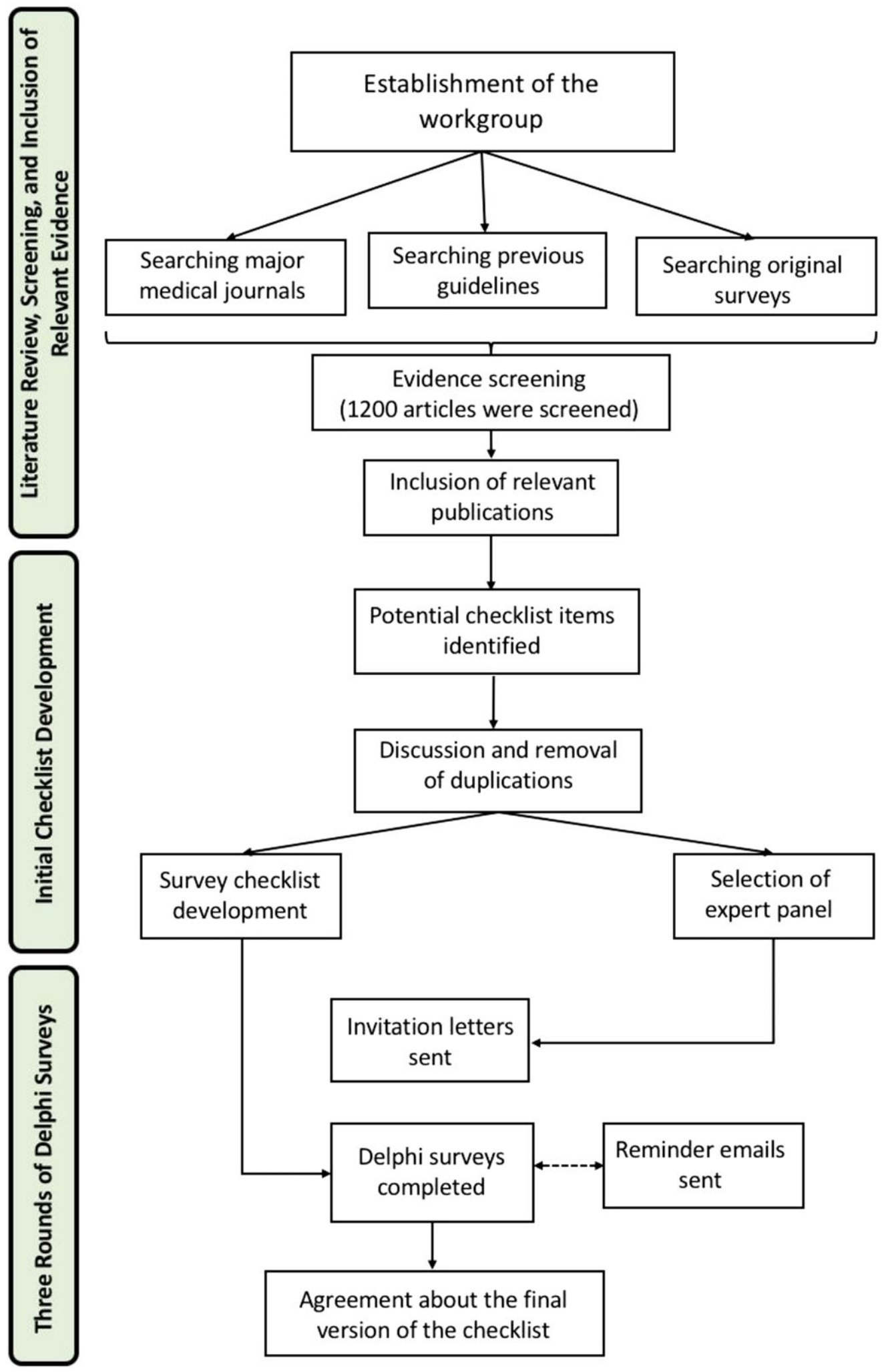

Fig. 1 Different stages of developing the checklist.

calculation, survey administration, study preparation, ethical considerations, and statistical analysis. The “Results” section of CROSS describes the respondent characteristics followed by the descriptive and main results, issues that are not discussed in CHERRIES and SURGE checklists. Also, our checklist can be used in both non-web-based and web-based surveys that serves all types of survey-based studies. New items were added to our checklist to address the gaps in the available tools. For example, in item 10b, we included reports of any modification of variables. This can help researchers to justify and readers to understand why there was a need to modify the variables. In item 11b, we encourage researchers to state the reasons for non-participation at each stage. Publishing these reasons can be useful for future researchers intending to conduct a similar survey. Finally, we have added components related to limitations, interpretation, and generalizability of study results to the “Discussion” section, which are an important effort in increasing transparency and external validity. These components are missing from previous checklists (i.e., CHERRIES and SURGE).

Dissemination and Maintenance of the Checklist

Following the consensus phase, we will publish our checklist statement together with a detailed Explanation and Elaboration (E&E) document in which an in-depth explanation of the scientific rationale for each recommendation will be provided. To disseminate our final checklist widely, we aim to promote it in various journals, make it easily available on multiple websites including EQUATOR, and disseminate it through presentations at relevant conferences if necessary. We will also use social media to reach certain demographics, and also the key persons in research organizations who are regularly conducting surveys in different specialties. We also aim to seek endorsement of CROSS by journal editors, professional societies, and researchers, and to collect feedback from scholars about their experience.

Taking comments, critics, and suggestion from experts for revising and correcting our guidelines could help maintain the relevancy of the checklist. Lastly, we are planning on publishing CROSS in several non-English languages to increase its accessibility across the scientific community.

Table 1 Percentage Agreement and Mean Score with Standard Deviation of the Items in Different Rounds

Section/topic	Item	Item description	Item included after which round of Delphi (round 1/round 2/round 3)	Agreement in round 1 (%) Mean score* ± standard deviation	Agreement in round 2 (%) Mean score ± standard deviation	Agreement in round 3 (%) Mean score ± standard deviation
Title and abstract						
Title and abstract	1a	State the word "survey" along with a commonly used term in title or abstract to introduce the study's design.	Round 1	86.3%<br>4.23 ± 0.69	-	-
	1b	Provide an informative summary in the abstract, covering background, objectives, methods, findings/results, interpretation/discussion, and conclusions.	Round 2	95.6%<br>4.70 ± 0.56	95.4%	-
Introduction						
Background	2	Provide a background about the rationale of study, what has been previously done, and why this survey is needed.	Round 2	87.5%<br>4.42 ± 0.83	95.4%	-
Purpose/aim	3	Identify specific purposes, aims, goals, or objectives of the study.	Round 1	95.65%<br>4.78 ± 0.52	-	-
Methods						
Study design	4	Specify the study design in the "Methods" section with a commonly used term (e.g., cross-sectional or longitudinal).	Round 2	86.9%<br>4.26 ± 0.96	86.3%	-
Data collection methods	5a	Describe the questionnaire (e.g., number of sections, number of questions, number and names of instruments used).	Round 2	75%<br>3.88 ± 0.99	77.2%	-
	5b	Describe all questionnaire instruments that were used in the survey to measure particular concepts. Report target population, reported validity and reliability information, scoring/classification procedure, and reference links (if any).	Round 2	78.2%<br>4.00 ± 1.04	72.7%<br>4.055±0.96	-
	5c	Provide information on pretesting of the questionnaire, if performed (in the article or in an online supplement). Report the method of pretesting, number of times questionnaire was pre-tested, number and demographics of participants used for pretesting, and the level of similarity of demographics between pre-testing participants and sample population.	Round 2	79.1%<br>4.08 ± 0.83	86.3%	-
	5d	Questionnaire, if possible, should be fully provided (in the article, or as appendices or as an online supplement).	Round 2	83.3%<br>4.25 ± 0.85	77.2%	-
Sample characteristics	6a	Describe the study population (i.e., background, locations, eligibility criteria for participant inclusion in survey, exclusion criteria).	Round 1	95.5%<br>4.74 ± 0.69	-	-
	6b	Describe the sampling techniques used (e.g., single stage or multistage sampling, simple random sampling, stratified sampling, cluster sampling, convenience sampling). Specify the locations of sample participants whenever clustered sampling was applied.	Round 1	95.8%<br>4.54 ± 0.72	-	-
	6c	Provide information on sample size, along with details of sample size calculation.	Round 1	83.3%<br>4.42 ± 0.88	-	-
	6d	Describe how representative the sample is of the study population (or target population if possible), particularly for population-based surveys.	Round 1	83.3%<br>4.21 ± 0.83	-	-
Survey administration	7a	Provide information on modes of questionnaire administration,	Round 2	91.6%<br>4.33 ± 0.64	86.3%<br>4.33±0.61	-

(*continued on next page*)

Table 1. (continued)

Section/topic	Item	Item description	Item included after which round of Delphi (round 1/round 2/round 3)	Agreement in round 1 (%) Mean score* ± standard deviation	Agreement in round 2 (%) Mean score ± standard deviation	Agreement in round 3 (%) Mean score ± standard deviation
		including the type and number of contacts, the location where the survey was conducted (e.g., outpatient room or by use of online tools, such as SurveyMonkey).				
	7b	Provide information of survey's time frame, such as periods of recruitment, exposure, and follow-up days.	Round 1	100%<br>4.13 ± 0.85	-	-
	7c	Provide information on the entry process:<br>–>For non-web-based surveys, provide approaches to minimize human error in data entry.<br>–>For web-based surveys, provide approaches to prevent "multiple participation" of participants.	Round 2	79.1%<br>4.52 ± 0.51	90.9%	-
Study preparation	8	Describe any preparation process before conducting the survey (e.g., interviewers' training process, advertising the survey).	Round 3	58.3%<br>3.63 ±0.93	61.1%<br>3.83±0.78	77.7%<br>3.83±0.85
Ethical considerations	9a	Provide information on ethical approval for the survey if obtained, including informed consent, institutional review board [IRB] approval, Helsinki declaration, and good clinical practice [GCP] declaration (as appropriate).	Round 2	91.3%<br>4.61 ± 0.89	72.7%<br>4±1.31	-
	9c	Provide information about survey anonymity and confidentiality and describe what mechanisms were used to protect unauthorized access.	Round 1	83.3%<br>4.25 ± 1.07	-	-
Statistical analysis	10a	Describe statistical methods and analytical approach. Report the statistical software that was used for data analysis.	Round 1	95.8%<br>4.58 ± 0.88	-	-
	10b	Report any modification of variables used in the analysis, along with reference (if available).	Round 2	75%<br>4.00 ± 1.14	83.3%<br>4.16±0.71	-
	10c	Report details about how missing data was handled. Include rate of missing items, missing data mechanism (i.e., missing completely at random [MCAR], missing at random [MAR], or missing not at random [MNAR]), and methods used to deal with missing data (e.g., multiple imputation).	Round 2	96.6%<br>4.57 ± 0.73	77.2%<br>4.44±0.81	-
	10d	State how non-response error was addressed.	Round 2	70.8%<br>4.04 ± 0.91	77.2%<br>4.11±0.70	-
	10e	For longitudinal surveys, state how loss to follow-up was addressed.	Round 2	79.1%<br>4.08 ± 1.02	86.3%<br>4.44±0.62	-
	10f	Indicate whether any methods such as weighting of items or propensity scores have been used to adjust for non-representativeness of the sample.	Round 1	83.3%<br>4.17 ± 1.05		-
	10g	Describe any sensitivity analysis conducted.	Round 2	78.2%<br>3.96 ± 0.77	86.3%	-
Results						
Respondent characteristics	11a	Report numbers of individuals at each stage of the study. Consider using a flow diagram, if possible.	Round 1	95.4%<br>4.59 ± 0.59	-	-
	11b	Provide reasons for non-participation at each stage, if possible.	Round 1	77.2%<br>4.05 ± 0.84	-	-
	11c	Report response rate, present the definition of response rate or the formula used to calculate response rate.	Round 1	95.2%<br>4.33 ± 0.73	-	-
	11d	Provide information to define how unique visitors are determined. Report number of unique visitors	Round 1	77.2%<br>4.05 ± 0.84	-	-

(continued on next page)

Table 1. (continued)

Section/topic	Item	Item description	Item included after which round of Delphi (round 1/round 2/round 3)	Agreement in round 1 (%) Mean score* ± standard deviation	Agreement in round 2 (%) Mean score ± standard deviation	Agreement in round 3 (%) Mean score ± standard deviation
		along with relevant proportions (e.g., view proportion, participation proportion, completion proportion).				
Descriptive results	12	Provide characteristics of study participants, as well as information on potential confounders and assessed outcomes.	Round 1	95.2%<br>4.57 ± 0.6	-	-
Main findings	13a	Give unadjusted estimates and, if applicable, confounder-adjusted estimates along with 95% confidence intervals and *p* values.	Round 1	77.2%<br>4.32 ± 0.84	-	-
	13b	For multivariable analysis, provide information on the model building process, model fit statistics, and model assumptions (as appropriate).	Round 1	90.9%<br>4.55 ± 0.8	-	-
	13c	Provide details about any sensitivity analysis performed. If there are considerable amount of missing data, report sensitivity analyses comparing the results of complete cases with that of the imputed dataset (if possible).	Round 2	81.8%<br>4.14 ± 0.83	77.2%<br>4.05±0.70	-
Discussion						
Limitations	14	Discuss the limitations of the study, considering sources of potential biases and imprecisions, such as non-representativeness of sample, study design, important uncontrolled confounders.	Round 1	95.4%<br>4.86 ± 0.47	-	-
Interpretations	15	Give a cautious overall interpretation of results, based on potential biases and imprecisions and suggest areas for future research.	Round 1	95.4%<br>4.59 ± 0.73	-	-
Generalizability	16	Discuss the external validity of the results.	Round 1	90.9%<br>4.45 ± 0.8	-	-
Other sections						
Role of the funding source	17	State whether any funding organization has had any roles in the survey's design, implementation, and analysis.	Round 1	100.0%<br>4.73 ± 0.46	-	-
Conflict of interest	18	Declare any potential conflict of interest.	Round 1	100.0%<br>4.77 ± 0.43	-	-
Acknowledgements	19	Provide names of organizations/persons that are acknowledged along with their contribution to the research.	Round 1	90.9%<br>4.41±0.67	-	-

**Based on Likert scale rating from 1 (strongly disagree) to 5 (strongly agree). Items' scores were re-rated if major modifications were made in the previous round*

Limitations

We acknowledge the limitations of our study. First, the use of the Delphi consensus method may involve some subjectivity in interpreting experts' responses and suggestions. Second, six experts were lost to follow up. Nonetheless, we think our checklist could improve the quality of the reporting of survey studies. Similar to other reporting checklists, CROSS requires to be re-evaluated and revised overtime to ensure it remains relevant and up-to-date with evolving research methodologies of survey studies. We therefore welcome feedback, comments, critiques, and suggestions for improvement from the research community.

CONCLUSIONS

We think CROSS has the potential to be a beneficial resource to researchers who are designing and conducting survey studies. Following CROSS before and during the survey administration could assist researchers to ensure their surveys are sufficiently reliable, reproducible, and transparent.

Acknowledgements: *We are thankful to Dr. David Moher (Ottawa Hospital Research Institute, Canada) and Dr. Masahiro Hashizume (Department of Global Health Policy, Graduate School of Medicine, The University of Tokyo, Tokyo, Japan) for initial contribution of the project and in rating and development of the checklist. We are also grateful to Obaida Istanbuly (Keele University, UK) and Omar Diab (Private*

Dental Practice, Jordan) for their contribution in the earlier phases of the project.

***Corresponding Author:** Nguyen Tien Huy, MD, PhD; Institute of Tropical Medicine (NEKKEN) and School of Tropical Medicine and Global Health, Nagasaki University, Nagasaki 852-8523, Japan (e-mail: tienhuy@nagasaki-u.ac.jp).*

The online version contains supplementary material available at https://doi.org/10.1007/s11606-021-06737-1.

***Author Contribution** NTH is the generator of the idea, and supervised and helped in writing, reviewing, and mediating Delphi process; AS participated in making a draft of guidelines, mediating Delphi process, analysis of results, writing, and process validation; TLT helped in making a draft of guidelines, and analysis; MNT helped in drafting checklist and mediating Delphi process; NNH, NSJ, KSA, and MK helped in writing and mediating Delphi; AM, JK, CLP, JKB, CDW, FJD, MH, YK, EK, JV, GHL, AG, KGT, ML, EMJ, WKL, STS, CDS, BM, SL, UST, MM and MK helped in the rating of items in Delphi rounds and reviewing the manuscript.*

***Funding** None.*

Declarations:

***Conflict of Interest:** The authors declare that they do not have a conflict of interest.*

***Ethics approval:** Ethics approval was not required for the study.*

REFERENCES

1. Wikipedia contributors. (2020). Survey (human research). In *Wikipedia, The Free Encyclopedia*. Retrieved 19:59, December 26, 2020, from https://en.wikipedia.org/w/index.php?title=Survey_(human_research)&oldid=994953597.
2. **Maymone MBC, Venkatesh S, Secemsky E, Reddy K, Vashi NA.** Research Techniques Made Simple: Web-Based Survey Research in Dermatology: Conduct and Applications. J Invest Dermatol 2018;138(7):1456-1462. doi: https://doi.org/10.1016/j.jid.2018.02.032.
3. **Alcock I, White MP, Pahl S, Duarte-Davidson R, Fleming LE.** Associations Between Pro-environmental Behaviour and Neighbourhood Nature, Nature Visit Frequency and Nature Appreciation: Evidence from a Nationally Representative Survey in England. Environ Int 2020;136:105441. doi: https://doi.org/10.1016/j.envint.2019.105441.
4. **Siddiqui J, Brown K, Zahid A, Young CJ**. Current Practices and Barriers to Referral for Cytoreductive Surgery and HIPEC Among Colorectal Surgeons: a Binational Survey. Eur J Surg Oncol 2020;46(1):166-172. doi: https://doi.org/10.1016/j.ejso.2019.09.007
5. **Lee JG, Park CH, Chung H, Park JC, Kim DH, Lee BI, Byeon JS, Jung HY**. Current Status and Trend in Training for Endoscopic Submucosal Dissection: a Nationwide Survey in Korea. PLoS One 2020;15(5):e0232691. doi: https://doi.org/10.1371/journal.pone.0232691
6. **McChesney SL, Zelhart MD, Green RL, Nichols RL**. Current U.S. Pre-Operative Bowel Preparation Trends: a 2018 Survey of the American Society of Colon and Rectal Surgeons Members. Surg Infect 2020;21(1):1-8. doi: https://doi.org/10.1089/sur.2019.125
7. **Núñez A, Manzano CA, Chi C.** Health Outcomes, Utilization, and Equity in Chile: an Evolution from 1990 to 2015 and the Effects of the Last Health Reform. Public Health 2020;178:38-48. doi: https://doi.org/10.1016/j.puhe.2019.08.017
8. **Blackwell AKM, Kosīte D, Marteau TM, Munafò MR**. Policies for Tobacco and E-Cigarette Use: a Survey of All Higher Education Institutions and NHS Trusts in England. Nicotine Tob Res 2020;22(7):1235-1238. doi: https://doi.org/10.1093/ntr/ntz192
9. **Liu S, Zhu Y, Chen W, Wang L, Zhang X, Zhang Y**. Demographic and Socioeconomic Factors Influencing the Incidence of Ankle Fractures, a National Population-Based Survey of 512187 Individuals. Sci Rep 2018;8(1):10443. doi: https://doi.org/10.1038/s41598-018-28722-1
10. **Tamanini JTN, Pallone LV, Sartori MGF, Girão MJBC, Dos Santos JLF, de Oliveira Duarte YA, van Kerrebroeck PEVA**. A Populational-Based Survey on the Prevalence, Incidence, and Risk Factors of Urinary Incontinence in Older Adults-Results from the "SABE STUDY". Neurourol Urodyn 2018;37(1):466-477. doi: https://doi.org/10.1002/nau.23331
11. **Tink W, Tink JC, Turin TC, Kelly M**. Adverse childhood experiences: survey of resident practice, knowledge, and attitude. Fam Med 2017;49(1):7-13
12. **Shi S, Lio J, Dong H, Jiang I, Cooper B, Sherer R.** Evaluation of Geriatrics Education at a Chinese University: a Survey of Attitudes and Knowledge Among Undergraduate Medical Students. Gerontol Geriatr Educ 2020;41(2):242-249. doi: https://doi.org/10.1080/02701960.2018.1468324
13. **Bennett, C., Khangura, S., Brehaut, J. C., Graham, I. D., Moher, D., Potter, B. K., & Grimshaw, J. M.** (2010). Reporting Guidelines for Survey Research: an Analysis of Published Guidance and Reporting Practices. *PLoS Med*, *8*(8), e1001069. https://doi.org/10.1371/journal.pmed.1001069
14. **Turk T, Elhady MT, Rashed S, Abdelkhalek M, Nasef SA, Khallaf AM, Mohammed AT, Attia AW, Adhikari P, Amin MA, Hirayama K, Huy NT**. Quality of Reporting Web-Based and Non-web-based Survey Studies: What Authors, Reviewers and Consumers Should Consider. PLoS One 2018;13(6):e0194239. doi: https://doi.org/10.1371/journal.pone.0194239
15. **Jones, T. L., Baxter, M. A., & Khanduja, V.** (2013). A Quick Guide to Survey Research. *Ann R Coll Surg Engl*, *95*(1), 5–7. https://doi.org/10.1308/003588413X13511609956372
16. **Jones D, Story D, Clavisi O, Jones R, Peyton P.** An Introductory Guide to Survey Research in Anaesthesia. Anaesth Intensive Care 2006;34(2):245-53. doi: https://doi.org/10.1177/0310057X0603400219
17. **Alderman AK, Salem B**. Survey Research. Plast Reconstr Surg 2010;126(4):1381-9. doi: https://doi.org/10.1097/PRS.0b013e3181ea44f9
18. **Moher D, Weeks L, Ocampo M, Seely D, Sampson M, Altman DG, Schulz KF, Miller D, Simera I, Grimshaw J, Hoey J.** Describing Reporting Guidelines for Health Research: a Systematic Review. J Clin Epidemiol 2011;64(7):718-42. doi: https://doi.org/10.1016/j.jclinepi.2010.09.013
19. **Simera, I., Moher, D., Hirst, A.** ***et al.*** Transparent and Accurate Reporting Increases Reliability, Utility, and Impact of Your Research: Reporting Guidelines and the EQUATOR Network. *BMC Med* 8, 24 (2010). https://doi.org/10.1186/1741-7015-8-24
20. **Moher D, Schulz KF, Simera I, Altman DG**. Guidance for Developers of Health Research Reporting Guidelines. PLoS Med 2010;7(2):e1000217. doi: https://doi.org/10.1371/journal.pmed.1000217
21. **Tan WK, Wigley J, Shantikumar S**. The Reporting Quality of Systematic Reviews and Meta-analyses in Vascular Surgery Needs Improvement: a Systematic Review. Int J Surg 2014;12(12):1262-5. doi: https://doi.org/10.1016/j.ijsu.2014.10.015
22. **Grimshaw, J.** (2014). SURGE (The SUrvey Reporting GuidelinE). In Guidelines for Reporting Health Research: a User's Manual (eds D. Moher, D.G. Altman, K.F. Schulz, I. Simera and E. Wager). https://doi.org/10.1002/9781118715598.ch20
23. **Eysenbach G.** Improving the Quality of Web Surveys: The Checklist for Reporting Results of Internet E-Surveys (CHERRIES). J Med Internet Res 2004;6(3):e34. DOI: https://doi.org/10.2196/jmir.6.3.e34.
24. EquatorNetwork.org. Developing your reporting guideline. 3 July 2018 [cited 12/28/2020]; Available from: https://www.equator-network.org/toolkits/developing-a-reporting-guideline/developing-your-reporting-guideline/.
25. **Li AH, Thomas SM, Farag A, Duffett M, Garg AX, Naylor KL**. Quality of Survey Reporting in Nephrology Journals: a Methodologic Review. Clin J Am Soc Nephrol 2014;9(12):2089-94. doi: https://doi.org/10.2215/CJN.02130214
26. **Shankar PR, Maturen KE**. Survey Research Reporting in Radiology Publications: a Review of 2017 to 2018. J Am Coll Radiol 2019;16(10):1378-1384. doi: https://doi.org/10.1016/j.jacr.2019.07.012

27. **Duffett M, Burns KE, Adhikari NK, Arnold DM, Lauzier F, Kho ME, Meade MO, Hayani O, Koo K, Choong K, Lamontagne F, Zhou Q, Cook DJ**. Quality of Reporting of Surveys in Critical Care Journals: a Methodologic Review. Crit Care Med 2012 Feb;40(2):441-9. doi: https://doi.org/10.1097/CCM.0b013e318232d6c6
28. Story DA, Gin V, na Ranong V, Poustie S, Jones D; ANZCA Trials Group. Inconsistent Survey Reporting in Anesthesia Journals. Anesth Analg 2011;113(3):591-5. doi: https://doi.org/10.1213/ANE.0b013e3182264aaf
29. **Marcopulos BA, Guterbock TM, Matusz EF**. [Formula: see text] Survey Research in Neuropsychology: a Systematic Review. Clin Neuropsychol 2020;34(1):32-55. doi: https://doi.org/10.1080/13854046.2019.1590643
30. **Rybakov KN, Beckett R, Dilley I, Sheehan AH.** Reporting Quality of Survey Research Articles Published in the Pharmacy Literature. Res Soc Adm Pharm 2020;16(10):1354-1358. doi: https://doi.org/10.1016/j.sapharm.2020.01.005
31. **Pagano MB, Dunbar NM, Tinmouth A, Apelseth TO, Lozano M, Cohn CS,** Stanworth SJ; Biomedical Excellence for Safer Transfusion (BEST) Collaborative. A Methodological Review of the Quality of Reporting of Surveys in Transfusion Medicine. Transfusion. 2018;58(11):2720-2727. doi: https://doi.org/10.1111/trf.14937
32. **Mulvany JL, Hetherington VJ, VanGeest JB**. Survey Research in Podiatric Medicine: an Analysis of the Reporting of Response Rates and Non-response Bias. Foot (Edinb) 2019;40:92-97. doi: https://doi.org/10.1016/j.foot.2019.05.005
33. **Tabernero P, Parker M, Ravinetto R, Phanouvong S, Yeung S, Kitutu FE, Cheah PY, Mayxay M, Guerin PJ, Newton PN**. Ethical Challenges in Designing and Conducting Medicine Quality Surveys. Tropical Med Int Health 2016 Jun;21(6):799-806. doi: https://doi.org/10.1111/tmi.12707
34. **Keeney S, Hasson F, McKenna H**. Consulting the Oracle: Ten Lessons from Using the Delphi Technique in Nursing Research. J Adv Nurs 2006; 53(2): 205-12 8p. doi: https://doi.org/10.1111/j.1365-2648.2006.03716.x.
35. **Zamanzadeh V, Rassouli M, Abbaszadeh A, Alavi-Majd H, Nikanfar A, Ghahramanian A**. Details of content validity index and objectifying it in instrument development. Nursing Pract Today 2014; 1(3): 163-71.

Publisher's Note: *Springer Nature remains neutral with regard to jurisdictional claims in published maps and institutional affiliations.*
